# Taxonomy of fibroblasts and progenitors in the synovial joint at single-cell resolution

**DOI:** 10.1136/ard-2021-221682

**Published:** 2022-11-22

**Authors:** Fraser L Collins, Anke J Roelofs, Rebecca A Symons, Karolina Kania, Ewan Campbell, Elaina S R Collie-duguid, Anna H K Riemen, Susan M Clark, Cosimo De Bari

**Affiliations:** 1 Arthritis and Regenerative Medicine Laboratory, Centre for Arthritis and Musculoskeletal Health, University of Aberdeen, Aberdeen, UK; 2 Centre for Genome-Enabled Biology and Medicine, University of Aberdeen, Aberdeen, UK

**Keywords:** Fibroblasts, Synovitis, Osteoarthritis, Knee

## Abstract

**Objectives:**

Fibroblasts in synovium include fibroblast-like synoviocytes (FLS) in the lining and *Thy1*+ connective-tissue fibroblasts in the sublining. We aimed to investigate their developmental origin and relationship with adult progenitors.

**Methods:**

To discriminate between *Gdf5*-lineage cells deriving from the embryonic joint interzone and other *Pdgfrα*-expressing fibroblasts and progenitors, adult *Gdf5-Cre;Tom;Pdgfrα-H2BGFP* mice were used and cartilage injury was induced to activate progenitors. Cells were isolated from knees, fibroblasts and progenitors were sorted by fluorescence-activated cell-sorting based on developmental origin, and analysed by single-cell RNA-sequencing. Flow cytometry and immunohistochemistry were used for validation. Clonal-lineage mapping was performed using *Gdf5-Cre;Confetti* mice.

**Results:**

In steady state, *Thy1*+ sublining fibroblasts were of mixed ontogeny. In contrast, *Thy1-Prg4*+ lining fibroblasts predominantly derived from the embryonic joint interzone and included *Prg4*-expressing progenitors distinct from molecularly defined FLS. Clonal-lineage tracing revealed compartmentalisation of *Gdf5*-lineage fibroblasts between lining and sublining. Following injury, lining hyperplasia resulted from proliferation and differentiation of *Prg4*-expressing progenitors, with additional recruitment of non-*Gdf5*-lineage cells, into FLS. Consistent with this, a second population of proliferating cells, enriched near blood vessels in the sublining, supplied activated multipotent cells predicted to give rise to *Thy1*+ fibroblasts, and to feed into the FLS differentiation trajectory. Transcriptional programmes regulating fibroblast differentiation trajectories were uncovered, identifying Sox5 and Foxo1 as key FLS transcription factors in mice and humans.

**Conclusions:**

Our findings blueprint a cell atlas of mouse synovial fibroblasts and progenitors in healthy and injured knees, and provide novel insights into the cellular and molecular principles governing the organisation and maintenance of adult synovial joints.

**WHAT IS ALREADY KNOWN ABOUT THIS TOPIC**

- Synovial fibroblasts, consisting of lining fibroblast-like synoviocytes (FLS) and sublining connective tissue fibroblasts, play a critical role in joint health and arthritis pathology. However, their phenotypic diversity, developmental origin and relationship with adult progenitors is incompletely understood.

**WHAT THIS STUDY ADDS**

- This study reveals the relationship between ontogeny and phenotypic diversity of synovial fibroblasts, and shows at single-cell level the cellular and molecular pathways involved in the response to injury. Findings also identify *Prg4*-expressing FLS progenitors in the lining and facultative progenitors in sublining that are activated by cartilage injury and give rise to FLS and *Thy1*+ sublining fibroblasts.

**HOW THIS STUDY MIGHT AFFECT RESEARCH, PRACTICE OR POLICY**

- This study provides novel insight into the hierarchical pathways and molecular regulation that govern synovial fibroblast cell fate in the adult joint.

## Introduction

The synovium consists of two layers, lining and sublining. The sublining is composed of Thy1+ fibroblasts, immune cells, blood vessels and nerves in a meshwork of extracellular matrix (ECM). The lining consists of type A macrophage-like synoviocytes and type B fibroblast-like synoviocytes (FLS). The FLS are specialised fibroblasts, negative for Thy1, which are unique to the synovium and critical for the maintenance of joint homeostasis through secretion of lubricating factors including lubricin (encoded by *Prg4*) and hyaluronic acid.^1^ Here, we will use the term synovial fibroblasts to collectively refer to Thy1-Prg4+ FLS in the lining and Thy1+ fibroblasts in the sublining.

Synovial fibroblasts express platelet-derived growth factor receptor α (*Pdgfrα*),^2^ a pan-fibroblast marker^3^ also expressed by skeletal progenitors.^4^ They are ontogenetically heterogeneous and derive in part from the growth differentiation factor 5 (*Gdf5)*-expressing cells of the embryonic joint interzone.^2^ The joint-interzone cells give rise to joint tissues during development, including articular cartilage and synovium.^5–7^ Tracing of *Gdf5*-expressing cell progeny into adulthood, using *Gdf5* regulatory sequence to control *Cre* expression that is active in the embryonic knee joint interzone but not in healthy, injured or osteoarthritic adult knees,^8–10^ revealed that the *Gdf5-*lineage cells in adult mouse knees proliferate following cartilage injury and repair cartilage.^2^ More recently, we identified within the adult *Gdf5*-lineage cell population two progenitor cell subsets, *Prg4*-expressing cells in synovial lining and *Sox9*-expressing cells in periosteum, which cooperate to form osteophytes during osteoarthritis.^11^ FLS express *Prg4*, and whether the progenitor activity of *Prg4*+ cells in the lining reflects FLS plasticity or true progenitor cells exist that are distinct from FLS, remains to be determined. Furthermore, it is not known whether a common adult stem/progenitor cell lineage or distinct pools of progenitors supply the different subsets of synovial fibroblasts.

Here, we used transgenic mice allowing the separation of ontogenetically distinct *Gdf5*-lineage mesenchymal stromal cells from other *Pdgfrα*-expressing fibroblasts and progenitors, we analysed at the single-cell level the transcriptome of these lineages in healthy and injured adult knees to construct a stromal cell atlas of the joint and elucidate the relationships between fibroblasts and progenitors in synovium.

## Methods

Materials and methods are available inonline supplemental materials and tables 1–4

10.1136/ard-2021-221682.supp1 Supplementary data

## Results

### Developmental origin and taxonomy of adult synovial fibroblasts in steady state.

**Figure 1:**
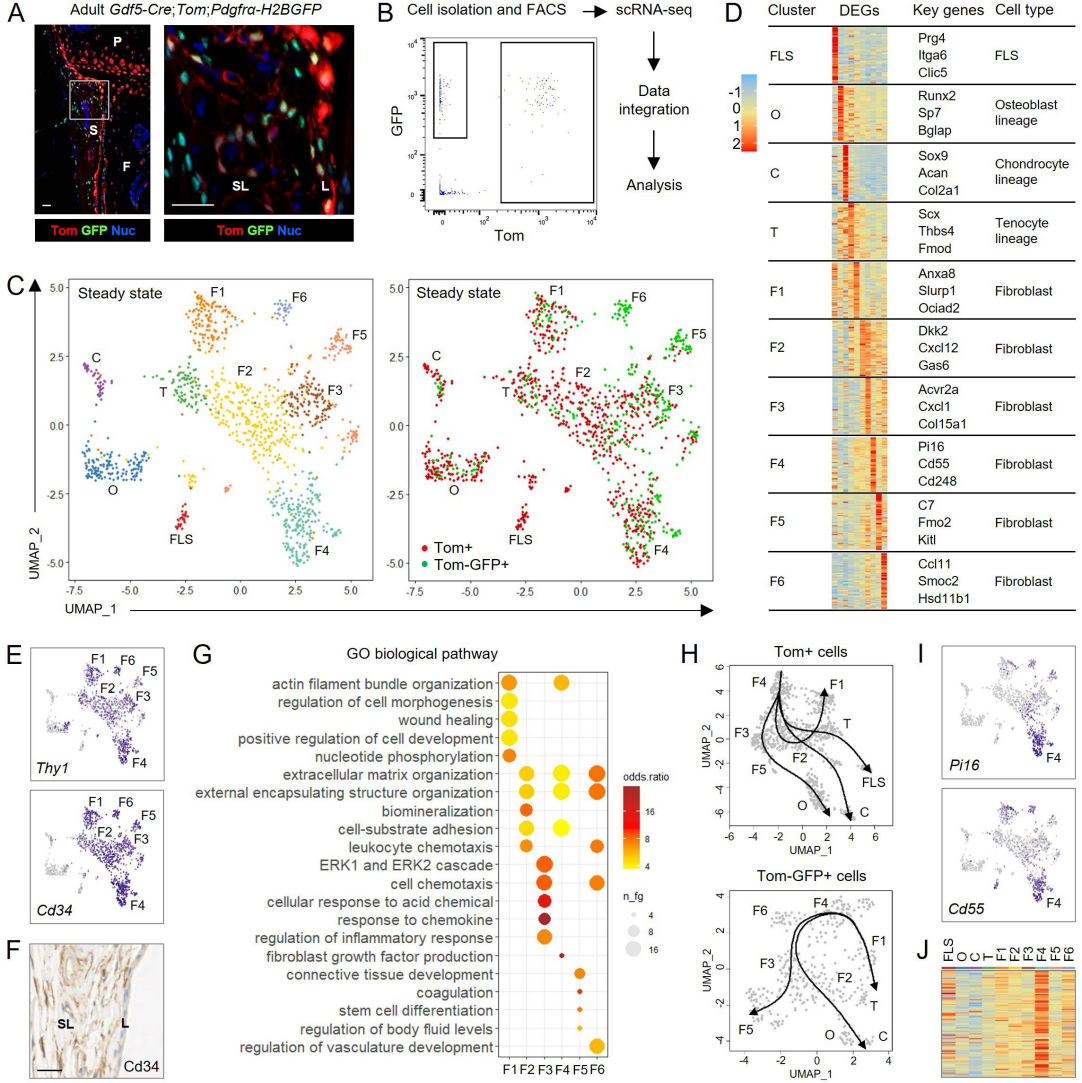
Single-cell transcriptomic atlas of adult mouse synovial fibroblasts. Cells were isolated from knees of 11-week-old *Gdf5-Cre;Tom;Pdgfrα-H2BGFP* mice, sorted by FACS into *Gdf5*-lineage cells (Tom+) and other cells expressing the pan-fibroblast marker *Pdgfrα* (Tom-GFP+), and analysed by scRNA-seq. (A) Histology showing Tom+GFP+ and Tom-GFP+ cells in synovium of 15-week-old *Gdf5-Cre;Tom;Pdgfrα-H2BGFP* mouse knee (n=3). Blue: DAPI nuclear counterstain. White outline on left shown at higher magnification on right. scale bars: 20 µm. S: synovium; P: patella; F: femur; SL: synovial sublining; L: synovial lining. (B) Experimental workflow with flow cytometry scatter plot showing cell populations sorted by FACS. See online supplemental figure 1 for extended data. (C) Unsupervised UMAP plot of integrated scRNA-seq data from 786 Tom+ cells (n=2 mice) and 376 Tom-GFP+ cells (n=1 matched mouse). Left: unsupervised clustering. Right: colour-coded by analysed cell population. See online supplemental figure 2 for extended data. (D) Analysis of differentially expressed genes (DEGs) to identify clusters. Heatmaps show expression of top 50 DEGs for each cluster. Key genes indicate selected DEGs that identify cell types or are dominant cluster-specific genes. See online supplemental figure 3 for UMAP plots of key genes and online supplemental table 5 for top 10 DEGs for each cluster. (E) UMAP plots showing expression of *Thy1* and *Cd34* by fibroblast clusters F1-F6. (F) Immunohistochemical detection of Cd34 in synovial sublining (SL) of 11-week-old mouse knee (n=7). Scale bar: 20 µm. (G) Over-representation analysis of gene ontology (GO) categories for the identified *Thy1*+ fibroblast clusters. (H) Inferred lineage trajectories within the Tom+ and the Tom-GFP+ cell populations based on Slingshot unbiased pseudotime analysis visualised using principal curves. Within both ontogenetic cell lineages, the F4 fibroblast cluster was predicted to represent a root state. (I) UMAP plots showing expression of the cross-tissue universal fibroblast marker *Pi16* and *Cd55* by fibroblast cluster F4. (J) Heatmap showing expression of top 100 DEGs of the *Pi16*+ cross-tissue fibroblast cluster identified by Buechler *et al* 15 by the clusters identified in the adult mouse knee. UMAP, uniform manifold approximation and projection; FLS, fibroblast-like synoviocytes; C, chondrocyte-lineage cells; O, osteoblast-lineage cells; T, tenocyte-lineage cells; F, fibroblasts.

To investigate the developmental origin of adult synovial fibroblasts, we used *Gdf5-Cre;Tom;Pdgfrα-H2BGFP* mice to trace cells from the *Gdf5*-expressing embryonic joint interzone based on tdTomato (Tom) expression and to identify fibroblasts and progenitors based on *Pdgfrα*-promoter-driven green fluorescent protein (GFP) expression (figure 1A). Cells isolated from adult mouse knees were sorted by FACS into Tom+ *Gdf5*-lineage cells, which coexpressed GFP, and Tom-GFP+ cells, and analysed independently by scRNA-seq, to ensure high purity (figure 1B; online supplemental figures 1 and 2). Unsupervised clustering of integrated datasets (figure 1C) and analysis of differentially expressed genes (DEGs) (figure 1D; online supplemental figure 3; table 5) identified FLS, osteoblast-lineage cells, chondrocyte-lineage cells, tenocyte-lineage cells, and 6 fibroblast clusters (F1–F6) expressing the synovial sublining fibroblast markers *Thy1* and *Cd34* (figure 1E, F).^12 13^ Gene Ontology (GO) analysis of significant cluster genes indicated functional diversity between the *Thy1*+ fibroblast clusters (figure 1G). The two ontogenetic lineages made variable contributions to the different fibroblast clusters, and within each cluster, Tom+ and Tom-GFP+ cells were highly transcriptomically similar (online supplemental figure 4). Strikingly, FLS were only detected in the Tom+ population, deriving from the embryonic joint interzone (figure 1C).

To identify putative developmental relationships among cell clusters, we performed unsupervised Slingshot lineage inference.^14^ This predicted, for both ontogenetic cell lineages, trajectories that emerged from the F4 fibroblast cluster towards the specialised cells of the skeletal joint (figure 1H). The transcriptome of the F4 cluster was characterised by *Pi16* and *Cd55* expression (figure 1I), and correlated with the transcriptome of a population of *Pi16*+ fibroblasts recently identified across multiple tissues that has been postulated to represent a reservoir of non-specialised, universal fibroblasts that can develop into specialised, tissue-specific fibroblasts (figure 1J).^15^

Collectively, these data reveal that the adult joint contains functionally distinct fibroblast subsets of heterogeneous developmental origin, with each ontogenetic lineage comprising a universal fibroblast population predicted to give rise to specialised cells.

### Identification of FLS and progenitors in synovial lining

**Figure 2:**
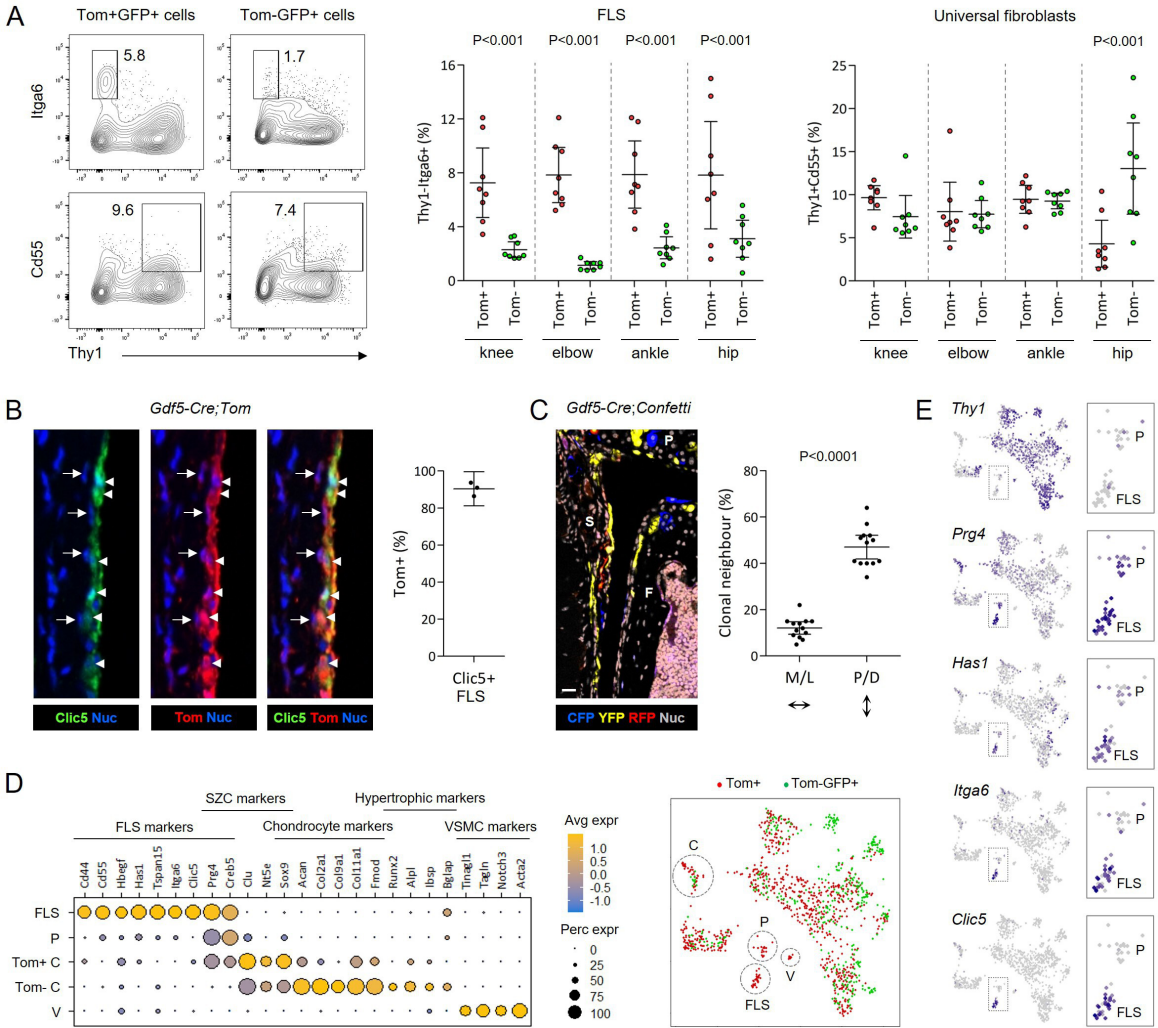
Ontogenetic compartmentalisation of synovial lining and sublining fibroblasts. (A) Detection of Thy1-Itga6+ FLS and Thy1+Cd55+ universal fibroblasts within Tom+GFP+ and Tom-GFP+ cell populations in indicated joints of 21–23 week old *Gdf5-Cre;Tom;Pdgfrα-H2BGFP* mice (n=8) by flow cytometry. Flow plots show gating to identify cell populations. See online supplemental figure 6 for full gating strategy and controls. Graphs show percentage of Thy1-Itga6+ FLS and Thy1+Cd55+ fibroblasts within Tom+GFP+ and Tom-GFP+ cell populations in each of the joints. P values: two-way repeated-measures ANOVA with Holm-Sidak post-test after log-transformation. (B) Tom+cells expressing the FLS marker Clic5 (arrowheads) and adjacent Tom+Clic5- cells (arrows) in synovial lining of 10-week-old *Gdf5-Cre;Tom;Pdgfrα-H2BGFP* mice detected by immunofluorescence staining. Scale bars: 10 µm. Graph shows the percentage of Clic5+ FLS that are Tom+ (n=3). See online supplemental figure 7 for low magnification image and isotype negative control staining. (C) Clonal lineage analysis in 13–16 week old *Gdf5-Cre;Confetti* mouse knees. Clonal cell clusters in synovium, marked by expression of cerulean fluorescent protein (CFP), yellow fluorescent protein (YFP) or red fluorescent protein (RFP), show alignment along the proximal-distal axis parallel to synovial lining. Scale bar: 20 µm. S: synovium; P: patella; F: femur. Graph shows percentage of labelled cells with at least one neighbouring cell expressing the same fluorescent protein in medial or lateral (M/L), or proximal or distal (P/D), direction (n=13; seven unoperated mice and six unoperated contralateral knees from injured mice). P value: paired two-tailed t-test. (D) Expression of selected marker genes to identify subclusters in the steady-state scRNA-seq data, as indicated on the UMAP plot on the right. Cells within a *Prg4*+ progenitor subcluster (P) expressed *Prg4* and *Creb5,* but were largely negative for other FLS and chondrocyte-lineage markers. Tom+ cells in the chondrocyte-lineage cluster (C) displayed a superficial zone chondrocyte (SZC) phenotype, while Tom- cells displayed a mature/hypertrophic chondrocyte phenotype and likely derived from growth plate. An additional Tom+ subcluster was identified as vascular smooth muscle cells (V). See online supplemental figure 8 for extended data. (E) UMAP plots showing expression of indicated genes. Dotted outline indicates enlarged region shown on right to highlight expression in FLS and the *Prg4*+ progenitor subcluster (P). All graphs show mean ± 95% CI. ANOVA, analysis of variance; UMAP, uniform manifold approximation and projection; FLS, fibroblast-like synoviocyte; SZC, superficial zone chondrocytes; VSMC, vascular smooth muscle cells.

Analysis of the scRNA-seq data showed that the FLS cluster only included Tom+ cells deriving from the embryonic joint interzone (figure 1C). We sought to confirm this in a larger cohort of mice and other synovial joints. Tom+ cells were present in synovial lining in all joints analysed (online supplemental figure 5). Flow cytometry confirmed that Thy1-Itga6+ FLS were enriched in the Tom+GFP+ population, while Thy1+Cd55+ universal fibroblasts were similarly or less abundant in the Tom+GFP+ compared with the Tom-GFP+ population (figure 2A; online supplemental figure 6). Furthermore, immunofluorescence staining on tissue sections showed that the vast majority of FLS, identified by Clic5 expression, expressed Tom (figure 2B; online supplemental figure 7). These findings show that, consistent with the scRNA-seq data, the FLS in the adult synovial joints predominantly derive from the embryonic joint interzone.

To define the spatial patterns of adult synovial fibroblasts by their derivation from individual embryonic joint interzone cells, we carried out clonal-lineage mapping using *Gdf5-Cre* mice crossed with Confetti multi-colour reporter mice.^16^ This revealed clonal fibroblast clusters in synovium to be typically aligned longitudinally, along the proximal-distal axis, parallel to the lining (figure 2C). This indicates that parallel clonal cell stacking underpins synovial tissue architecture and suggests that lining fibroblasts are a self-maintaining cell population throughout life.

Interestingly, we observed Tom+ cells in synovial lining that were negative for the FLS marker Clic5 (figure 2B), raising the possibility of the existence of distinct progenitors within the *Gdf5*-lineage synovial lining fibroblast population. Consistent with this notion, we identified in the scRNA-seq data a Tom+ subcluster of *Thy1-Prg4*+ cells, which were distinct from mature FLS (defined by expression of *Cd44*, *Cd55*, *Hbegf*, *Has1*, *Tspan15*, *Itga6* and *Clic5*)^17^ and superficial zone chondrocytes^18–21^ (figure 2D and E; online supplemental figure 8, table 6). We additionally identified growth plate chondrocytes and vascular smooth muscle cells (figure 2D; online supplemental figure 8, table 6), and a putative progenitor subset within the osteoblast-lineage cluster (online supplemental figure 9). These findings further define the mouse synovial joint stromal cell atlas and identify *Prg4*-expressing synovial lining cells distinct from FLS.

### Activation of synovial fibroblasts following joint injury

**Figure 3:**
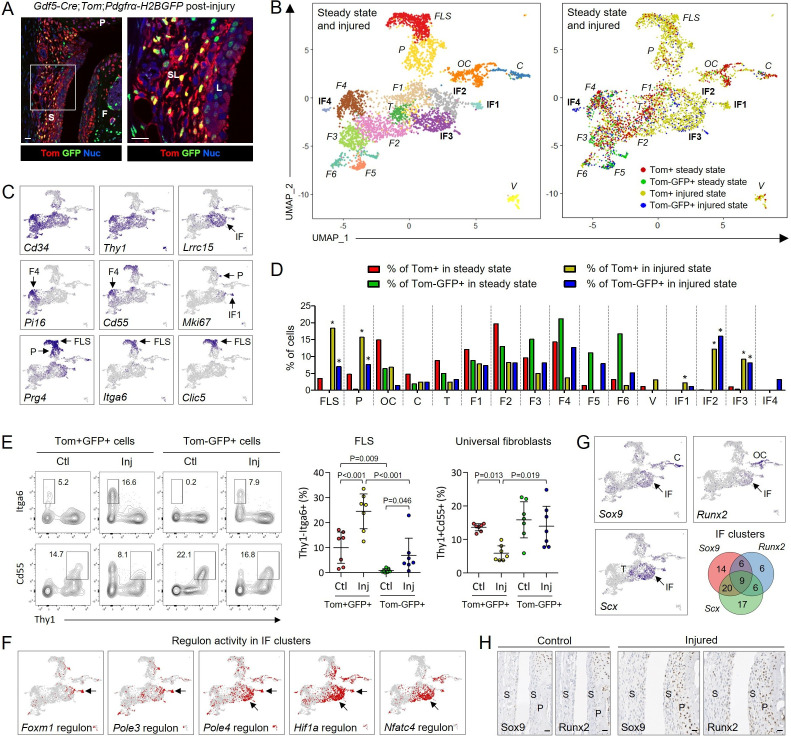
Single-cell transcriptomic atlas of synovial fibroblasts from 12-week-old *Gdf5-Cre;Tom;Pdgfrα-H2BGFP* mice 6 days after joint surface injury, integrated with steady-state data shown in figure 1. (A) Histology showing Tom+GFP+ and Tom-GFP+ cells in synovium 6 days after injury (n=3). Blue: DAPI nuclear counterstain. White outline on left shown at higher magnification on right. Scale bars: 20 µm. S: synovium; P: patella; F: femur; SL: synovial sublining; L: synovial lining. (B) UMAP plot of integrated scRNA-seq data from 786 steady-state Tom+ cells (n=2 mice), 376 steady-state Tom-GFP+ cells (n=1 matched mouse), 2383 injured-state Tom+ cells (n=4 mice) and 651 injured-state Tom-GFP+ cells (n=2 matched mice). Left: unsupervised clustering. Right: colour-coded by analysed cell population and state. Injury-induced fibroblast (IF) clusters are in bold; clusters with steady-state analogues in italics. See online supplemental figure 10–12 for extended data. (C) Expression of key genes that identify clusters. See online supplemental figure 13 for extended data. (D) Relative abundance of cells across identified cell clusters. *FDR<0.05 vs steady-state, negative binomial generalised linear model with Benjamini-Hochberg post-test. (E) Detection by flow cytometry of Thy1-Itga6+ FLS and Thy1+Cd55+ universal fibroblasts within the Tom+GFP+ and Tom-GFP+ cell populations in control (Ctl) and injured (Inj) knees of 15–19 week old *Gdf5-Cre;Tom;Pdgfrα-H2BGFP* mice 6 days post-injury (n=7). See online supplemental figure 14 for gating strategy and controls. Graphs show percentage of cells within the respective cell populations that are Thy1-Itga6+ FLS or Thy1+Cd55+ fibroblasts. Lines and error bars: mean ± 95% CI. P values: two-way repeated-measures ANOVA with Holm-Sidak post-test. (F) Regulons active in injury-induced fibroblast clusters IF1-3. (G) Expression of lineage-specifying transcription factors. Venn diagram shows percentage of cells in injury-induced clusters IF1-IF3 expressing *Sox9*, *Runx2* and *Scx*. See online supplemental figure 17 A, B for other cell clusters. (H) Immunohistochemical detection of Sox9 and Runx2 in synovium (S) and periosteum (P) in consecutive tissue sections of control and injured knees of 11–13 week old mice 7 days post injury (n=5). Scale bars: 20 µm. ANOVA, analysis of variance; UMAP, uniform manifold approximation and projection; FDR, false discovery rate; C, chondrocyte-lineage cells; F, fibroblasts; FLS, fibroblast-like synoviocytes; IF, injury-induced fibroblasts; OC, osteochondral-lineage cells; T, tenocyte-lineage cells; VSMC, vascular smooth muscle cells.

To study fibroblast activation, we used a mouse model whereby injury to articular cartilage triggers a healing response characterised by fibroblast proliferation that underpins synovial hyperplasia and chondrogenesis to repair cartilage.^2^ We analysed cells 6 days after cartilage injury, a time when synovial hyperplasia peaks (figure 3A),^2^ and integrated the data with steady-state data (figure 3B; online supplemental figure 10). Clusters identified by unsupervised clustering were annotated by analysing the top DEGs for each cluster and mapping cells from each steady-state cluster onto the integrated UMAP (figure 3C; online supplemental figures 11-13; table 7). Relative abundance analysis revealed increases in FLS and *Prg4*+ progenitor populations post-injury within both ontogenetic lineages (figure 3D), and FLS expansion was confirmed by flow cytometry (figure 3E; online supplemental file 14). The injured-state Tom+ and Tom-GFP+ FLS were transcriptomically highly similar to each other and to the steady-state Tom+ FLS (online supplemental figure 15). Transcriptomic comparisons between steady-state and injured-state cells within the fibroblast clusters revealed upregulation of genes involved in ECM remodelling and fibroblast migration, such as *Cthrc1*, *Postn*, *Timp1*, *Bgn*, *Lum*, *Sparc*, *Lox* and various collagens (online supplemental figure 16).^13 22–24^

Four injury-induced fibroblast (IF) clusters were identified with no steady-state analogous cluster (figure 3B–D; online supplemental figures 11-13), which were characterised by activity of regulons associated with cell proliferation and activation (figure 3F; online supplemental tables 8-12). Individual cells in these clusters coexpressed *Sox9* (chondrocyte-lineage), *Runx2* (osteoblast-lineage) and *Scx* (tenocyte-lineage) transcription factors (figure 3G; online supplemental figure 17A, B), suggestive of multilineage differentiation potential. In addition, the cluster analogous to the osteoblast-lineage cluster in steady state displayed an osteochondral phenotype after injury (online supplemental figure 17C, D), similar to the hybrid skeletal cells that form the early osteophyte in osteoarthritis.^11^ Immunostaining confirmed upregulation of Sox9 and Runx2 expression after injury, especially at the joint margin where synovium and periosteum merge and chondrophyte formation is typically observed (figure 3H).

Altogether, these data indicate that injury triggers expansion of the *Prg4*+ progenitor and FLS populations, which in part involves recruitment of cells that do not derive from the *Gdf5*-expressing joint interzone, and induces activation of fibroblasts expressing genes indicative of multi-lineage differentiation potency.

### Context-dependent activated fibroblast phenotypes

Next, we sought to determine the specificity of the synovial fibroblast response to cartilage injury. Recently, Buechler *et al* analysed single-cell transcriptomic data of fibroblasts from multiple injured or diseased mouse tissues and identified three perturbed-state fibroblast (PF) populations.^15^ Two of these showed transcriptomic similarity to the injury-induced clusters in our study (online supplemental figure 18).

**Figure 4:**
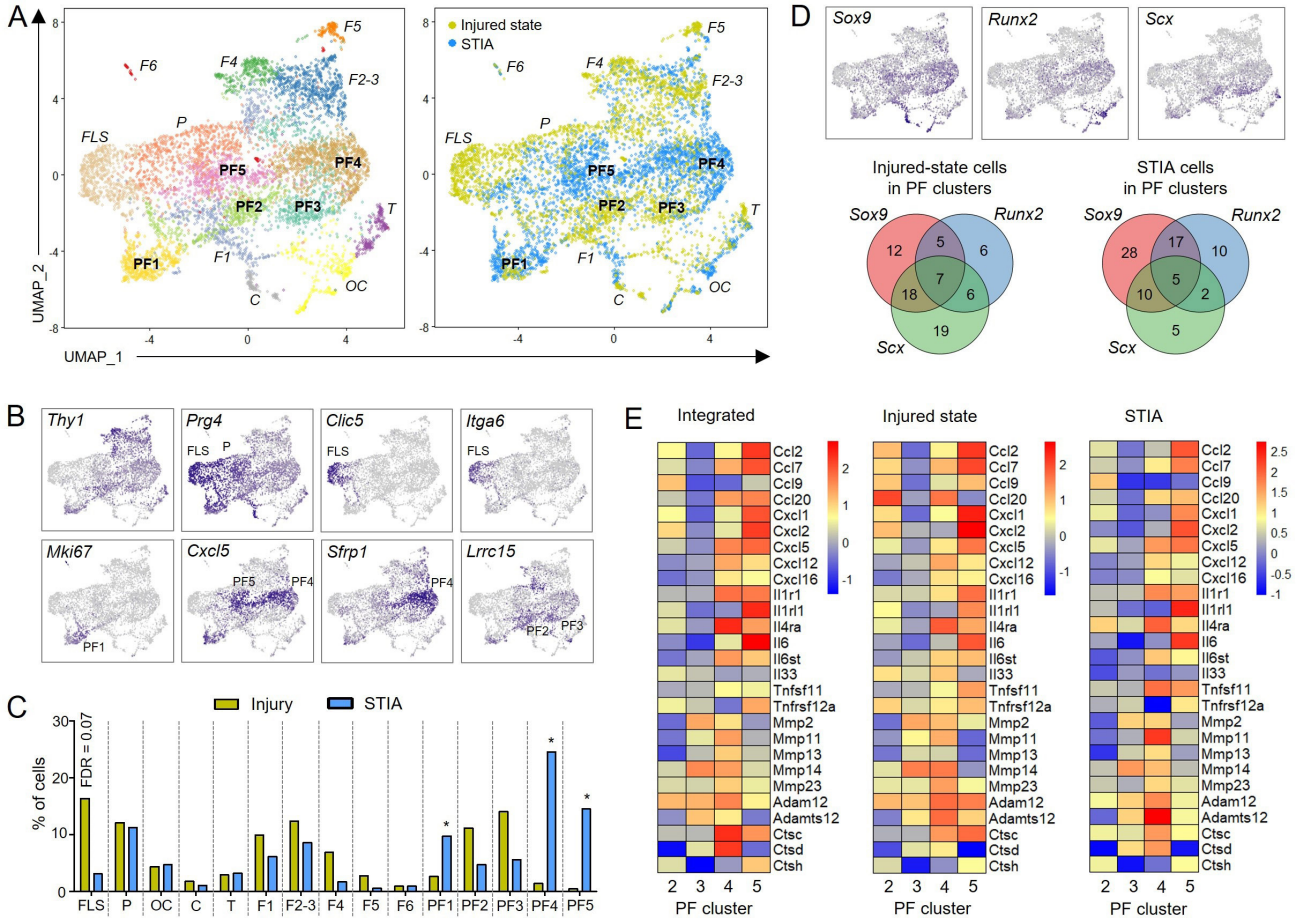
Integrated single-cell transcriptomic analysis of synovial fibroblasts from mice 6 days after joint surface injury (data shown in figure 3) and day 9 serum-transfer induced arthritis (STIA) mice.^25^ (A) UMAP plot of integrated scRNA-seq data from 2955 Tom+ and Tom-GFP+ injured-state cells and 3549 STIA cells. Left: unsupervised clustering. Right: colour-coded by state. See online supplemental figures 19, 20 for cluster annotation. Perturbed-state fibroblast (PF) clusters are in bold; clusters with steady-state analogues in italics. (B) Expression of key genes that identify steady-state analogous clusters (top) and perturbed-state clusters (bottom). (C) Relative abundance of cells across identified clusters. *FDR<0.05 vs injured-state, negative binomial generalised linear model with Benjamini-Hochberg post-test. (D) Expression of lineage-specifying transcription factors. Venn diagrams show percentage of STIA cells and injured-state cells in perturbed-state clusters (PF1-PF5) expressing *Sox9*, *Runx2* and *Scx*. (E) Heatmaps showing expression of differentially expressed genes (DEGs) involved in inflammation and catabolism in non-proliferating perturbed-state fibroblast clusters (PF2-PF5). Left: integrated injured-state and STIA cells. Middle: Injured-state cells only. Right: STIA cells only. UMAP, uniform manifold approximation and projection; FDR, false discovery rate.

We then focused on a comparison with fibroblasts from joints of mice with serum transfer-induced inflammatory arthritis (STIA), by integrating our injured-state dataset with the STIA dataset published by Croft *et al*.^25^ Unsupervised clustering revealed five perturbed-state fibroblast clusters (PF1-5) (figure 4A–C; online supplemental figures 19, 20), which showed expression of lineage-specifying transcription factors (*Sox9*, *Runx2* and *Scx*) in both models (figure 4D). Strikingly, few FLS were present in STIA, while *Thy1-Prg4*+ cells extended from the progenitor cluster into the perturbed-state clusters (figure 4A–C).

PF clusters included one proliferating cluster (PF1), and four clusters predominant in either the injured state (PF2 and PF3) or STIA (PF4 and PF5) (figure 4A–C), the latter characterised by *Cxcl5* expression (figure 4B). Cells in STIA-dominant PF clusters showed a more inflammatory and catabolic transcriptome compared with cells in injury-dominant PF clusters, and this remained true when injured-state and STIA cells were analysed separately (figure 4E).

These findings indicate that different perturbed-state synovial fibroblast phenotypes exist, in varying proportions, both after injury and during immune-mediated inflammation, and suggest that adoption of multilineage potency by fibroblasts is a generic response to a perturbed state.

### Identification and molecular regulation of progenitor cell differentiation trajectories

**Figure 5:**
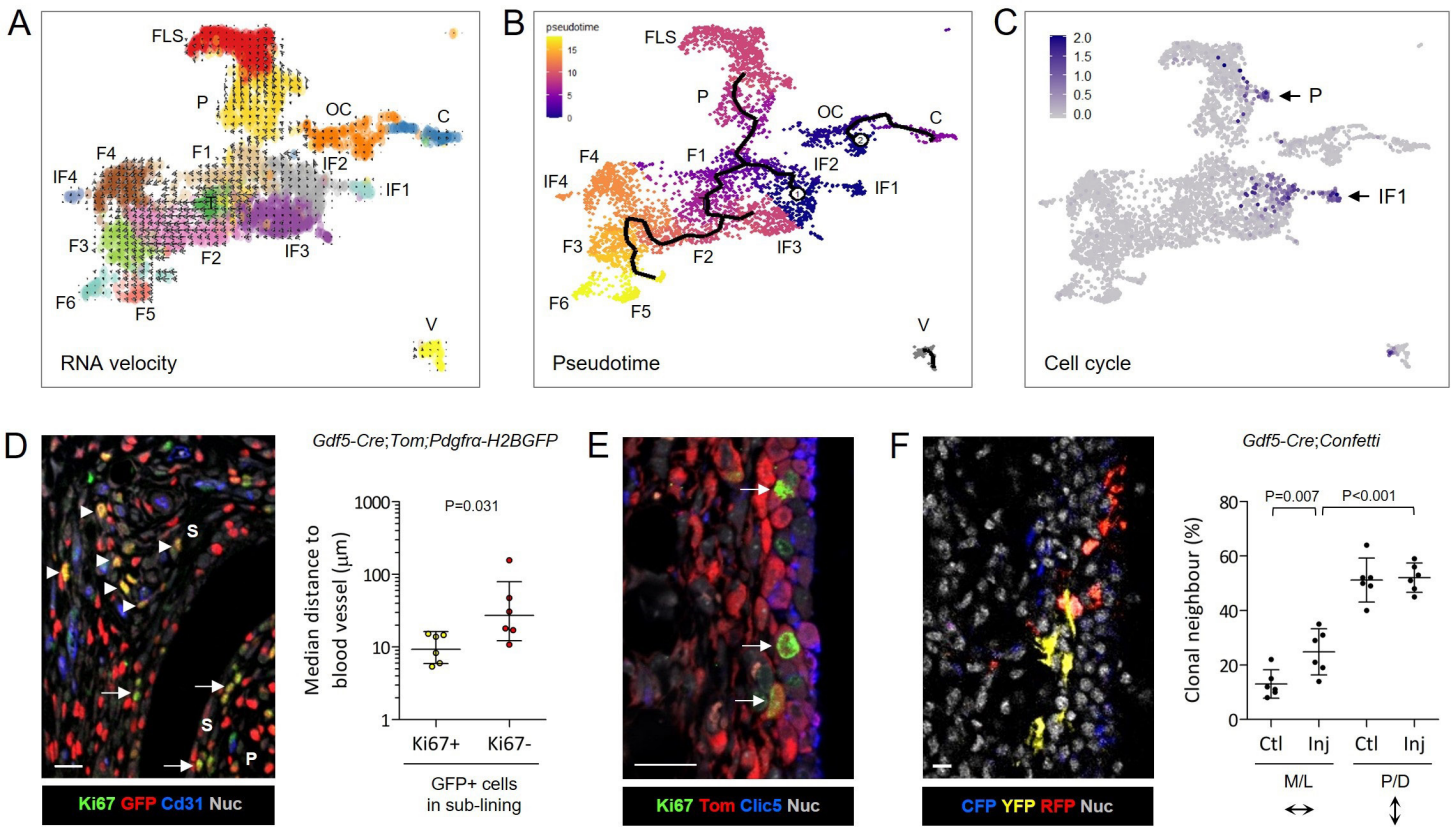
Stromal cell proliferation and differentiation trajectories after injury. (A) RNA velocity analysis using the dynamical model projected onto the integrated steady-state and injured-state uMAP plot as shown in figure 3B. Arrows show the local average velocity and direction. (B) Monocle3 lineage inference showing differentiation trajectories originating from injury-induced fibroblast clusters with a branchpoint towards *Thy1*+ fibroblasts or FLS. Colouring of plot represents pseudotime. (C) UMAP plot of cell cycle gene module score based on expression of *Mki67*, *Ccna2*, *Ccnb1*, *Ccnb2* and *Cdk1*. Arrows indicate proliferating cells. See online supplemental figure 21 for separate analysis of injured-state Tom+, injured state Tom-GFP+, and steady-state populations. (D, E) Immunofluorescence staining in 12–14 week old *Gdf5-Cre;Tom;Pdgfrα-H2BGFP* mouse synovium 6 days after injury (n=6) to locate proliferating fibroblasts. (D) Ki67+GFP+ proliferating fibroblasts in synovial lining (arrows), and near Cd31+blood vessels in synovial sublining (arrowheads). See online supplemental figure 22A for split channel images and isotype negative control staining. Graph shows the median distance to the nearest Cd31+ blood vessel for Ki67+GFP+ proliferating fibroblasts and Ki67-GFP+ non-proliferating fibroblasts in synovial sublining. Lines and error bars indicate geometric mean ± 95% CI. P value: Wilcoxon signed rank test. (E) Ki67+Tom+ proliferating *Gdf5*-lineage cells in synovial lining (arrows), adjacent to Clic5+Tom+ FLS. see online supplemental figure 22B for split channel images and isotype negative control staining. (F) Clonal lineage analysis in 13–14 week old *Gdf5-Cre;Confetti* mouse knees 6 days after injury (n=6). Ctl: unoperated contralateral control knees. Cerulean fluorescent protein (CFP), yellow fluorescent protein (YFP), red fluorescent protein (RFP) and TO-PRO-3 nuclear counterstain were detected by confocal fluorescence microscopy. Graph shows percentage of labelled cells with at least one neighbouring cell expressing the same fluorescent protein in the medial or lateral (M/L), or the proximal or distal (P/D), direction. Lines and error bars indicate mean ± 95% CI. P value: two-way repeated-measures ANOVA with Holm-Sidak post-test. ANOVA, analysis of variance; UMAP, uniform manifold approximation and projection.

We next analysed differentiation trajectories using RNA velocity and lineage reconstruction^14 26 27^ (figure 5A) and by pseudotemporally ordering the cells based on changes in gene expression using Monocle 3^27^ (figure 5B). This revealed inferred differentiation trajectories originating from cells in injury-induced fibroblast clusters IF1 and IF2, with a branchpoint towards either *Thy1-Prg4*+ lining fibroblasts (P and FLS) or *Thy1*+ sublining fibroblasts (F1–F6 and tenocytes) (figure 5A and B). Cell cycle analysis revealed two clusters of cycling cells after injury supplying new cells feeding into the differentiation trajectories, one in IF1 and IF2 clusters, and one in the *Thy1-Prg4*+ progenitor cluster extending into the FLS cluster (figure 5C; online supplemental figure 21).

To confirm these findings in situ, we costained for GFP and the proliferation marker Ki67, together with the endothelial marker Cd31. Proliferating fibroblasts were detected in lining, and in sublining enriched near blood vessels (figure 5D, online supplemental figure 22A). To further determine the identity of proliferating fibroblasts in the lining, we costained for Tom and Ki67, together with the FLS marker Clic5. We observed Tom+Ki67+ proliferating cells in the lining located immediately adjacent to Clic5+ FLS (figure 5E, online supplemental figure 22B), and occasional Tom+Ki67+Clic5+ FLS (online supplemental figure 22B), supporting the notion that the *Thy1-Prg4*+ progenitors identified by scRNA-seq (P cluster) are located in the lining where they proliferate and give rise to new FLS after injury. Clonal-lineage tracing using the *Gdf5-Cre;Confetti* model indicated clonal expansion along the medial-lateral axis (figure 5F), although clones typically remained locally confined to either lining or sublining compartments. Altogether, these data indicate that synovial lining hyperplasia after injury in large part results from proliferation of *Gdf5*-lineage FLS progenitors in the lining, with additional recruitment from proliferating cells in injury-induced clusters into the FLS trajectory.

**Figure 6:**
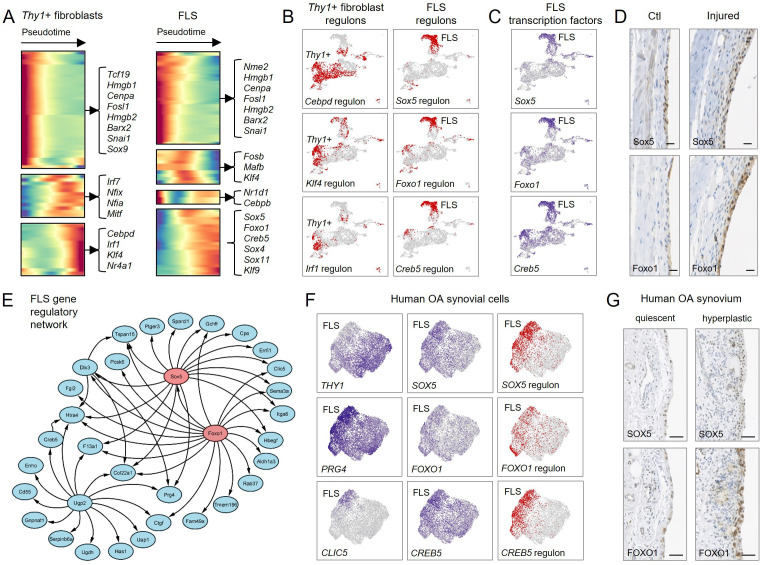
Transcriptional programmes regulating fibroblast differentiation trajectories. (A–C) Transcription factors and regulons associated with the two fibroblast differentiation trajectories (*Thy1*+ fibroblasts and FLS) identified in figure 5. (A) Heatmaps showing expression across pseudotime of transcription factors whose expression changed significantly across either trajectory. Transcription factors are clustered by pseudotemporal expression pattern. Top differentially expressed transcription factors are indicated. (B) Activity of selected regulons associated with transcription factors identified by pseudotime analysis, projected onto the integrated steady-state and injured-state UMAP plot as shown in figure 3B. See online supplemental figure 23 for regulon activity analysed independently in steady state and injured state. (C) Expression of selected FLS-associated transcription factors identified from the pseudotime analysis projected onto the integrated steady-state and injured-state UMAP plot. Expression of *Sox5* and *Foxo1* is mostly restricted to the FLS, while *Creb5* shows a wider expression pattern. (D) Immunohistochemical detection of SOX5 and FOXO1 in near-consecutive tissue sections of 11–13 week old mice 7 days post-injury (n=5) showing expression in synovial lining of control (unoperated contralateral control knee) and injured knees. Scale bars: 20 µm. See online supplemental figure 24A for lower magnification images and isotype negative control stainings. (E) Predicted FLS-associated gene regulatory network based on the mouse scRNA-seq data, constructed using GENIE3 and Cytoscape, indicating that the transcription factors Sox5 and Foxo1 drive key FLS-associated genes. (F) UMAP plots of scRNA-seq data from human OA synovium obtained at arthroplasty (n=3; 2 female and one male, mean age 68)^28^ showing from left to right expression of indicated marker genes to identify FLS (THY1-PRG4+CLIC5+), expression of transcription factors *SOX5*, *FOXO1 and CREB5*, and activity of associated regulons. See online supplemental figure 25 for analysis of two additional human OA synovial cell datasets.^13 29^ (G) Immunohistochemical detection of SOX5 and FOXO1 in near-consecutive tissue sections of human OA synovium obtained at arthroplasty (n=6; see online supplemental table 1 for donor information), showing expression in quiescent and active areas of synovial lining. Scale bars: 20 µm. See online supplemental figure 24B for lower magnification images and isotype negative controls. UMAP, uniform manifold approximation and projection; FLS, fibroblast-like synoviocytes; OA, osteoarthritis.

To gain insight into the molecular regulation of synovial fibroblast differentiation, we identified transcription factors that significantly changed in expression across pseudotime (figure 6A). SCENIC analysis revealed regulon activity associated with these transcription factors along their respective differentiation trajectories, suggesting they are key to driving this process (figure 6B; online supplemental figure 23; tables 13-18). FLS-associated transcription factors included *Sox5*, *Foxo1 and Creb5* (figure 6C), with *Sox5* and *Foxo1* detectable by immunostaining in the lining of normal and injured knee synovium (figure 6D; online supplemental figure 24A). Reconstruction of gene regulatory networks revealed that *Sox5* and *Foxo1* transcription factors interact with key FLS genes (figure 6E), supporting their potentially critical role in FLS fate determination.

For clinical relevance, we extended our analysis to published data from knee synovial tissues of osteoarthritis patients,^13 28 29^ which similarly showed that *THY1-PRG4+CLIC5*+ FLS exhibited *SOX5, FOXO1* and *CREB5* regulon activity (figure 6F, online supplemental figure 25). Immunohistochemistry confirmed SOX5 and FOXO1 expression in both quiescent and hyperplastic lining of human synovium (figure 6G, online supplemental figure 24B). These data indicate that molecular regulation of the FLS phenotype is conserved across species and states.

## Discussion

Comprehensive cell atlases from diseased joints with inflammatory or degenerative arthritis have documented heterogeneity of synovial fibroblasts, identifying perturbed-state subsets.^13 15 25 30^ We previously reported that in the adult knee synovium, the *Gdf5*-lineage cell population contains fibroblasts that become pathogenic in inflammatory arthritis^31^ and progenitors that form cartilage after injury.^2 11^ However, little was known about the fibroblasts in healthy joints, and it remained to be determined whether progenitors and fibroblasts are distinct cells, or plastic fibroblasts adopt progenitor activity. In this study, single-cell transcriptomic analysis of ontogenetically distinct stromal cell lineages from steady-state mouse knee joints led to the identification of FLS and distinct *Prg4*-expressing progenitors in the lining, both largely deriving from the embryonic joint interzone. Joint surface injury, employed to study repair mechanisms,^2^ triggered proliferation of progenitors in the lining, and additional cells located near blood vessels in sublining predicted to supply specialised fibroblasts.

Traditionally, *Prg4*-expressing synovial lining fibroblasts are considered to be specialised FLS that maintain joint homeostasis through secretion of lubricating factors.^1^ Here, we disentangle the identity of the lining fibroblasts and show that they comprise two distinct cell subsets, FLS and progenitors postulated to replenish FLS lost to physiological turnover. The observation that in synovium, clonal fibroblasts are arranged longitudinally parallel to the lining, supports this notion. We also show that the synovial lining hyperplasia following joint surface injury^2 32^ is largely underpinned by an expansion of FLS driven by proliferating *Prg4*-expressing progenitors. A previous study tracing the progeny of cells expressing *Prg4* showed their proliferation and expansion in synovium after cartilage injury.^32^ Our data identify a population of *Prg4*-expressing progenitors in synovial lining that are distinct from FLS and respond to injury with proliferation to supply new FLS.

The FLS population is further expanded after injury by differentiation of cells that do not derive from the *Gdf5*-lineage population. Although the non-*Gdf5* lineage FLS are transcriptomically highly similar to their *Gdf5*-lineage counterparts, to which extent they are functionally equivalent remains to be determined. Similarly, while *Gdf5*-lineage cells are the main progenitors that form articular cartilage during development, repair cartilage after injury in adulthood,^2^ and form osteophytes in osteoarthritis,^11^ other cells can give rise to new chondrocytes, especially ectopically in synovium after injury.^2^ Thus, while the *Gdf5*-lineage cells are the natural progenitors for FLS and articular chondrocytes, under conditions of stress, other cells in the joint supply FLS and chondrocytes in a compensatory mechanism.

The quiescent cells from which the injury-induced cells with multipotent phenotype originate remain to be determined. A recent study identified a population of fibroblasts that reside near blood vessels in many tissues, marked by expression of *Pi16*. These cells were postulated to be unspecialised reservoir cells giving rise to tissue-specific specialised fibroblasts.^15^ We identified a transcriptomically similar *Pi16*+ fibroblast cluster in the adult mouse knee, which was predicted to give rise to specialised cells of the steady-state skeletal joint. After injury, proliferating cells feeding into differentiation trajectories were found to be enriched in a sublining perivascular niche, and we previously showed these perivascular cells to be distinct from pericytes.^33^ While these cells could be progeny of the *Pi16*+ fibroblasts, we speculate that quiescent fibroblasts in the joint, under the stress resulting from damage, would be opportunistically recruited to function as facultative progenitors, showing a plasticity that has been reported in other tissues.^34^

A comparative analysis of synovial fibroblasts in joint surface injury and various perturbed states revealed an overall similar fibroblast response. Fibroblasts with an inflammatory phenotype were detected in our injury model, although at a much lower prevalence compared with the STIA mouse model of inflammatory arthritis. Inflammation plays a crucial role in repair.^35^ Likewise, activated cells with a multipotent phenotype were detected in both injury and STIA models. These data suggest that inflammatory and multipotent fibroblast transcriptional states reflect a generic response to insult, although their prevalence and level of activation would be context-dependent and likely to determine structural outcome. It was interesting to observe that while few FLS were present in the STIA dataset, *Prg4*-expressing progenitors were abundant and extended into the PF clusters. This suggests that under inflammatory conditions such as rheumatoid arthritis, *Prg4*-expressing progenitors are shifted towards pathogenic fibroblasts.

Our data define the molecular identity of FLS distinct from *Prg4*+ progenitors, and reveal the transcriptional programmes underpinning synovial fibroblast differentiation. Notably, both mouse and human FLS are characterised by *Sox5*, *Foxo1* and *Creb5* regulon activity. This suggests that the identified gene regulatory networks are crucial for the FLS phenotype and that their disruption could result in dysregulation of FLS formation or function. The transcription factors Foxo1 and Sox5 have been linked to skeletal cell survival and fate.^36 37^ Creb5 was shown to be required for the induction of *Prg4* expression in articular chondrocytes.^19^ Since the synovial lining shares many properties with the superficial zone of the articular cartilage, including production of lubricin (encoded by *Prg4*), it is likely that the *Creb5* regulon has similar functions in FLS and superficial zone chondrocytes.

In summary, our analysis at single-cell resolution of stromal cells isolated from steady-state and injured mouse knees provides novel insights into the ontogeny and taxonomy of fibroblast and progenitor populations in synovium and defines differentiation trajectories and their molecular regulation. This study critically advances our knowledge of the cell populations that maintain the synovial joint in adult life.

## Data Availability

Data are available in a public, open access repository. Data are available on reasonable request. Single-cell RNA sequencing data that support the findings of this study have been deposited in Gene Expression Omnibus (GEO) with the accession code GSE214500. All data relevant to the study are included in the article or uploaded as online supplemental information.
