# Supplementary material for: Taxonomy of fibroblasts and progenitors in the synovial joint at single-cell resolution
Source: Ann Rheum Dis. 2022 Nov 22;82(3):428–37. doi: 10.1136/ard-2021-221682 (PMC9933170; doi:10.1136/ard-2021-221682)
Supplement: Supplementary data [file ard-2021-221682supp001.pdf]

Supplementary Materials for

The taxonomy of fibroblasts and progenitors in the synovial joint at single-cell resolution

Authors:

Fraser L. Collins[1#*], Anke J. Roelofs[1#], Rebecca A. Symons[1], Karolina Kania[1], Ewan Campbell[2], Elaina S. R. Collie-Duguid[2], Anna H. K. Riemen[1], Susan M. Clark[1], Cosimo De Bari[1*]

Author Affiliations:

[1]Arthritis & Regenerative Medicine laboratory, Aberdeen Centre for Arthritis and Musculoskeletal Health, Institute of Medical Sciences, University of Aberdeen, Aberdeen AB25 2ZD.

[2]Centre for Genome-Enabled Biology and Medicine, University of Aberdeen, Aberdeen AB25 2ZD.

[#]Contributed equally

Correspondence:

Cosimo De Bari, Institute of Medical Sciences, University of Aberdeen, Foresterhill, Aberdeen AB25 2ZD, UK. Tel: +44-1224-437477, E-mail: c.debari@abdn.ac.uk.

Fraser Collins, Institute of Medical Sciences, University of Aberdeen, Foresterhill, Aberdeen AB25 2ZD, UK, E-mail: fraser.collins@abdn.ac.uk

This PDF file includes:

Methods

Suppl. Figs. 1-25

Tables 1 - 18

Methods

Human tissue collection

Human synovial tissue samples were obtained from patients with a clinical diagnosis of osteoarthritis after informed consent, under the auspices of the NHS Grampian Biorepository, during knee arthroplasty. Patient information is provided in Supplementary Table 1.

Mice

All animal experimental protocols were approved by the UK Home Office and the Animal Welfare and Ethical Review Committee of the University of Aberdeen. All animal experiments were performed at the University of Aberdeen Medical Research Facility after a minimum acclimatisation period of 6 days. Experiments were designed to ensure that minimum numbers of mice were used to obtain biologically significant results. *Gdf5-Cre* mice (Tg(Gdf5-cre-ALPP)1Kng)[1] were donated by Dr David Kingsley (Stanford, CA, USA). *Cre*-inducible *tdTomato* (*Tom*) (B6.Cg-Gt(ROSA)26Sortm14(CAG-tdTomato)Hze/J) reporter mice,[2] *Cre*-inducible *Confetti* (STOCK Gt(ROSA)26Sortm1(CAG-Brainbow2.1)Cle/J) reporter mice[3] and *Pdgfrα-H2BGFP* (B6.129S4-Pdgfratm11(EGFP)Sor/J) mice were obtained from The Jackson Laboratory. *Gdf5-Cre* mice were maintained on an FVB background, *Tom* and *Pdgfrα-H2BGFP* mice on a C57BL/6 background, and Confetti mice on a partly backcrossed C57BL/6 background. Mice within each experiment were maintained in the same temperature-controlled room on a 12h:12h light-dark cycle and provided with food and water *ad libitum*. Female *Gdf5-Cre*;*Tom;Pdgfrα-H2BGFP* mice or *Gdf5-Cre*;*Confetti* mice with a hemizygous or heterozygous transgene status were used for experiments. Mice that showed widespread (leaky) *Tom* expression were excluded *a priori* via detection of Tom expression in peripheral blood.[4] Mice whose genotype at the time of analysis did not correspond with the genotype determined *a priori* were excluded. Mice were either left unoperated (steady-state), or underwent joint surface injury on one knee (injured) with the contralateral knee left unoperated (control), as detailed below. No formal randomisation procedure was performed. Mice were humanely killed at 10-23 weeks of age by overdose of CO_2 followed by cervical dislocation and knee joint dissection. No randomisation was carried out for this study. Donor-matched samples (injured and contralateral knees, or cell populations) were compared. Blinding was not applicable to this study as mice within each experiment were all the same genotype.

Joint surface injury

Surgery was performed to create a joint surface injury as previously described.[4,5] Briefly, mice were anaesthetized with ketamine (50 mg/kg) and medetomidine (0.67 mg/kg) with atipamezole (1 mg/kg) post-operatively, or isoflurane with 0.1 mg/kg buprenorphine and in some experiments paracetamol (200 mg/kg) mixed with soft food for two days post-surgery. Medial parapatellar arthrotomy was

performed on the right knee under a dissection microscope. Using a 25G needle, a full-cartilage-thickness scratch was made along the exposed cartilage of the trochlear groove following lateral dislocation of the patella. The patella was re-located and incisions were closed by suturing. For some experiments, the unoperated contralateral knee served as control knee. Mice were humanely killed 6 or 7 days after surgery.

Tissue processing and histology

Mouse knee joints for histology were PFA-fixed, EDTA-decalcified, and paraffin or cryosectioned, as described.[4] Fluorescent proteins were detected either by their native fluorescence in cryosections, or via immunofluorescence staining on paraffin sections. Immunohistochemistry and immunofluorescence stainings were performed as described[6] using antibodies in Supplementary Table 2. For immunofluorescence detection of Clic5, after incubation with primary antibody against Clic5, tissue sections were sequentially incubated with biotinylated anti-rabbit antibody, streptavidin-AF647 (ThermoFisher, cat. no. S32357), biotinylated anti-streptavidin antibody, and again streptavidin-AF647, for signal amplification. Images were acquired on a Zeiss Axioscan Z1 slide scanner or Zeiss 710 META Laser Scanning Confocal microscope. Image analysis was performed using Zen v3 software. Cell counting was performed manually using ImageJ 1.47v or Zen 2.1 Lite software on at least 3 tissue sections per sample.

Isolation of synovial joint cells

Isolation of cells from mouse synovial joints was performed as previously described.[4] Briefly, dissected joints were incubated with 1 mg/ml collagenase type IV in culture media (high-glucose DMEM with 10% foetal bovine serum (FBS;) and 100 U/ml penicillin and 0.1 mg/ml streptomycin) for 50 minutes at 37°C under agitation. Cells were disassociated by vortexing and cell suspensions passed through a 40 µm cell strainer. Cells were centrifuged at 300g for 5 minutes and resuspended in 1 ml of culture media before further processing and analysis.

Immunophenotyping by flow cytometry

Cells were stained with antibodies listed in Supplementary table 3. Data were acquired on a BD Fortessa flow cytometer and analysed using FlowJo v10 software. Unstained and single-labelled cells or antibody-labelled UltraBeads (eBioscience) were used to set compensation and gates were applied based on fluorescence-minus-one (FMO) controls. Erythrocytes and debris were excluded based on forward and side scatter parameters. Doublets and aggregates were gated out based on forward-scatter parameters. Staining with Fixable Viability Dye eFluor 455UV (eBioscience, cat. no. 65-0868-18) was used to exclude dead cells as indicated. See Supplementary Fig. 6 for gating strategies.

Fluorescence-activated cell sorting

Cells isolated from the knees of steady-state or injured female *Gdf5-Cre*;*Tom;Pdgfrα-H2BGFP* mice at 11-13 weeks of age were sorted for Tom+ (*Gdf5*-lineage) and Tom-GFP+ (non-*Gdf5*-lineage, *Pdgfrα*-expressing) cells on an BD Influx Cell Sorter. Cells from each mouse were sorted and processed independently. Erythrocytes and debris were excluded based on forward and side-scatter profiles. Doublets and aggregates were gated out based on forward-scatter parameters, and non-viable cells excluded by 4',6-diamidino-2-phenylindole (DAPI) staining. Cells were sorted based on Tom and GFP fluorescence into separate FBS-coated tubes containing 1 ml culture media to maintain cell viability. Immediately following sorting, small aliquots of sorted cells were analysed on a BD Fortessa flow cytometer to confirm sample purity. Cells were kept on ice in the dark, for a maximum period of 2 hours, until further processing.

Generation of scRNA-seq data

Following sorting, Tom+ cells (n=2) and donor-matched Tom-GFP+ cells (n=1) from steady-state mice, and Tom+ cells (n=4) and donor-matched Tom-GFP+ cells (n=2) from mice 6 days after joint surface injury, were captured independently using the 10x Genomics Chromium system. Steady-state and injured mice were age- and sex-matched. Generation of data was performed separately for each mouse and across 3 experiments as detailed in Suppl. Table 4. Sequencing libraries were generated using the 10x Genomics Single Cell 5' Library and Gel Bead Kit (version 2) and sequenced on the Illumina NextSeq 500. Alignment and quantification of sequencing data was performed using the 10x Genomics Cell Ranger pipeline (version 2.2.0) and mouse reference genome (GRCm38.p6) obtained from NCBI and modified to contain the *tdTom* and *H2BGFP* transgenes.[7]

Analysis of scRNA-seq data

Quality control of scRNA-seq data was performed using the default parameters of the SoupX package[8] to estimate and remove ambient RNA. Analysis of scRNA-seq data was performed using the Seurat R package (version 4.03) and associated SeuratWrappers for the Velocyto, scVelo and Slingshot packages.[9–13] For samples obtained from steady-state mice, cells with fewer than 200 genes, more than 4000 genes, or greater than 5% mitochondrial reads were excluded from analysis. For samples obtained after injury, cells with fewer than 200 genes, more than 6000 genes, or greater than 5% mitochondrial reads were excluded from analysis. Gene expression measurements were normalised by the total expression, multiplied by a scale factor (10,000) and log-transformed. Cells expressing either haematopoietic cell markers *Ptprc* or *Fcer1g* that were also negative for the fibroblast marker *Pdpn* were subset out from all samples to remove haematopoietic contamination.

Cells expressing *Tom* were subset out from the Tom-GFP+ samples to remove Tom+ cell contamination.

Datasets were integrated using the reciprocal PCA (RPCA) method using 2000 integration anchor features that were repeatedly variable across datasets. To mitigate cell cycle heterogeneity, cell cycle scoring, and regression were performed using the Seurat CellCycleScoring function to calculate G2M and S phase scores. The difference between these scores was used to regress out signal associated with cell cycle.

Fifty principal components were determined to account for the majority of variation and, along with the previously identified 2000 variable genes, were used for uMAP and clustering analysis (original Louvain algorithm). For steady-state and integrated steady-state and injured datasets clustering resolution was set to 0.8. For analysis of individual experimental conditions or comparison of ontogenetic lineages within steady-state or injured-state knees, data were subset from the main integrated file and re-clustered at a resolution of 0.7. Following clustering, clusters corresponding to adipocytes, based on high *Adipoq* expression, were removed using the subset function.

Cluster markers were calculated using the non-parametric Wilcoxon rank sum test with Bonferroni correction using all features in the dataset. Only genes detected in 25% of cells either within or outside the cluster of interest and that demonstrated a minimum log-fold difference of 0.25 were tested for differential expression.

To map steady-state clusters onto the fully integrated dataset we first obtained the barcodes for cells that constituted each steady-state cluster using the Seurat WhichCells function. These barcodes were mapped onto the fully integrated dataset using the Seurat Dimplot function with cells.highlight specified.

Gene Ontology analysis

For gene set over-representation analysis, the R package ‘gsfisher’ was utilised. Cluster-specific gene universes were constructed as those genes expressed by 25% of the cells in the cluster or 25% of all cells. Cluster-specific differentially expressed genes were tested against these gene universes for gene ontology (GO) category enrichments using one-sided Fisher’s exact tests with multiple testing correction, with ‘biological process’ gene sets obtained from the GO database. Results were filtered to discard GO categories with less than 3 genes or more than 500 genes in the foreground list, or an over-representation odds ratio of less than 2.

Pseudo-bulk analysis

For pseudo bulk analysis, average gene expression was calculated using the Seurat AverageExpression function. Briefly, for each cluster, gene expression log values were averaged then scaled so that gene variance across clusters is 1. Correlation between cluster gene expression was performed using the R pairs function with Pearson correlation coefficient.

Differential abundance analysis

For differential abundance analysis, the contribution of each replicate / sample to cluster composition was calculated. Differential abundance was calculated by the negative binomial generalised linear model, using the R package edgeR.[14] Briefly, data were normalised using the relative log expression (RLE) method and design matrix specified. Dispersion was estimated and estimation of quasi-likelihood (QL) dispersions performed using the glmQLFit function. Comparisons between cell types was made using the glmQLFTest function. To control the false discovery rate (FDR), multiple testing correction was performed using the Benjamini-Hochberg method.

Venn diagram generation

For analysis of cells expressing multiple master regulator genes, cells expressing selected genes were identified using the Seurat WhichCells function and passed to the R package VennDiagram.

Single-cell gene regulatory network analysis

For single-cell gene regulatory network analysis we utilised the SCENIC package,[15] using the standard R pipeline. Briefly, the expression matrix was isolated from the Seurat object and loaded into GENIE3 for building the initial co-expression gene regulatory network (GRN). RcisTarget was used to analyse the regulon data along with the mm9-tss-centred-10kb (mouse) or hg19-500bp-upstream-7species (human) database to create transcription factor (TF) motifs. Regulon activity scores were calculated using the AUCell package. A binary output for each regulon (active or inactive) was determined by setting a threshold based upon the ranked distribution of AUCell scores across cells. Regulons were then ranked within clusters for level of activity, and the highest ranked regulons that displayed cluster-specificity were identified as potentially important for regulating cell phenotype. Visualisation of gene regulatory networks was performed using Cytoscape 3 and associated R package RCy3.[16]

MA plot analysis

For MA plot generation, gene log sums were first calculated using the R log2 function and Seurat GetAssayData function. Calculation of gene differential expression was performed via the Seurat FindMarkers function with logfc.threshold set to 0 using the Wilcoxon rank sum test. P-value

adjustment was performed using Bonferroni correction based on the total number of genes in a dataset. Gene log sum was plotted against avg_log2FC using ggplot2. Genes with greater than two-fold change and adjusted p value <0.01 were highlighted.

RNA velocity analysis

The package scVelo was utilised for the analysis of RNA velocity.[11] Briefly, loom files were generated from the 10x Genomics cellranger output files using the velocyto python implementation,[10] loaded into Seurat and converted into a Seurat object. Files were merged and the scTransform command run to normalise, scale and find variable features. Fifty principal components were used. Data was subset based on barcode ID to match cell identities between RNA velocity and the standard Seurat analysis. uMAP co-ordinates from the standard Seurat analysis were used to ensure comparable projections. Data was converted to .h5ad format and loaded into the scVelo package. Analysis of RNA velocity in the scVelo package was performed using the default settings for the dynamical model with differential kinetics.[11]

Pseudotime lineage inference

Single-cell pseudotime trajectories were computed using the Slingshot[13] and Monocle3[12] algorithms. Pre-computed cell embeddings and clusters from the Seurat pipeline served as input.

For Slingshot analysis of ontogenetically separated steady-state datasets, no start or end clusters were defined. Lineage inference was performed by the construction of a minimum spanning tree (MST) between clusters (nodes). Pseudotime was visualised by fitting principal curves to these lineages.[13]

For Monocle 3 analysis of injured datasets, gene expression data was isolated from the Seurat object using the Seurat GetAssayData function and a Monocle cell data set (cds) created using the Monocle3 new_cell_data_set function. The cds was pre-processed, dimensions reduced, and cells clustered using the Monocle3 preprocess_cds, reduce_dimension and cluster_cells functions. Seurat cluster IDs and uMAP settings were then imported into the monocle cds. Trajectories were determined and cells ordered in pseudotime using the Monocle3 learn_graph and order_cells functions. Identification of genes whose expression changed significantly along trajectories, as a function of pseudotime, was determined using graph-auto correlation analysis using the Monocle3 choose_cells and graph_test functions. Selected genes, listed as being transcription factors in the AnimalTFDB3.0 database,[17] that varied significantly in their pseudo-temporal expression pattern (Moran's I > 0.1 and q value < 0.01) were plotted on a heatmap of expression over pseudotime.

Cell cycle analysis

For analysis of cell cycle score, a gene module containing Cdk1, Ccna2, Ccnb1, Ccnb2 and Mki67 was generated and the average expression level in each cluster calculated using the Seurat AddModuleScore function.

Analysis of scRNA-seq data from the mouse serum transfer-induced arthritis (STIA) model

Mouse serum transfer-induced arthritis (STIA) scRNA-seq datasets were obtained from NCBI GEO GSE129087.[18] Cells with fewer than 200 genes, more than 6000 genes, or greater than 5% mitochondrial reads were excluded from analysis. Gene expression measurements were normalised by the total expression, multiplied by a scale factor (10,000) and log-transformed.

For the identification of annotated clusters in the Croft *et al*. study[18] the dataset was processed in a comparable manner. Immune cells were removed and the STIA dataset was down sampled to consist of 1,725 cells. Thirty principal components were used for uMAP generation and clustering performed at a resolution of 0.4. Fibroblast clusters were identified according to the Croft *et al*. study[18] based on the expression of: F1 – *Sfrp2*, *Col11a1*, *Mfap4*; F2 – *Tnfaip6*, *Inhba*, *Prg4*; F3 – *Apod*, *Clec3b*, *Cd34*; F4 – *Top2a*, *Hmgb2*, *Cdk1*; F5 – *Clic5*, *Col22a1*, *Tspan15*. Identification of cells in these clusters was determined using the Seurat WhichCells function and mapped onto the integrated STIA and injured dataset using the Seurat Dimplot function with cells.highlight specified.

For the integration of the STIA and injured datasets we used the reciprocal PCA (RPCA) method using 2000 integration anchor features that were repeatedly variable across datasets. To mitigate cell cycle heterogeneity, cell cycle scoring and regression were performed using the Seurat CellCycleScoring function to calculate G2M and S phase scores. The difference between these scores was used to regress out signal associated with cell cycle. Fifty principal components were determined to account for the majority of variation and, along with the previously identified 2000 variable genes, were used for uMAP and clustering analysis (original Louvain algorithm). Clustering resolution was set to 0.8. Non-fibroblasts were removed based on the expression of *Ptprc*, *Pecam1*, *Myh11* and *Adipoq*.

Analysis of scRNA-seq data from human osteoarthritis patients

Human osteoarthritis scRNA-Seq datasets were obtained from Chou *et al*., NCBI GEO GSE152805 (n = 3 patients),[19] Mizoguchi *et al*., NCBI GEO GSE109449 (n = 2 patients),[20] and Zhang *et al*., ImmPort SDY998 (n = 3 patients).[21] For the Chou *et al*.[19] dataset, cells with fewer than 200 genes, more than 7,500 genes or greater than 30% mitochondrial reads were excluded from analysis, as per original publication.[19] For the Mizoguchi *et al*.[20] dataset, cells with fewer than 200 genes, more than 16,000 genes or greater than 20% mitochondrial reads were excluded from analysis. For the Zhang *et*

al.[21] dataset, cells with fewer than 1000 genes or greater than 25% mitochondrial reads were excluded from analysis, as per original publication.[21] Patient samples from within each study were integrated using the reciprocal PCA (RPCA) method with 2000 repeatedly variable integration anchor features. Cell cycle effects were regressed out and fifty principal components used for uMAP and clustering analysis (original Louvain algorithm, resolution set to 0.4, 0.5 and 0.5 respectively). Haematopoietic, endothelial and smooth muscle cells were excluded based on the expression of PTPRC, PLVAP and ACTA2. Regulon analysis was performed using the SCENIC algorithm as previously described.

Statistical analysis

All data points on graphs, and n-numbers in text, indicate individual human donors or mice, except for graphical visualisations of scRNA-seq data. SigmaPlot v14 and GraphPad Prism v5 software were used for statistical analysis. Tests used to determine statistical significance ($p<0.05$) are indicated in figure legends. Normality and equality of variance were tested in Sigmaplot using the Shapiro-Wilk and Brown-Forsythe tests, respectively. Log-transformation was used to equalise variance prior to statistical testing as indicated.

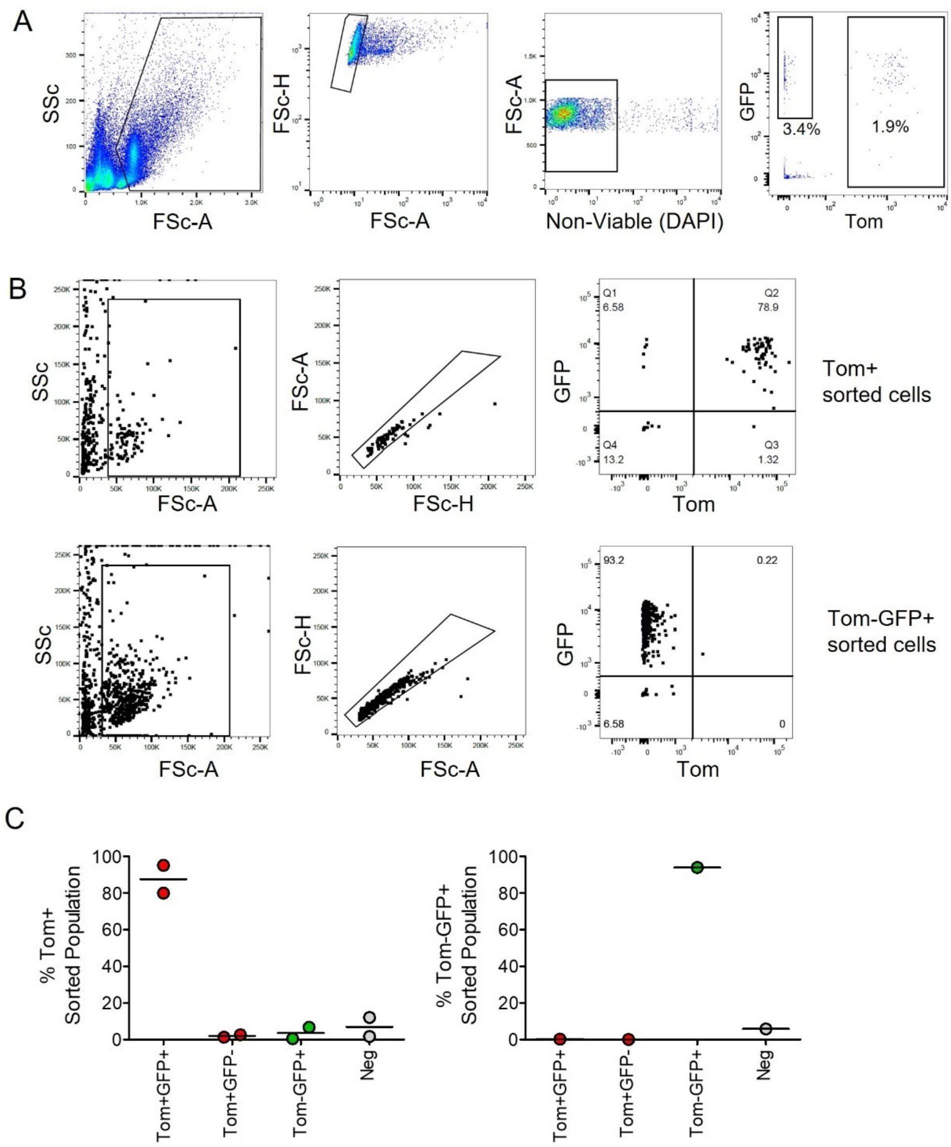

Supplementary Figure 1. Extended data Figure 1B. Cell sorting strategy for isolation of adult *Gdf5*-lineage and non-*Gdf5*-lineage *Pdgfrα*-expressing cells. (**A**) Gating strategy to sort Tom+ (*Gdf5*-lineage) and Tom-GFP+ (non-*Gdf5*-lineage *Pdgfrα+*) cells within single live cells freshly isolated from knees of adult *Gdf5-Cre*;*Tom*;*Pdgfrα-H2BGFP* mice using a BD Influx cell sorter. Erythrocytes and debris were gated out based on Forward and Side Scatter profile. Doublets and aggregates were excluded based on Forward Scatter parameters. Dead cells were excluded based on DAPI staining. Tom+ and Tom-GFP+ cells were sorted based on fluorescence. (**B,C**) Following sorting, aliquots of Tom+ and Tom-GFP+ collected cells were analysed on a BD Fortessa flow cytometer to confirm cell purity. Data in (**C**) show the purity of sorted cells used for scRNA-seq analysis. Note that while Tom+ cells were sorted regardless of GFP expression, the vast majority of Tom+ cells also expressed GFP.

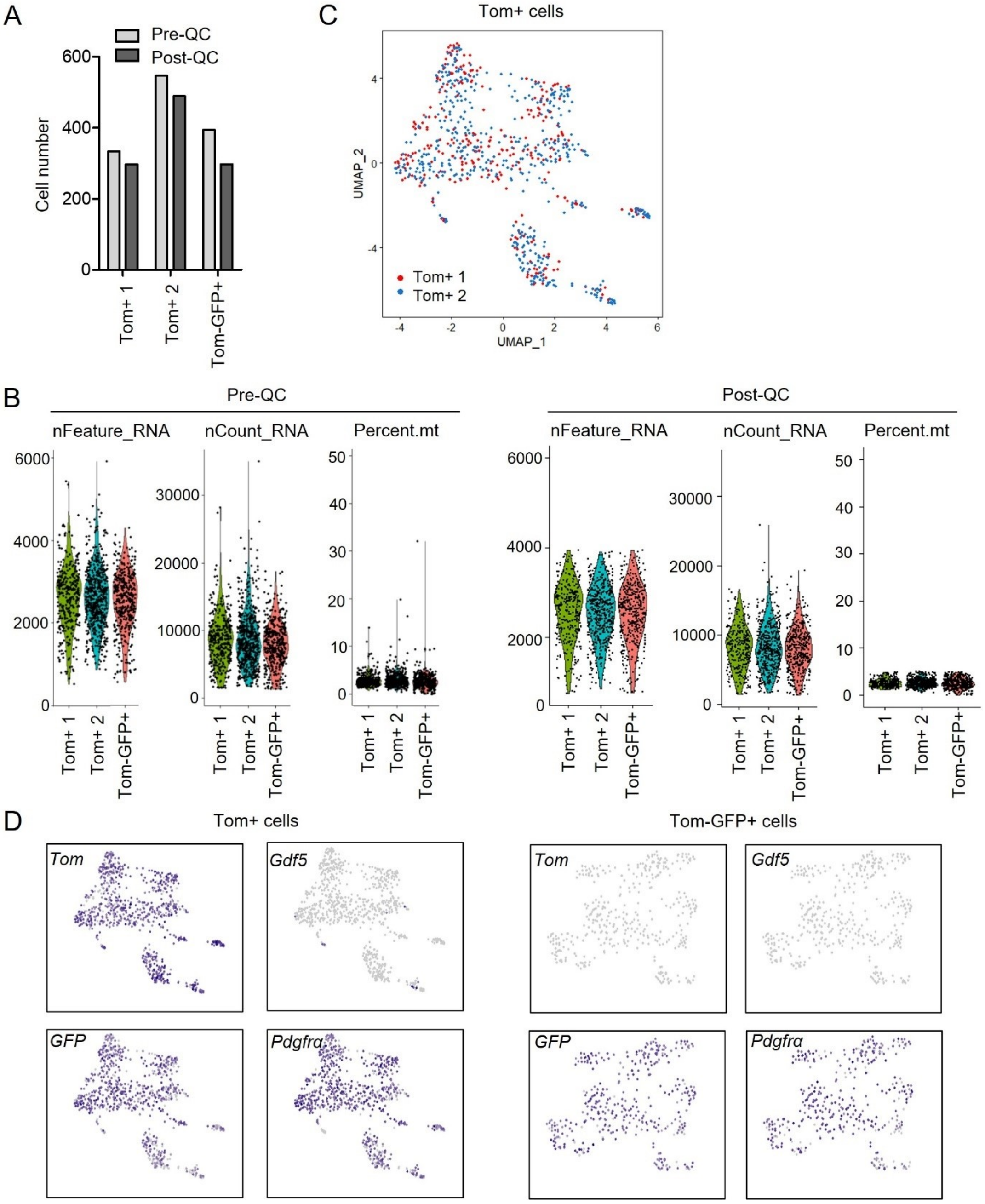

Supplementary Figure 2. Extended data Figure 1C. Single-cell RNA-sequencing analysis of cells isolated from knee joints of adult *Gdf5-Cre;Tom;Pdgfrα-H2BGFP* mice. scRNA-seq data of Tom+ (n=2 mice) and donor-matched Tom-GFP+ (n=1 mouse) sorted cells was obtained using the 10x Genomics Chromium system. (**A**) Number of cells present in each sample before and after QC processing. (**B**) Features, counts and percent mitochondrial genes before and after QC processing. (**C**) Unsupervised uMAP of the Tom+ cells coloured by biological replicate (mouse). (**D**) Detection of *Tom, Gdf5, GFP* and *Pdgfrα* expression in the analysed cell populations.

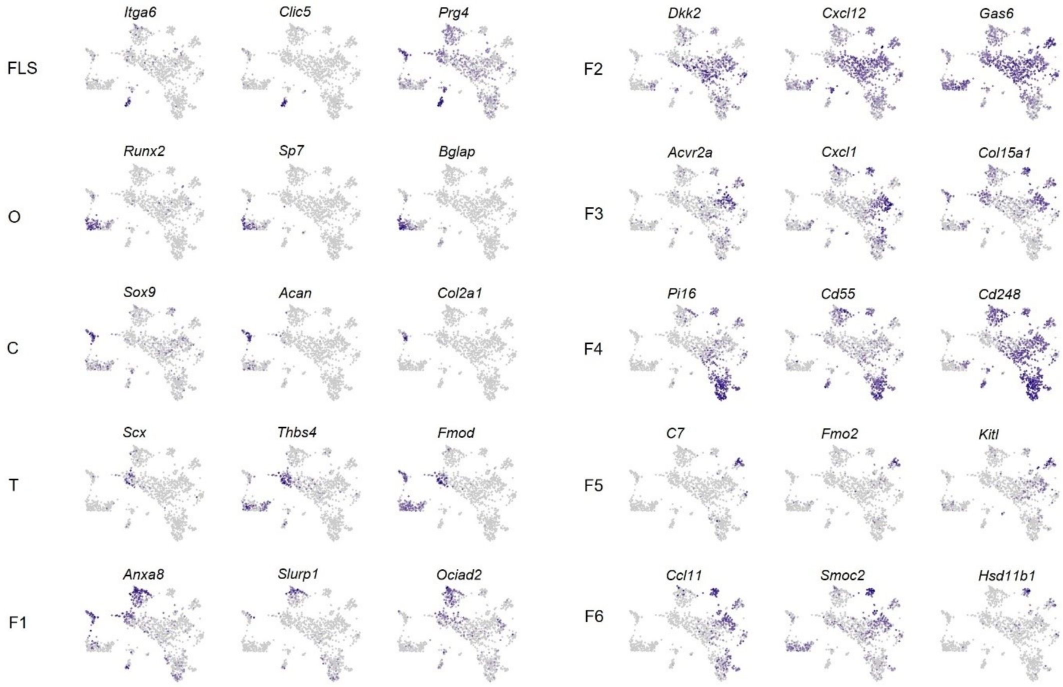

Supplementary Figure 3. Extended data Fig. 1D. UMAP plots showing the expression of selected DEGs for each cluster that identify specialised cell types or are dominant cluster-specific genes. FLS: Fibroblast-like synoviocytes; O: Osteoblast-lineage cells; C: Chondrocyte-lineage cells; T: Tenocyte-lineage cells; F: Fibroblasts.

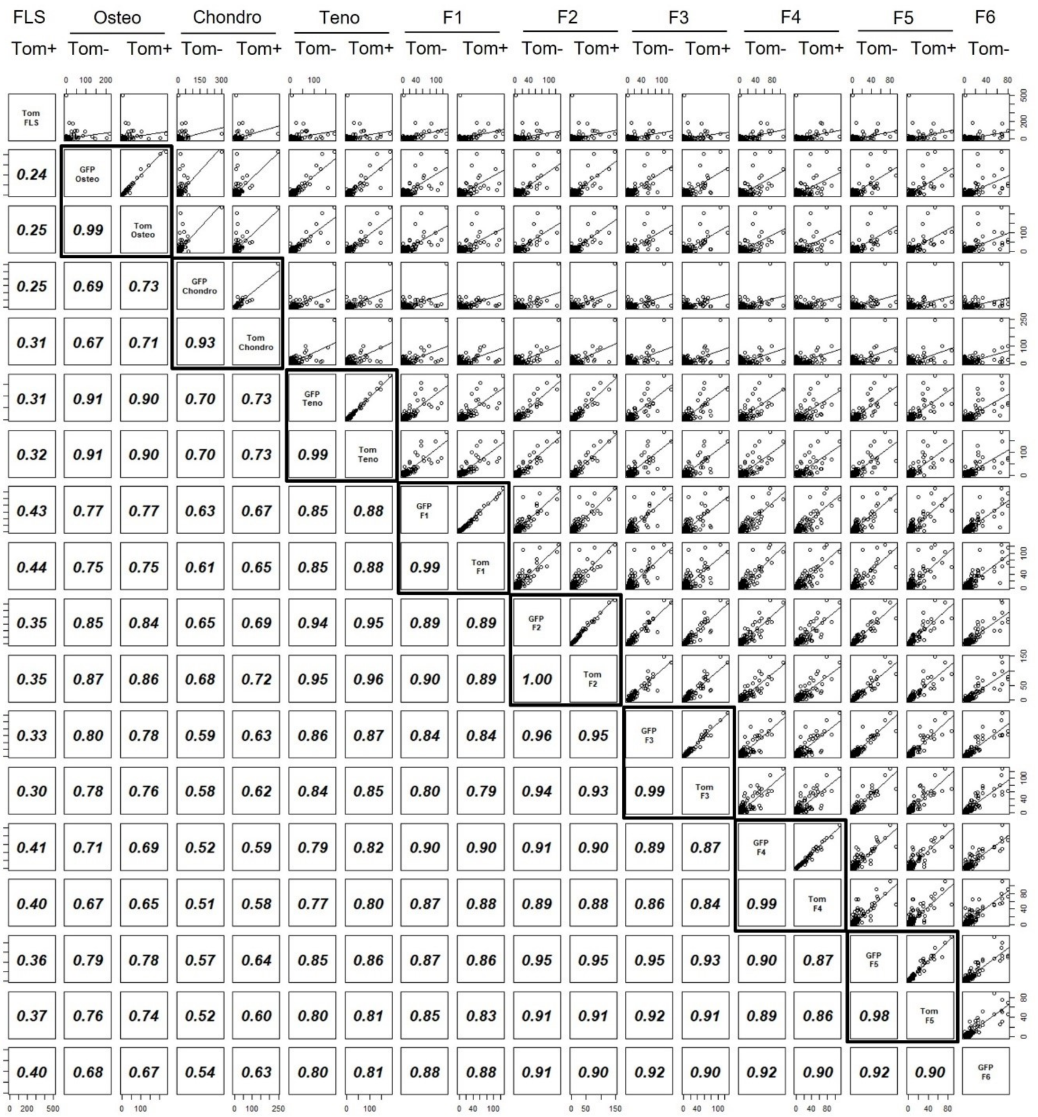

Supplementary Figure 4. Data relating to Figure 1. Pseudo-bulk transcriptome comparisons between Tom+ and Tom-GFP+ cells within the cell sub-populations identified in steady-state. Average gene expression levels were calculated for the Tom+ and Tom-GFP+ cells within each identified cell cluster. Black outlines indicate comparisons between Tom+ and Tom-GFP+ cells within each cluster. FLS: Fibroblast-like synoviocytes; Osteo: Osteoblast-lineage cells; Chondro: Chondrocyte-lineage cells; Teno: Tenocyte-lineage cells; F: Fibroblasts.

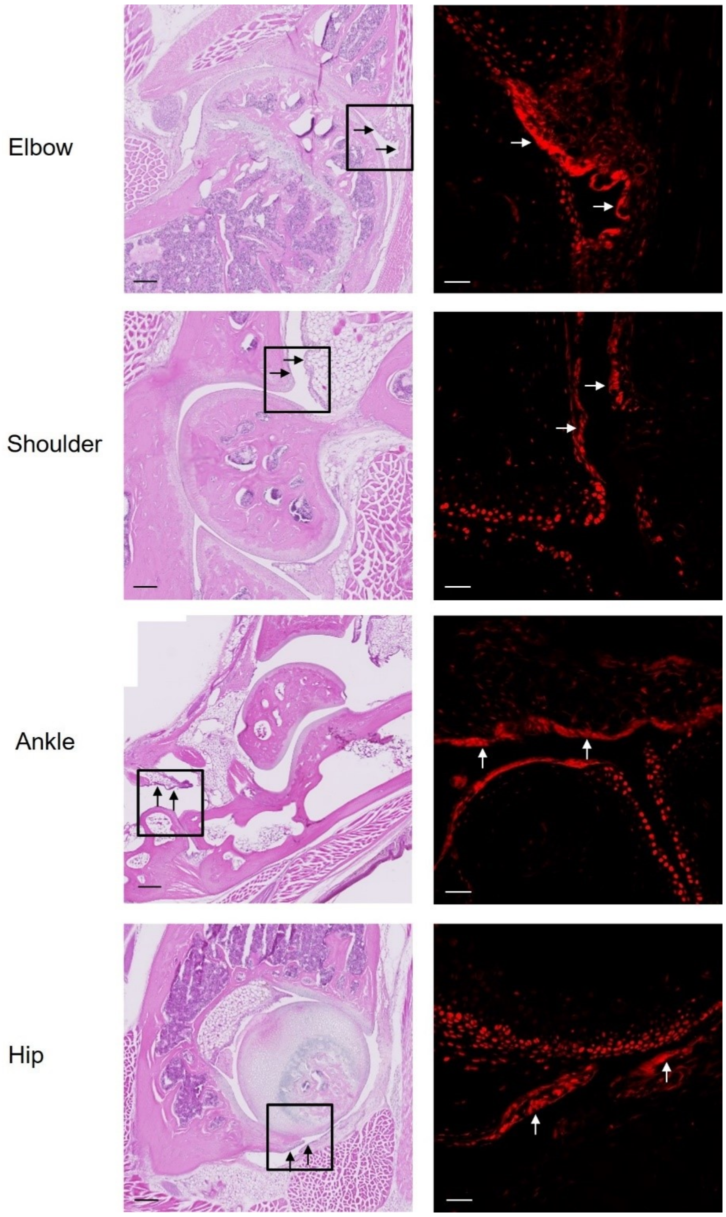

Supplementary Figure 5. Data relating to Figure 2A. Detection of Tom+ cells in synovial lining (arrows) of elbow, shoulder, ankle and hip from 15-week-old *Gdf5-Cre*;*Tom;Pdgfrα-H2BGFP* mice by histology (n=3). Left: H&E-stained sections. Scale bars: 200 µm. Right: Confocal fluorescence microscopy images of near-consecutive sections showing Tom fluorescence in red. Scale bars: 50 µm. Boxed areas in H&E images indicate approximate region shown on the right.

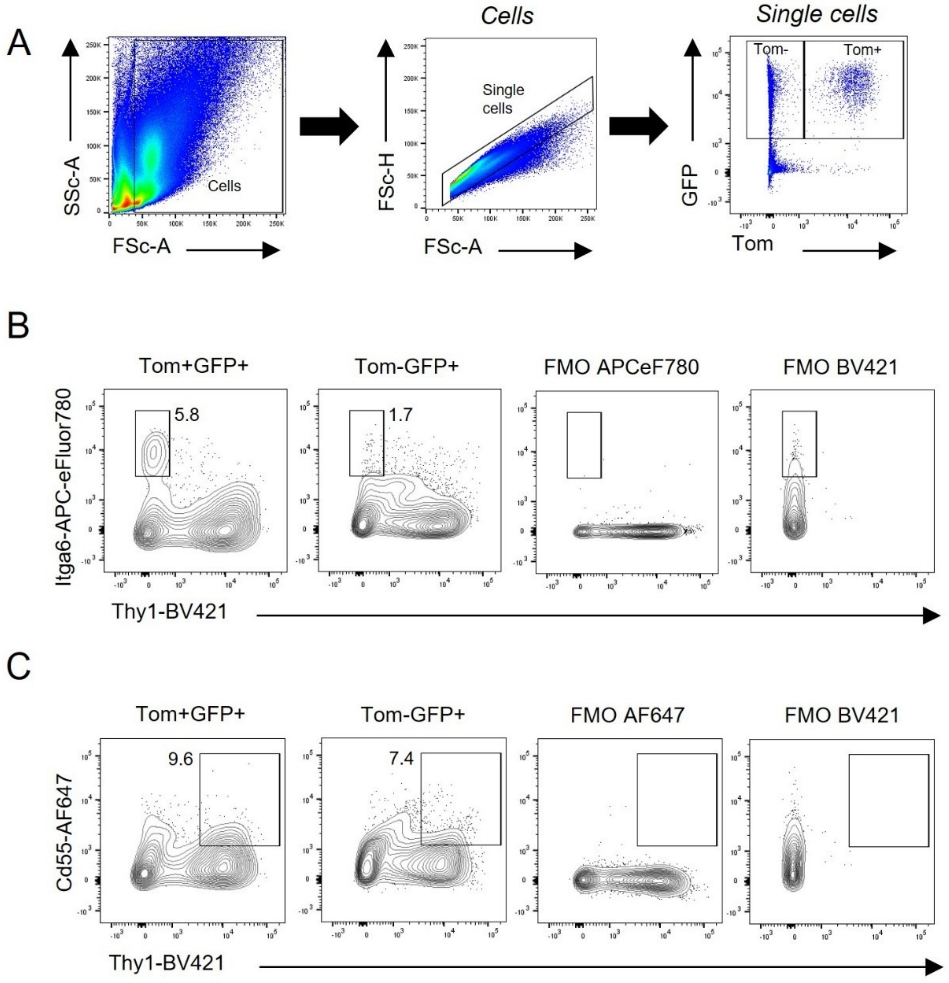

Supplementary Figure 6. Extended data Figure 2A. Cells were isolated from knees, elbows, ankles and hips of 21-to-23-week-old *Gdf5-Cre*;*Tom;Pdgfrα-H2BGFP* mice (n=8) and analysed by flow cytometry. (**A**) Gating strategy to identify Tom+GFP+ and Tom-GFP+ cells. Erythrocytes and debris were excluded based on Forward and Side Scatter profile. Doublets and aggregates were excluded based on Forward Scatter parameters. Tom+GFP+ and Tom-GFP+ cells were identified based on fluorescence. (**B,C**) Representative flow cytometry plots showing gating that was used to identify Thy1-Itga6+ FLS (**B**) and Thy1+Cd55+ fibroblasts (**C**) within the Tom+GFP+ and Tom-GFP+ cell populations. Gates were verified using fluorescence-minus-one (FMO) controls.

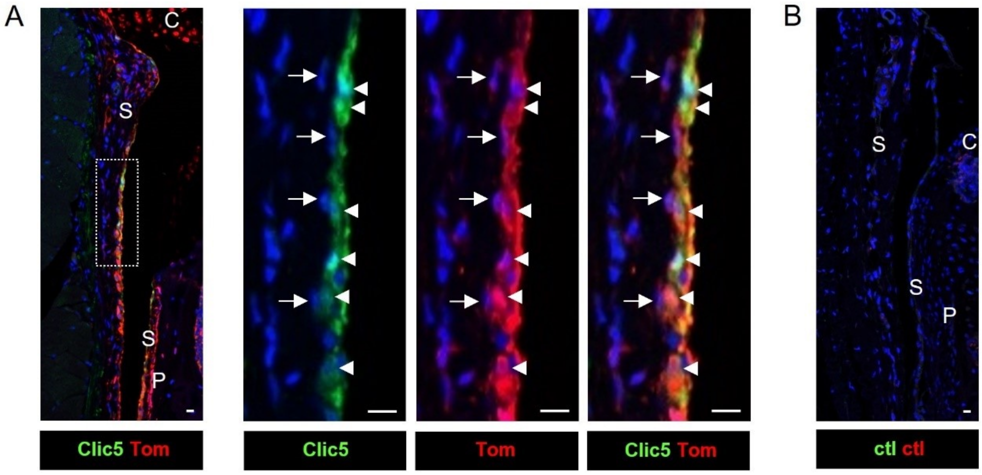

Supplementary Figure 7. Extended data Figure 2B. (**A**) Tom+ cells expressing Clic5 (arrowheads) and adjacent Tom+Clic5- cells (arrows) in synovial lining of 10-week-old *Gdf5-Cre*;*Tom;Pdgfrα-H2BGFP* mice detected by immunofluorescence staining (n=3). Note absence of Clic5 staining in cartilage. Boxed area on the left is shown enlarged on the right as separate and merged channel images. (**B**) Section stained with isotype negative control antibodies (ctl). Scale bars: 10 µm. Blue: DAPI nuclear counterstain. S: Synovium; P: Periosteum; C: Cartilage.

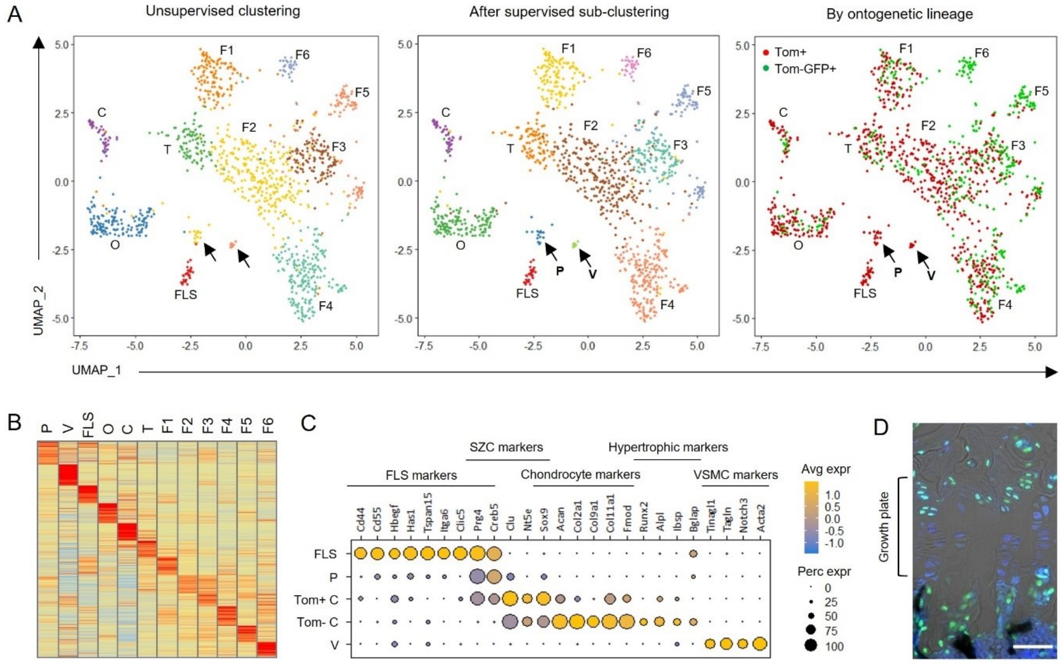

Supplementary Figure 8. Extended data Figure 2D. (**A**) UMAPs of the steady-state scRNA-seq data (see Fig. 1 for details). Left: Unsupervised clustering. Arrows indicate two small cell populations not identified as separate clusters by the unsupervised clustering algorithm. Middle: After supervised sub-clustering of the two clusters indicated by arrows. Right: Coloured by analysed cell populations showing that the two sub-clusters consisted of Tom+ cells. (**B**) Heatmap showing average expression of top 50 DEGs for all clusters identified after supervised sub-clustering. (**C**) Heatmap showing expression of selected marker genes. Cells within the P sub-cluster expressed *Prg4* and *Creb5,* but were largely negative for other FLS, superficial zone chondrocyte (SZC), and chondrocyte markers. Tom+ cells within the chondrocyte-lineage cluster expressed the superficial zone chondrocyte (SZC) markers *Prg4,*[22] *Creb5,*[23] *Clusterin,*[24] and *Nt5e,*[25] the chondrocyte-lineage transcription factor *Sox9*, and limited expression of mature chondrocyte genes, indicative of a SZC phenotype. Of note, the short collagenase digestion protocol that was used is insufficient to release chondrocytes from the deeper zones of articular cartilage, as previously shown.[4] The small number of Tom-GFP+ cells in the chondrocyte-lineage cluster did not express *Prg4* or *Creb5* and showed a mature / prehypertrophic chondrocyte phenotype. These cells likely derived from the growth plate, which was used as an incision point during knee joint dissection. The second sub-cluster was identified as vascular smooth muscle cells (V). (**D**) Histology of the tibial growth plate of an adult *Gdf5-Cre*;*Tom;Pdgfrα-H2BGFP* mouse, showing GFP expression, but not Tom expression, by chondrocytes in the upper part (proliferative/pre-hypertrophic) zone of the growth plate. Scale bar indicates 50 μm. FLS: Fibroblast-like synoviocytes; O: Osteoblast-lineage cells; C: Chondrocyte-lineage cells; T: Tenocyte-lineage cells; F: Fibroblasts.

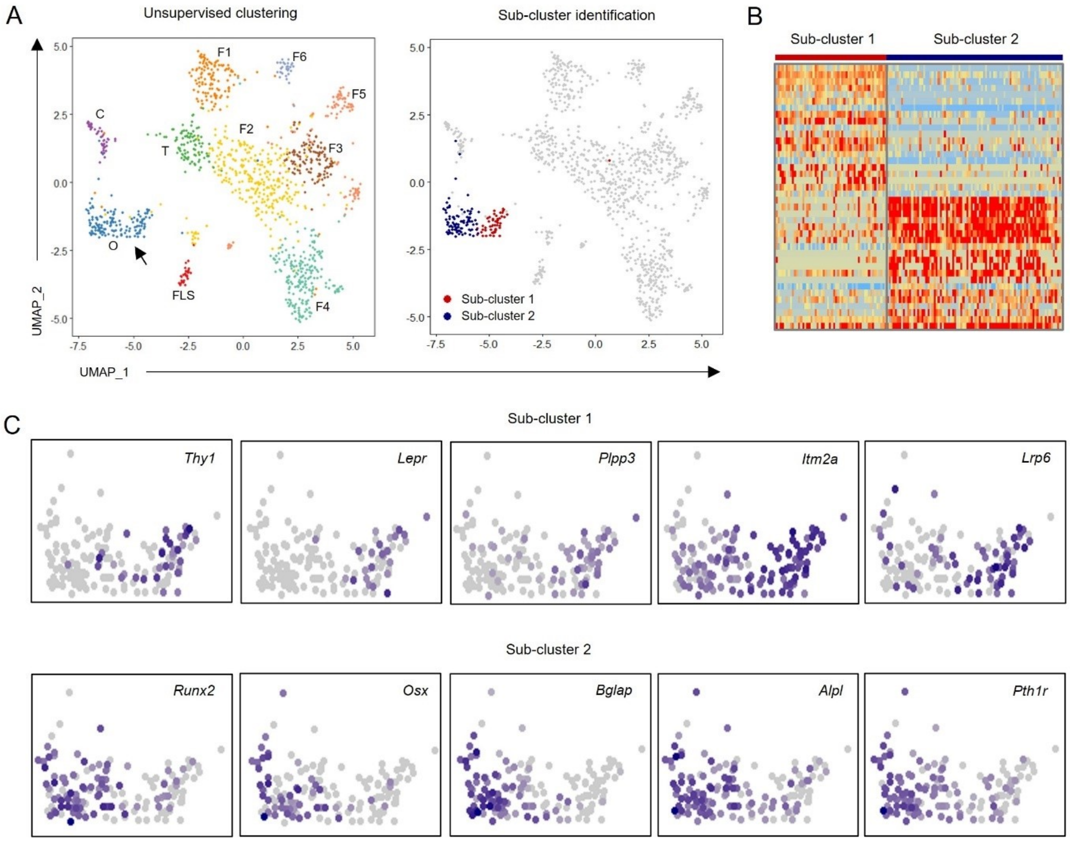

Supplementary Figure 9. Data relating to Figure 2. Osteoblast-lineage sub-cluster analysis. (**A**) UMAPs of the steady-state scRNA-seq data. Left: Unsupervised clustering. Arrow indicates osteoblast-lineage (O) cluster. Right: Osteoblast-lineage cluster was subset and re-clustered to identify sub-clusters. Two sub-clusters were identified and barcodes of cells that composed each sub-cluster were obtained and are shown in red and blue on the UMAP. (**B**) Heatmap showing expression of top 20 DEGs for the two sub-clusters. (**C**) UMAP plots showing expression of selected DEGs that were cluster-specific for the two sub-clusters, putatively identifying sub-cluster 1 as osteochondral progenitors and sub-cluster 2 as osteoblasts. FLS: Fibroblast-like synoviocytes; O: Osteoblast-lineage cells; C: Chondrocyte-lineage cells; T: Tenocyte-lineage cells; F: Fibroblasts.

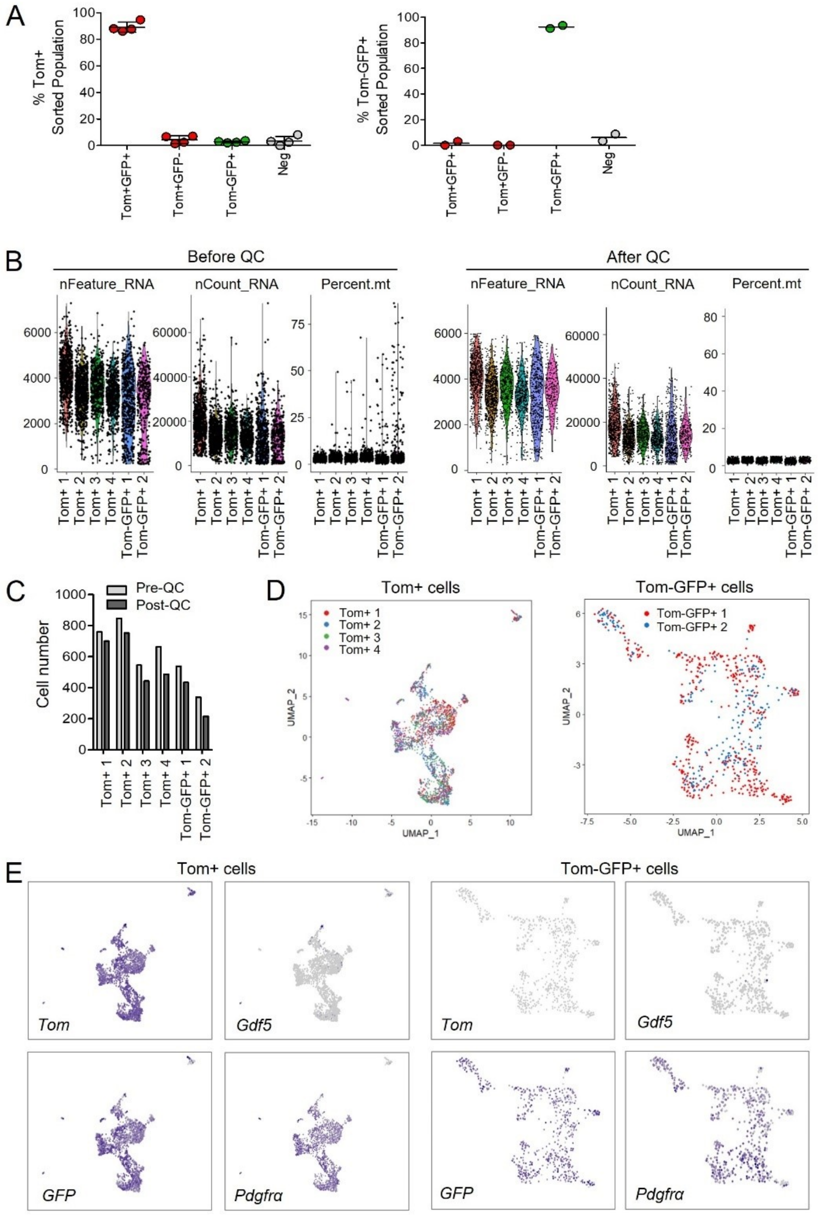

Supplementary Figure 10. Extended data Figure 3B. ScRNA-seq analysis of cells isolated from knee joints of adult *Gdf5-Cre*;*Tom*;*Pdgfrα-H2BGFP* mice after joint surface injury. scRNA-seq data of Tom+ (n=4 mice) and donor-matched Tom-GFP+ (n=2 mice) sorted cells was obtained using the 10x Genomics Chromium system. (**A**) Following sorting by FACS, aliquots of Tom+ and Tom-GFP+ collected cells were analysed on a BD Fortessa flow cytometer to confirm cell purity. Lines and error bars indicate mean ± SD. (**B**) Features, counts and percent mitochondrial genes before and after QC processing. (**C**) Number of cells present in each sample before and after QC processing of the scRNA-seq data. (**D**) Unsupervised uMAP of the Tom+ cells and Tom-GFP+ cells coloured by biological replicate (mouse). (**E**) Detection of *Tom, Gdf5, GFP* and *Pdgfrα* expression in the analysed cell populations.

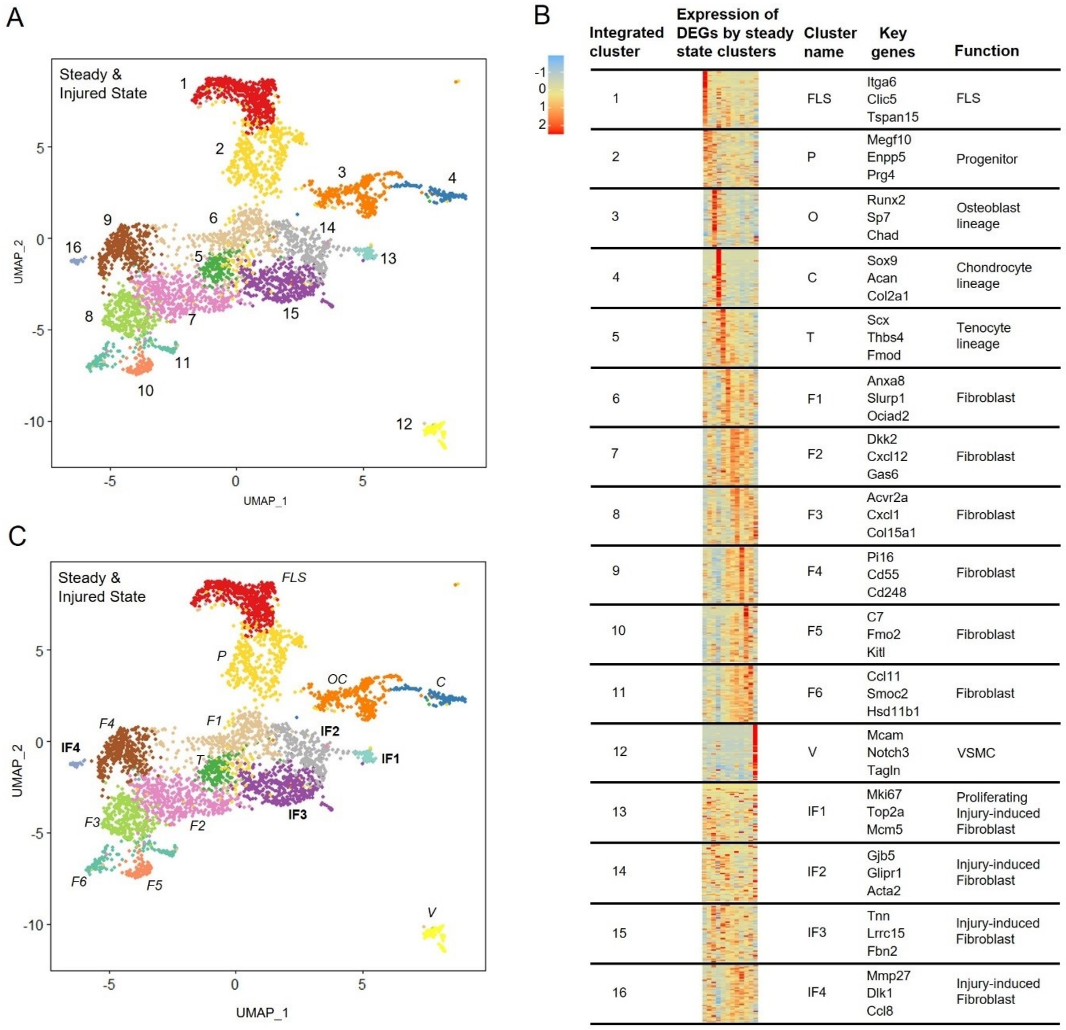

Supplementary Figure 11. Extended data Figure 3B,C. (**A**) UMAP plot of integrated scRNA-seq data from steady-state and injured-state cells showing unsupervised clustering identifying 16 cell clusters. (**B**) Analysis of differentially expressed genes (DEGs) (see Suppl. Table 6) used to identify clusters equivalent to the clusters identified in steady-state (see Fig. 1). Heatmaps show expression of the top 50 DEGs from each integrated cluster by the 10 steady-state clusters identified by unsupervised analysis (Fig. 1C) and the two steady-state clusters identified by supervised sub-clustering (Fig. 2D and Suppl. Fig. 8). Heatmap order: FLS, *Prg4*+ progenitors, osteoblast-lineage, chondrocyte-lineage, tenocyte-lineage, fibroblast clusters 1-6 and vascular smooth muscle cells. Key genes indicate selected DEGs that identify specialised cell types or are dominant cluster-specific genes. (**C**) UMAP with cluster annotation. Twelve clusters were annotated according to equivalent cluster identified in steady-state (see also Suppl. Fig. 12), with the osteoblast-lineage cluster identified as osteochondral (OC) lineage after injury (see also Suppl. Fig. 17). Four clusters only identified after injury were annotated as injury-induced fibroblasts (IF1-IF4). Injury-induced fibroblast (IF) clusters are in bold; clusters with steady-state analogues in italics.

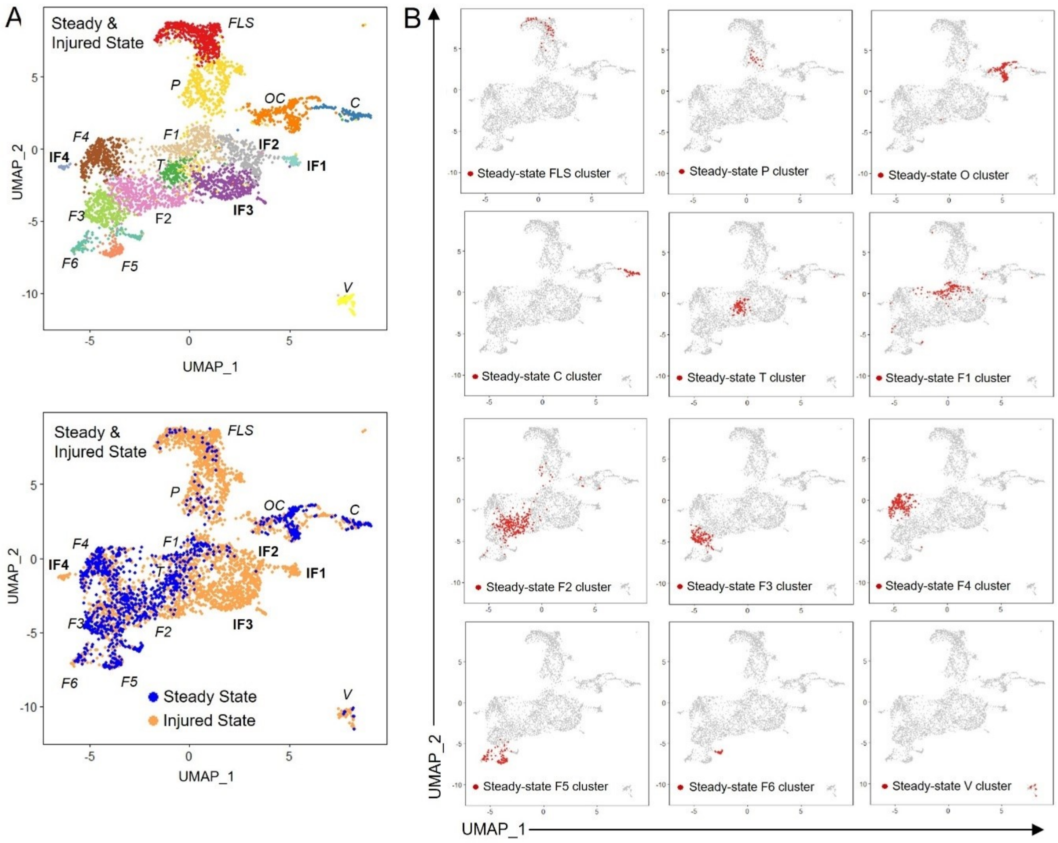

Supplementary Figure 12. Extended data Figure 3B,C. UMAP plots of integrated scRNA-seq data from steady-state and injured-state cells showing (A) Top: unsupervised clustering. Bottom: Colour coded by analysed state. Injury-induced fibroblast (IF) clusters are in bold; clusters with steady-state analogues in italics. (B) The location, in red, of steady-state cells within each of the clusters identified in steady-state (see Fig. 1) to verify cluster annotation. The location of steady-state cluster cells on the integrated uMAP correlates well with the integrated uMAP clustering and annotation, with the exception of clusters F5 and F6, which clustered differently between steady-state and integrated uMAP. FLS: Fibroblast-like synoviocytes; P: *Prg4*+ progenitors; O: Osteoblast-lineage cells; C: Chondrocyte-lineage cells; T: Tenocyte-lineage cells; F: Fibroblasts; V: Vascular smooth muscle cells.

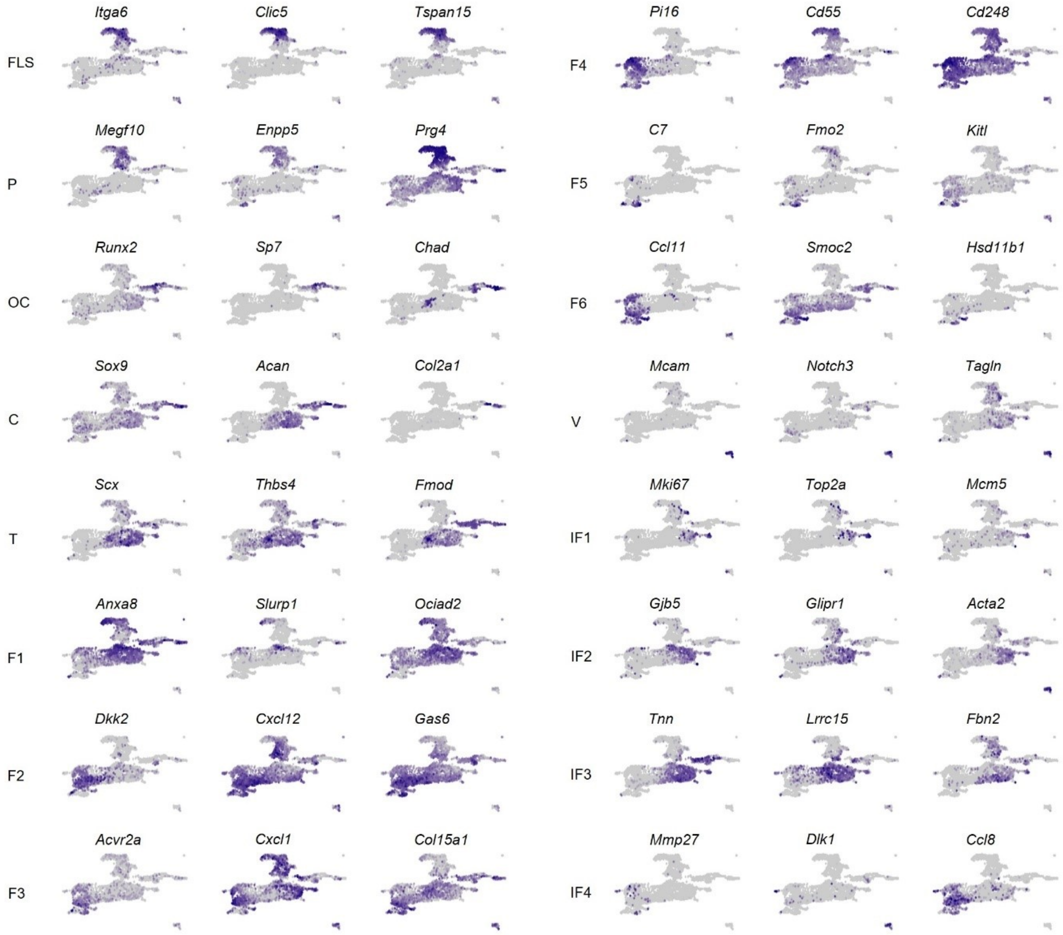

Supplementary Figure 13. Extended data Fig. 3B,C. UMAP plots showing the expression of selected DEGs for each cluster that identify specialised cell types or are dominant cluster-specific genes. FLS: Fibroblast-like synoviocytes; P: *Prg4*+ Progenitors; OC: Osteochondral-lineage cells; C: Chondrocyte-lineage cells; T: Tenocyte-lineage cells; F: Fibroblasts; V: Vascular smooth muscle cells; IF: Injury-induced fibroblasts.

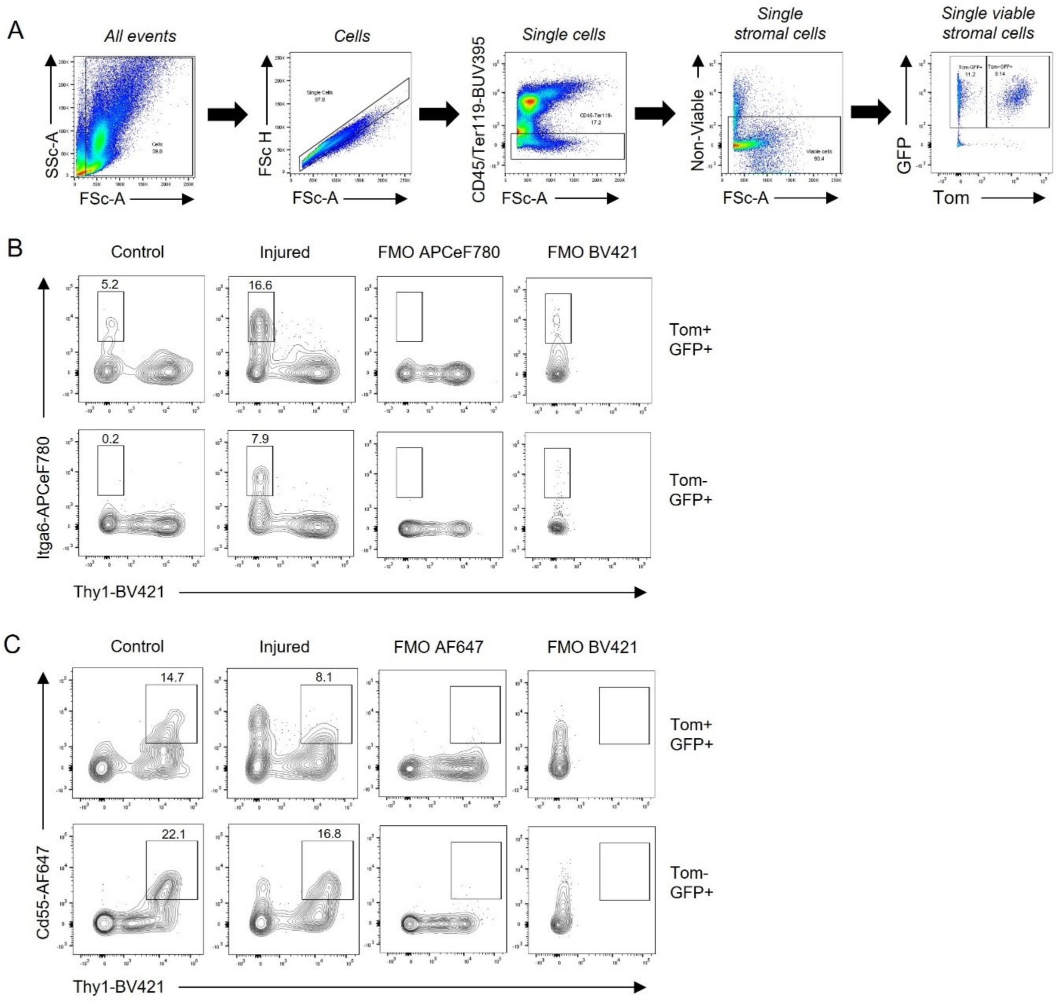

Supplementary Figure 14. Extended data Figure 3E. Freshly isolated cells from knees of 15-to-18-week-old *Gdf5-Cre*;*Tom;Pdgfrα-H2BGFP* mice 6 days after joint surface injury were analysed by flow cytometry (n=7). (**A**) Gating strategy to identify GFP+Tom+ and GFP+Tom- cells. Erythrocytes and debris were excluded based on Forward and Side Scatter profile. Doublets and aggregates were excluded based on Forward Scatter parameters. Haematopoietic cells and erythrocytes were further excluded based on CD45 and Ter119 staining. Dead cells were excluded based on viability dye staining. (**B,C**) Representative flow cytometry plots showing Itga6 and Thy1 expression (**B**) and Cd55 and Thy1 expression (**C**) within the GFP+Tom+ and GFP+Tom- cell populations. Injured: Cells isolated from injured knee; Control: Cells isolated from contralateral control knee. Gates were set using fluorescence-minus-one (FMO) controls.

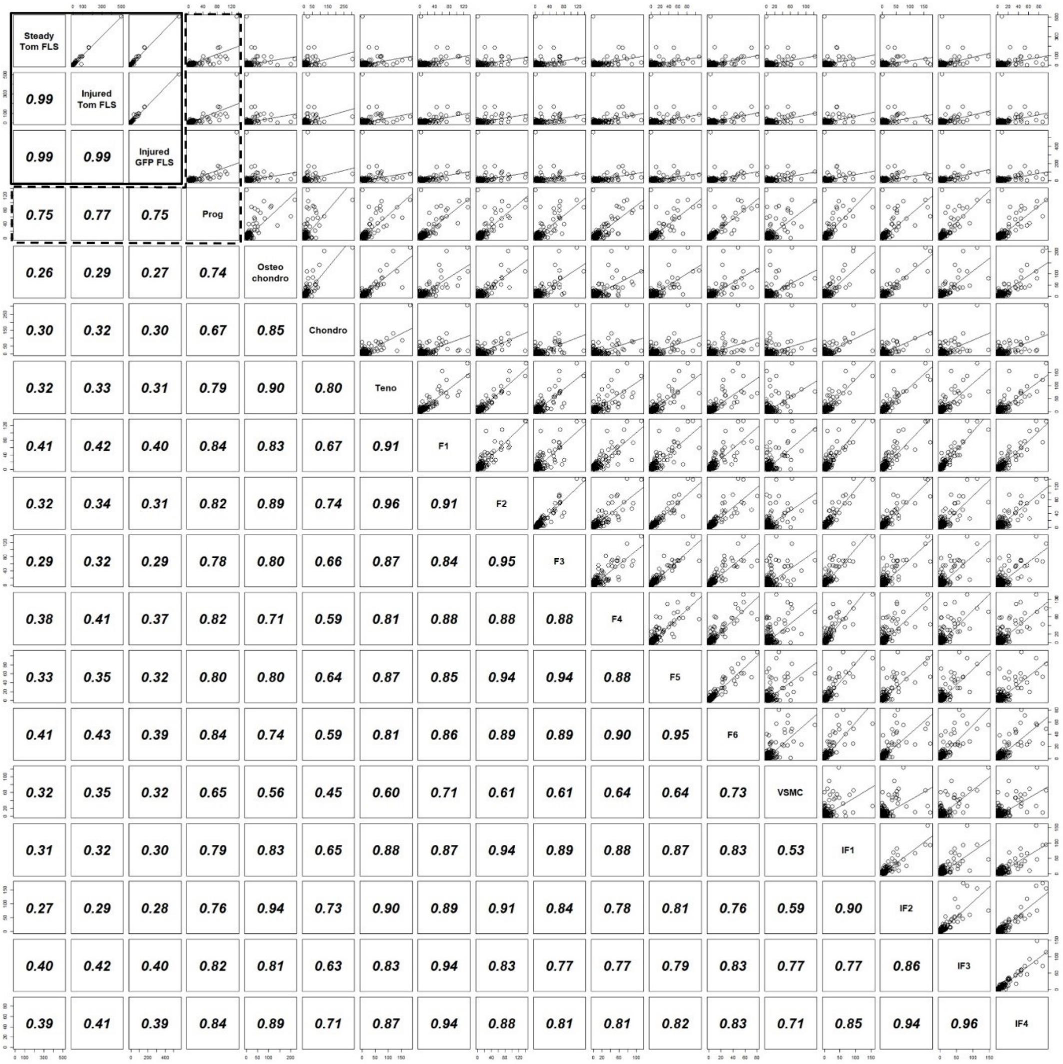

Supplementary Figure 15. Data relating to Figure 3. Pseudo-bulk transcriptome comparisons between FLS and *Prg4*+ progenitors. Average gene expression levels were calculated for the steady-state and injured-state Tom+ and Tom-GFP+ FLS and all cells within each identified integrated cell cluster. Solid black outline indicates comparisons between the steady-state and injured-state Tom+ and Tom-GFP+ FLS. Dotted black outline indicates comparison between the FLS and *Prg4*+ progenitors. FLS: Fibroblast-like synoviocytes; Prog: *Prg4*+ Progenitors; Osteo: Osteochondral-lineage cells; Chondro: Chondrocyte-lineage cells; Teno: Tenocyte-lineage cells; F: Fibroblasts; IF: Injury-induced fibroblasts; VSMC: Vascular smooth muscle cells.

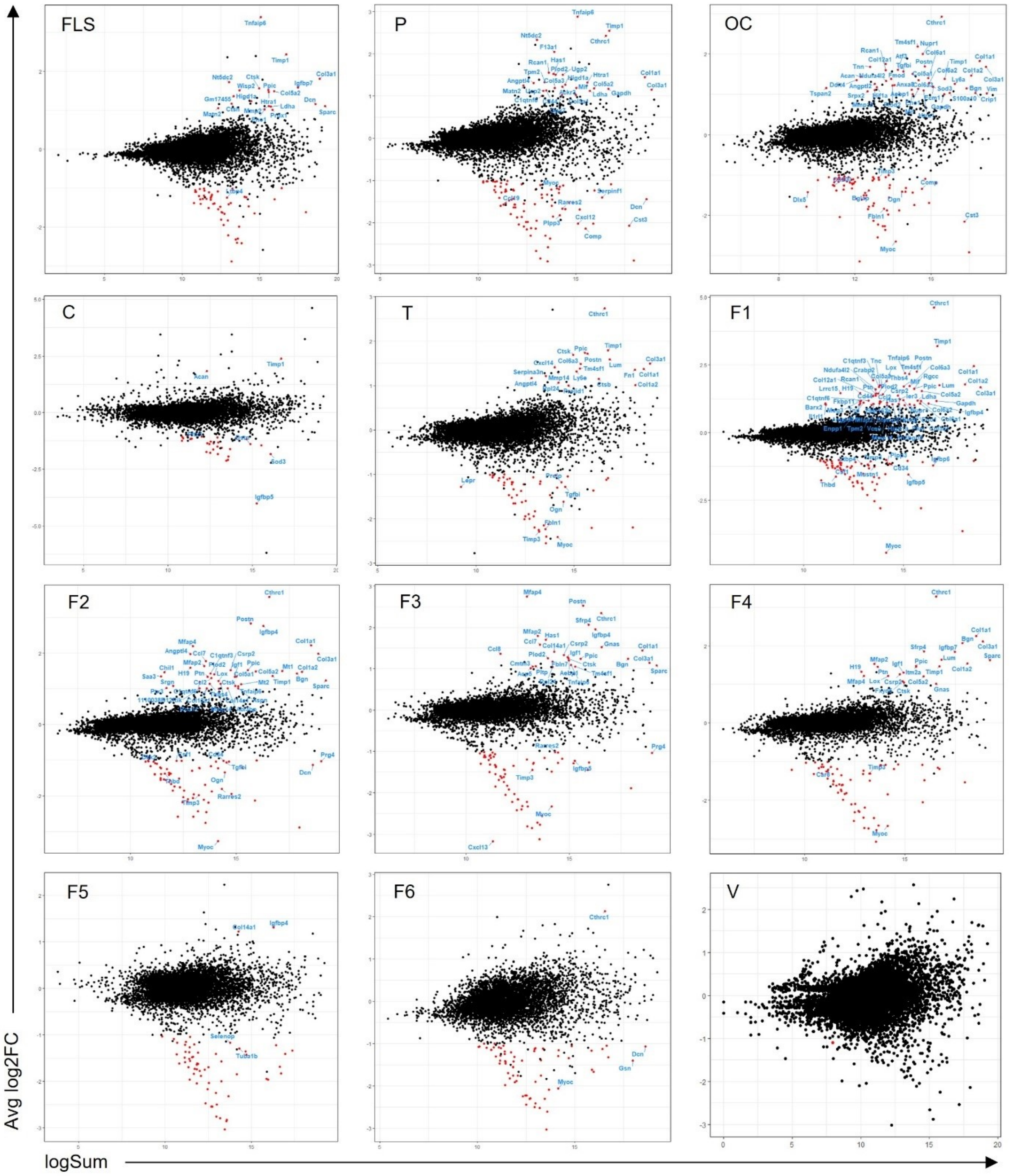

Supplementary Figure 16. Data relating to Figure 3. Transcriptomic changes between steady-state and injured-state cells within identified clusters. MA plots showing differentially expressed genes (log2FC > / < 1 and p<0.01; red points) following injury in each of the shared clusters**.** Significantly upregulated and selected downregulated genes are labelled. Majority of downregulated genes were predicted genes or pseudogenes (not shown). FLS: Fibroblast-like synoviocytes; P: *Prg4*+ Progenitors; OC: Osteochondral-lineage cells; C: Chondrocyte-lineage cells; T: Tenocyte-lineage cells; F: Fibroblasts; V: Vascular smooth muscle cells.

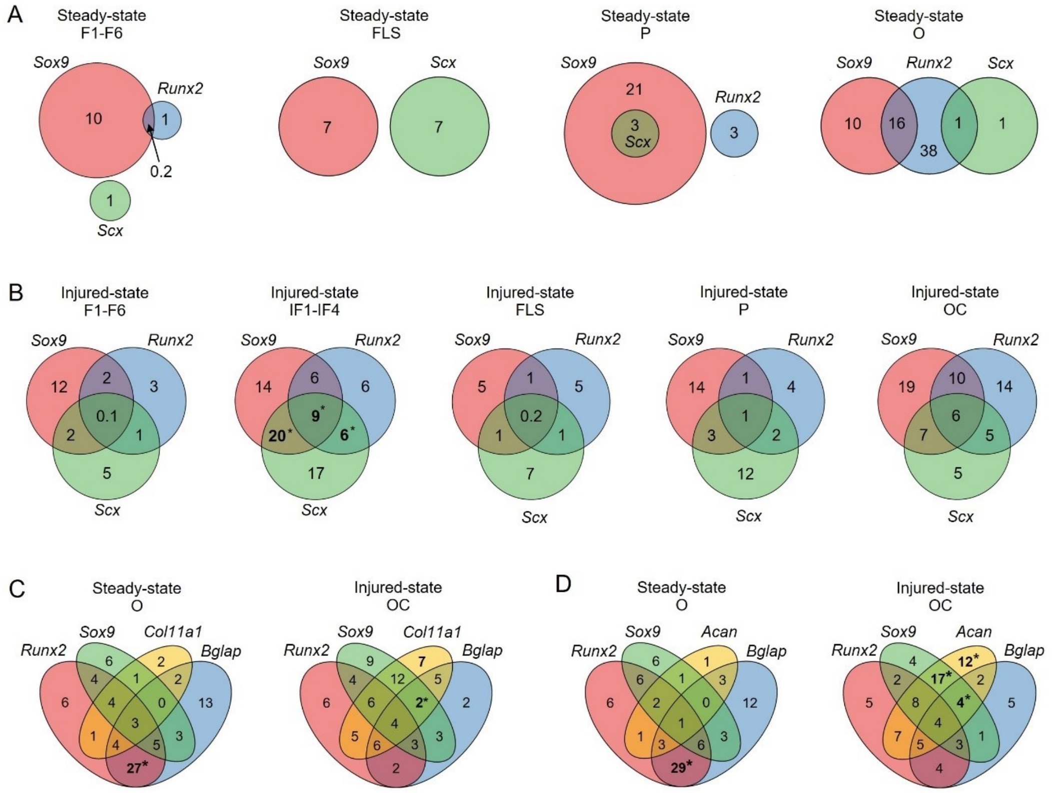

Supplementary Figure 17. Extended data Figure 3G. Venn diagrams showing the percentage of (**A**) steady-state cells and (**B**) injured-state cells within indicated clusters that express / co-express the skeletal lineage-specifying transcription factors *Sox9*, *Runx2* and *Scx*. *=FDR <0.05 between injured state F1-F6 and injured state IF1-IF4, negative binomial generalized linear model with Benjamini-Hochberg post-test. (**C,D**) Venn diagrams showing the percentage of osteochondral cells that express / co-express the genes (**C**) *Runx2*, *Sox9*, *Col11a1* and *Bglap* or (**D**) *Runx2*, *Sox9*, *Acan* and *Bglap* in steady-state and after injury. *=FDR <0.05 between steady-state O cluster and injured-state OC cluster, negative binomial generalized linear model with Benjamini-Hochberg post-test. FLS: Fibroblast-like synoviocytes; P: *Prg4*+ Progenitors; O: Osteoblast-lineage cells; OC: Osteochondral-lineage cells; F: Fibroblasts; IF: Injury-induced fibroblasts.

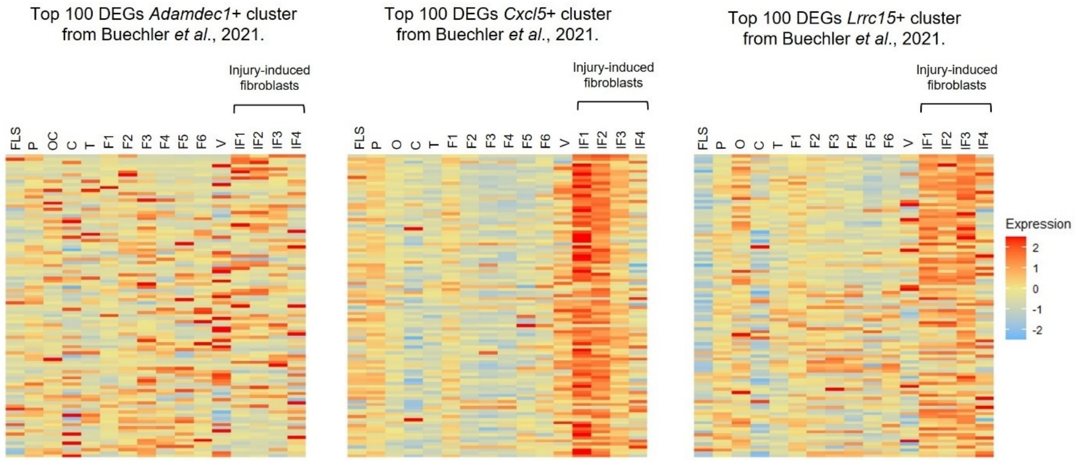

Supplementary Figure 18. Comparison of injury-induced fibroblast phenotype to previously identified perturbed fibroblast populations. Heatmaps showing expression of the top 100 DEGs of the *Adamdec1*+, *Cxcl5*+ and *Lrrc15*+ perturbed fibroblast populations identified by Buechler *et al*.[26] in the steady-state and injured-state integrated dataset. FLS: Fibroblast-like synoviocytes; P: *Prg4*+ Progenitors; OC; Osteochondral-lineage cells; C: Chondrocyte-lineage cells; T: Tenocyte-lineage cells; F: Fibroblasts; IF: Injury-induced fibroblasts; V: Vascular smooth muscle cells.

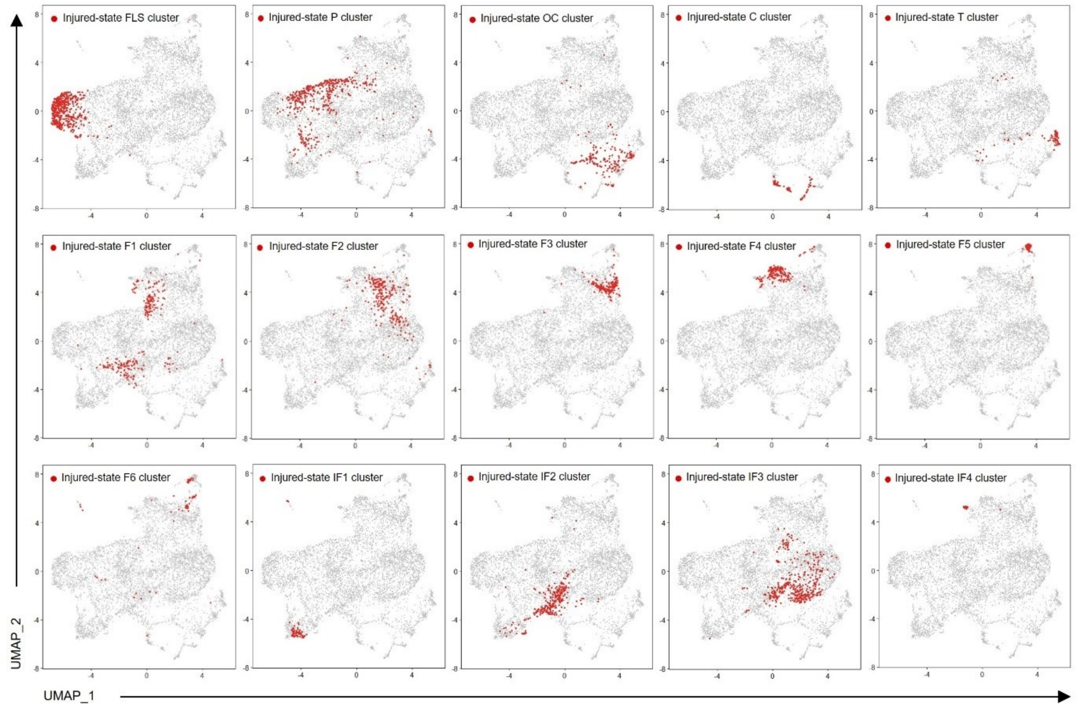

Supplementary Figure 19. Extended data Figure 4A. UMAP plots showing in red the location of injured-state clusters (Fig. 3) mapped onto the integrated injured-state and STIA dataset. FLS: Fibroblast-like synoviocytes; P: *Prg4*+ Progenitors; OC: Osteochondral-lineage cells; C: Chondrocyte-lineage cells; T: Tenocyte-lineage cells; F: Fibroblasts; IF: Injury-induced fibroblasts.

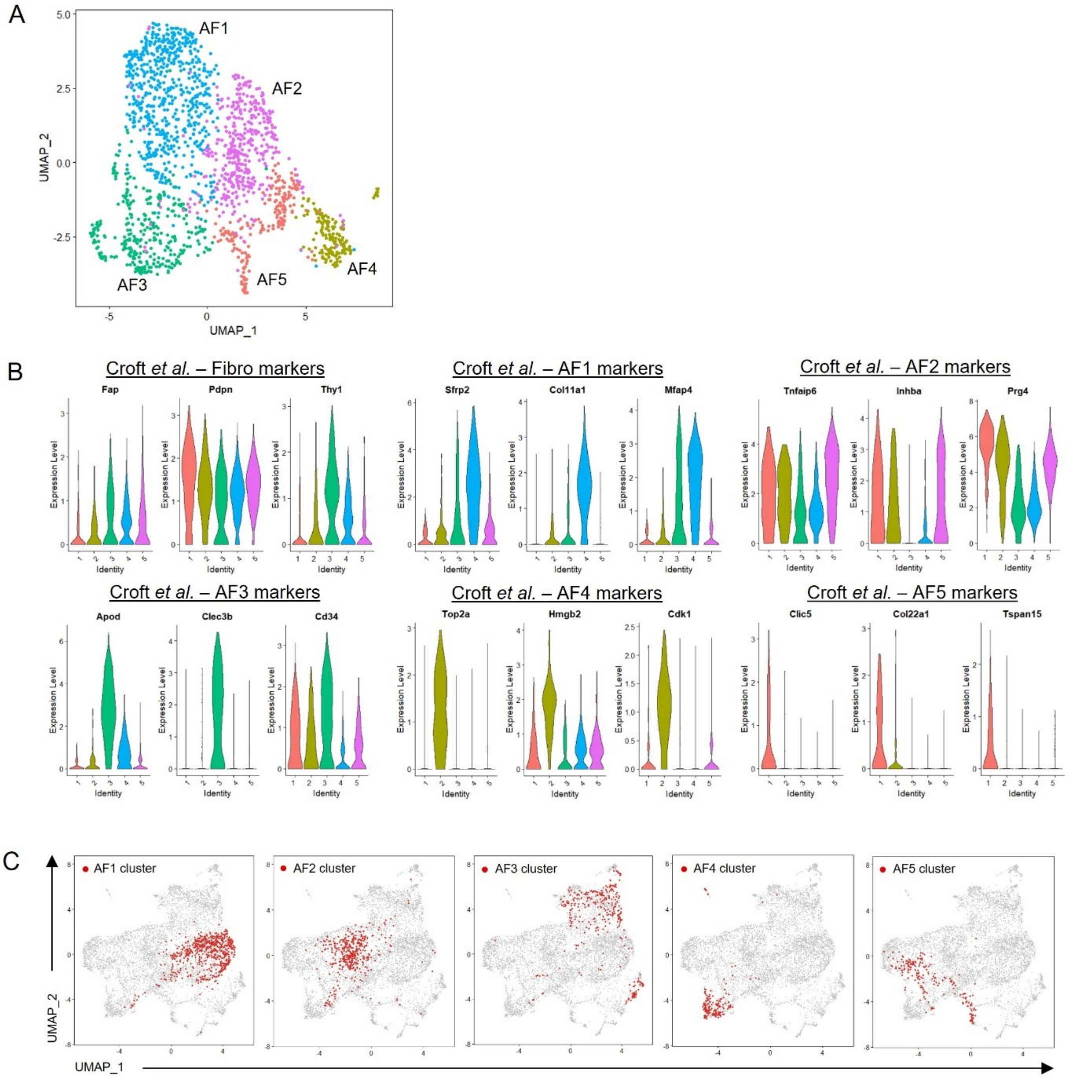

Supplementary Figure 20. Extended data Figure 4A. Identification of clusters in the STIA dataset. The serum transfer induced arthritis (STIA) scRNA-seq dataset was obtained from NCBI GEO GSE129087.[18] Data underwent QC, was down-sampled to consist of 1,725 cells and (**A**) clustered in an unbiased manner at a resolution that provided the same number of clusters as in the paper by Croft *et al*. [18]. (**B**) Clusters were annotated based upon the markers identified by Croft *et al*. [18] and (**C**) mapped, in red, onto the integrated injured-state and STIA dataset.

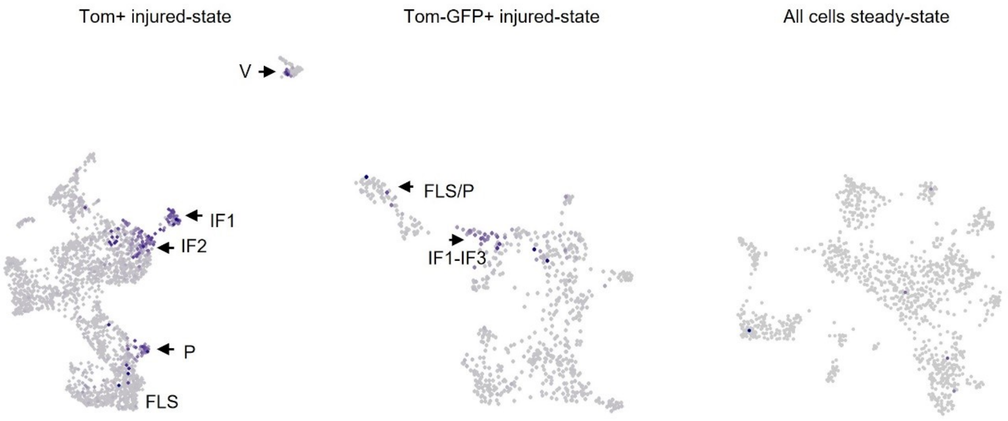

Supplementary Figure 21. Extended data Figure 5C. UMAP plots of cell cycle module score based on expression of *Mki67*, *Ccna2*, *Ccnb1*, *Ccnb2* and *Cdk1* for injured-state Tom+ cells, injured-state Tom-GFP+ cells, and steady-state Tom+ and Tom-GFP+ cells. Arrows indicate proliferating cell populations. FLS: Fibroblast-like synoviocytes; P: *Prg4*+ progenitors; IF: injury-induced fibroblasts. V: Vascular smooth muscle cells.

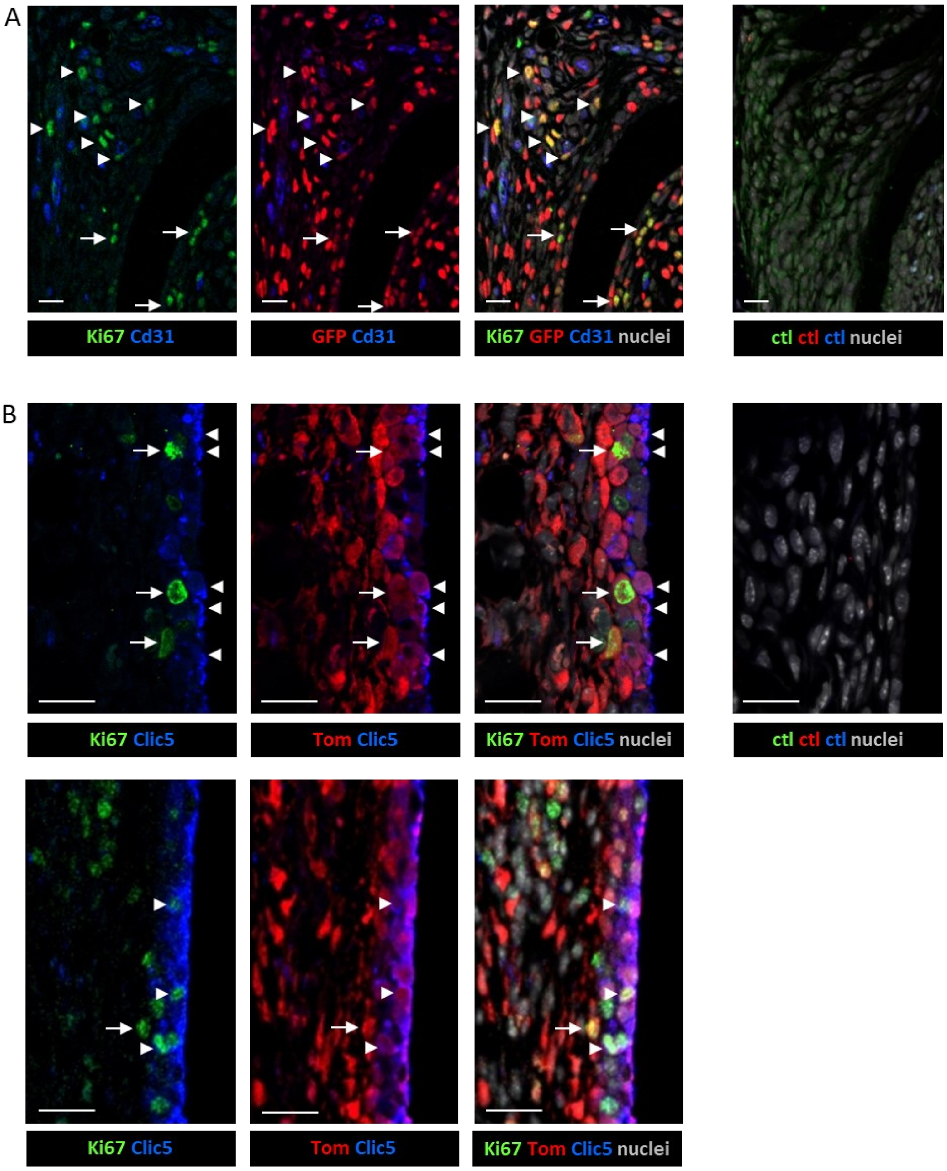

Supplementary Figure 22. Extended data Figure 5D,E. Immunofluorescence staining in *Gdf5-Cre*;*Tom;Pdgfrα-H2BGFP* mouse synovium 6 days after injury to locate proliferating fibroblasts. (**A**) Ki67+ and GFP+ proliferating fibroblasts in synovial lining (arrows), and near Cd31+ blood vessels in synovial sub-lining (arrowheads). The same microscopy image is shown from left to right with different fluorescence channels visualised as indicated. Tissue section stained with isotype negative control antibodies is shown on the right. Single-stained tissue sections served as additional controls (not shown). (**B**) Top: Ki67+Tom+Clic5- proliferating fibroblasts in synovial lining (arrows), adjacent to Clic5+Tom+ FLS (arrowheads). Bottom: Proliferating Ki67+Tom+Clic5- synovial lining fibroblast (arrow) and Ki67+Tom+Clic5+ FLS (arrowheads) in synovial lining. The same microscopy image is shown from left to right with different fluorescence channels visualised as indicated. Tissue section stained with isotype negative control antibodies is shown on the right. Single-stained tissue sections served as additional controls (not shown). All scale bars: 20 µm.

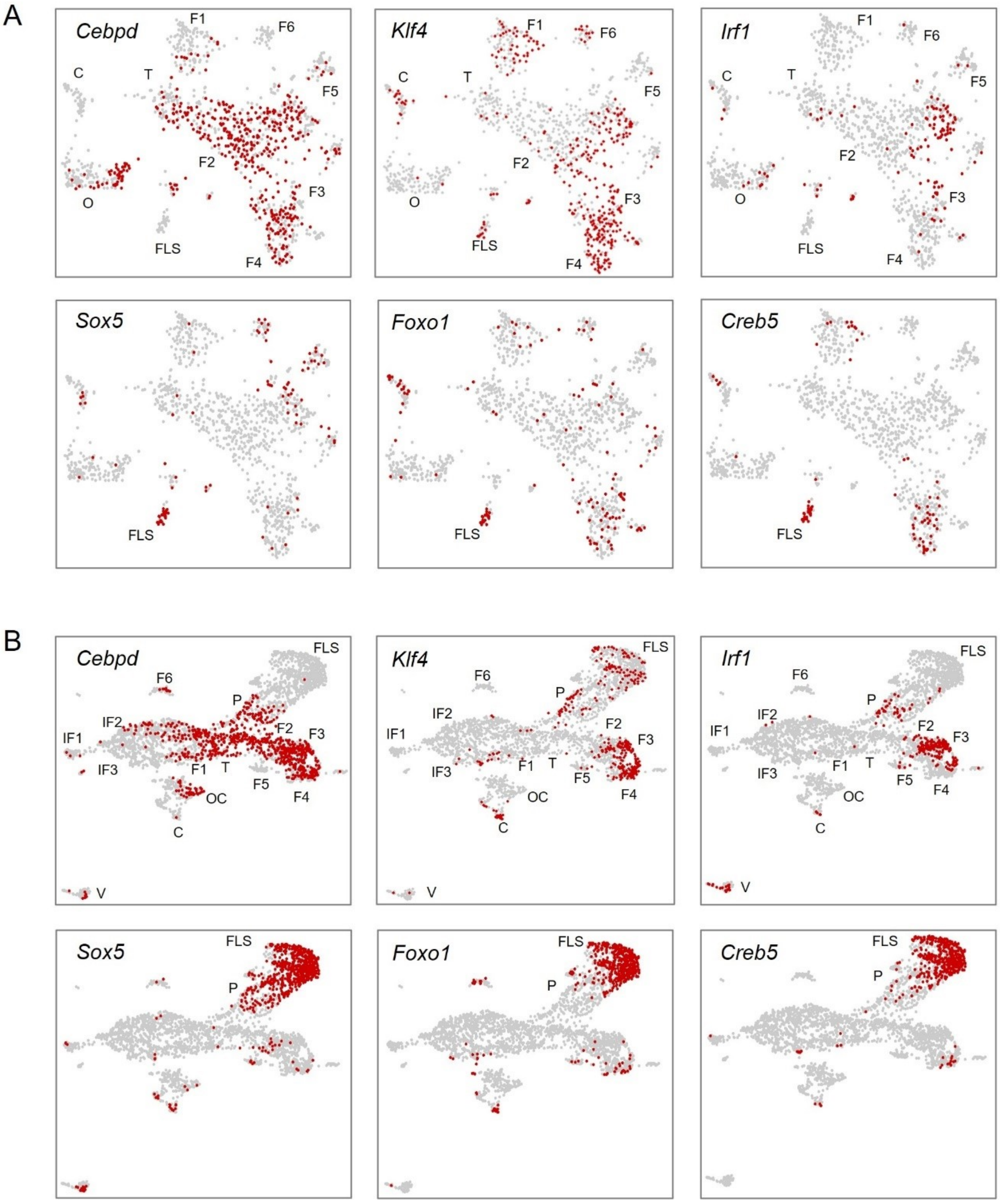

Supplementary Figure 23. Extended data Figure 6B. Analysis of regulon activity associated with *Thy1*+ fibroblast and FLS in separated (**A**) steady-state and (**B**) injured-state datasets. FLS: Fibroblast-like synoviocytes; P: *Prg4*+ progenitors; O: Osteoblast-lineage cells; OC: Osteochondral-lineage cells; C: Chondrocyte-lineage cells; T: Tenocyte-lineage cells; F: Fibroblasts; IF: Injury-induced fibroblasts; V: Vascular smooth muscle cells.

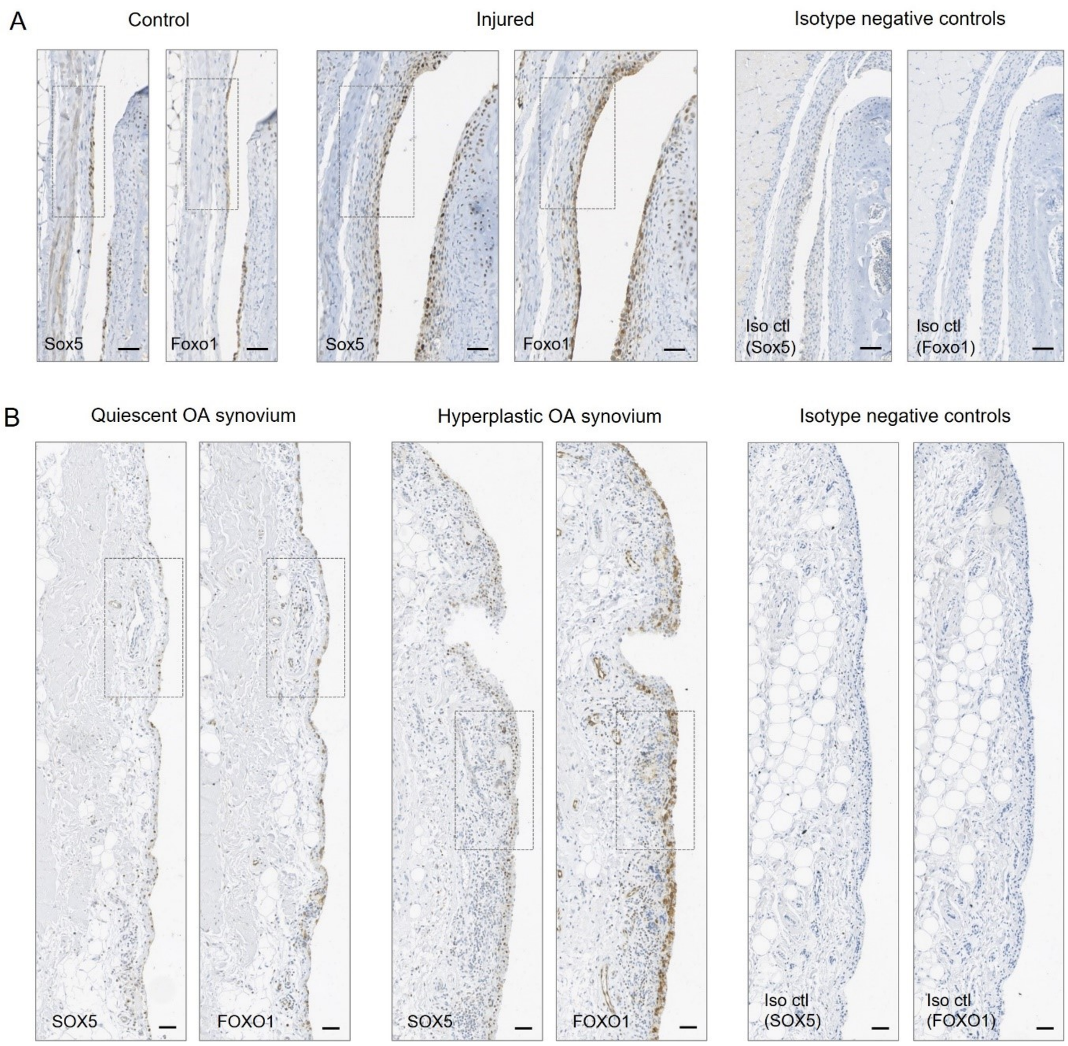

Supplementary Figure 24. Extended data Figure 6D,G. Detection of Sox5 and Foxo1 in mouse and human synovium. (**A**) Immunohistochemical detection of Sox5 and Foxo1 in synovium in near-consecutive tissue sections of 11-to-13-week-old mice 7 days post-injury (n=5) showing expression in synovial lining in control (unoperated contralateral control knee) and injured knees. Boxed areas indicate region shown in Fig. 6D. Iso ctl: sections stained with isotype negative control antibody. Scale bars: 50 µm. (**B**) Immunohistochemical detection of SOX5 and FOXO1 in near-consecutive tissue sections of human OA synovium obtained at arthroplasty (n=6), showing expression in quiescent and hyperplastic areas of synovial lining. Boxed areas indicate region shown in Fig. 6G. Scale bars: 20 µm.

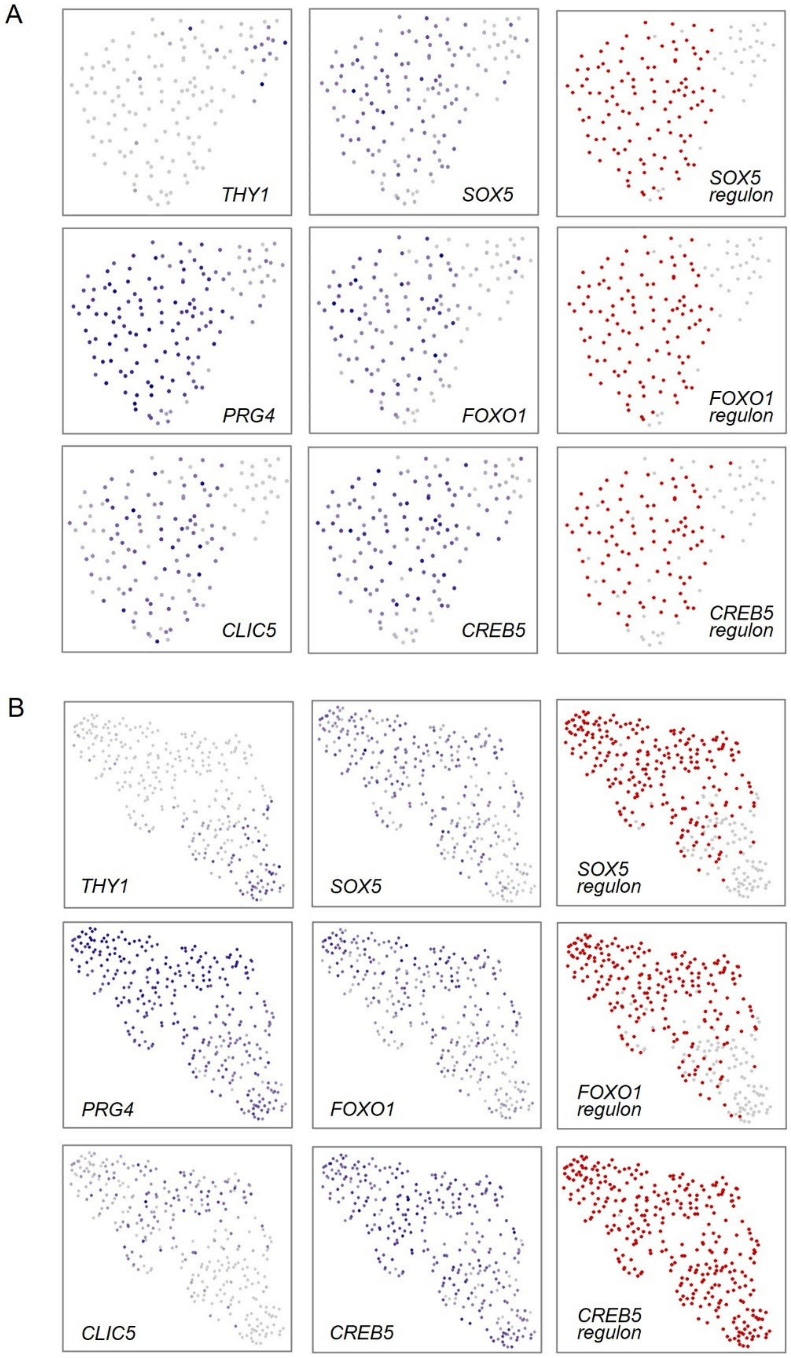

Supplementary Figure 25. Extended data Figure 6F. Identification of FLS regulon activity in additional human OA synovial cell datasets. UMAP plots showing expression of marker genes, transcription factors and FLS regulon activity in human OA scRNA-seq datasets from (**A**) Mizoguchi *et al*. (n=2 patients)[20] and (**B**) Zhang *et al*. (n = 3 patients).[21]

Supplementary Table 1. OA patient information

Patient	Age	Sex	Joint
1	65	M	Knee
2	81	M	Knee
3	65	F	Knee
4	79	F	Knee
5	68	F	Knee
6	65	F	Knee

Supplementary Table 2. Antibodies for immunohistochemistry and immunofluorescence staining

Antibody	Clone	Manufacturer	Cat. No.	Conjugation
mCherry (Tom)	Polyclonal	Sicgen Antibodies	AB0081-200	Unconjugated
GFP	Polyclonal	Abcam	ab13970	Unconjugated
Sox5	Polyclonal	Abcam	Ab94396	Unconjugated
Clic5	Polyclonal	Novus Biologicals (Bio-techne)	NBP1-80075	Unconjugated
Sox9	EPR14335-78	Abcam	ab185966	Unconjugated
Runx2	EPR14334	Abcam	ab192256	Unconjugated
Foxo1	C29H4	Cell Signalling	2880	Unconjuagted
Cd31	EPR17259	Abcam	ab182981	Unconjugated
Ki-67	Monoclonal	eBioscience	14-5698-82	Unconjugated
Rabbit IgG isotype control	Polyclonal	Abcam	ab37415	Unconjugated
Normal Rabbit IgG control	Polyclonal	R&D Systems (Bio-techne)	AB-105-C	Unconjugated
Normal Chicken IgY control	Polyclonal	R&D Systems (Bio-techne)	AB-101-C	Unconjugated
Normal Goat IgG control	Polyclonal	R&D Systems (Bio-techne)	AB-108-C	Unconjugated
Rabbit IgG XP® isotype control	DA1E	Cell Signaling Technology	3900	Unconjugated
Rat IgG1 isotype control	43414	R&D Systems (Bio-techne)	MAB005	Unconjugated
Goat anti-rabbit IgG (H&L)	Polyclonal	Vector Laboratories	BA-1000	Biotinylated
Horse anti-rabbit IgG (H&L)	Polyclonal	Vector Laboratories	BA-1100	Biotinylated
Streptavidin	1C2	Novus Biologicals (Bio-techne)	NBP1-04345B	Biotinylated
Donkey anti-rabbit IgG (H&L)	Polyclonal	Abcam	ab150065	Alexa Fluor®488
Donkey anti-goat IgG (H&L)	Polyclonal	Abcam	ab15029	Alexa Fluor®488
Goat anti-chicken IgY (H&L)	Polyclonal	Abcam	ab150173	Alexa Fluor®488
Donkey anti-goat IgG (H&L)	Polyclonal	Abcam	ab150136	Alexa Fluor®594
Donkey anti-rat IgG (H&L)	Polyclonal	Abcam	ab150156	Alexa Fluor®594
Donkey anti-rabbit IgG (H&L)	Polyclonal	Abcam	ab150067	Alexa Fluor®647

Supplementary Table 3. Antibodies for flow cytometry

Antibody	Clone	Manufacturer	Cat. No.	Conjugation
Cd55	RIKO-3	Biolegend	131806	Alexa Fluor®647
Cd49f (Itga6)	eBioGoH3	eBioscience	47-0495-82	APCef780
Cd90 (Thy1)	OX-7	BD Biosciences	563770	BV421

Supplementary Table 4. Samples analysed by scRNA-seq in this study.

Sample	Mouse	Cells	Condition	Cell number pre-QC	Cell number post-QC
1	1	Tom+	Steady state	333	297
2	2	Tom+	Steady state	547	489
3	2	Tom-GFP+	Steady state	394	376
4	3	Tom+	Injury	760	700
5	4	Tom+	Injury	848	752
6	5	Tom+	Injury	546	443
7	5	Tom-GFP+	Injury	539	434
8	6	Tom+	Injury	664	488
9	6	Tom-GFP+	Injury	339	217

Supplementary Table 5. Top 10 DEGs for each cluster in steady state.

Cluster	Gene	p_val	avg_log2FC	pct.1	pct.2	p_val_adj
FLS	F13a1	5.74E-171	2.888714	0.906	0.01	1.10E-166
FLS	Col22a1	3.79E-166	2.335707	0.75	0.003	7.26E-162
FLS	Clic5	3.34E-136	3.467433	0.875	0.016	6.39E-132
FLS	Gchfr	6.46E-104	1.111849	0.562	0.006	1.24E-99
FLS	Tspan15	4.99E-103	2.685473	0.875	0.03	9.56E-99
FLS	Tmem196	1.64E-102	0.800652	0.5	0.003	3.14E-98
FLS	Dlx3	9.02E-86	1.181168	0.781	0.028	1.73E-81
FLS	Itga6	2.17E-73	1.630272	0.781	0.038	4.15E-69
FLS	Fut9	6.10E-67	0.360444	0.312	0.002	1.17E-62
FLS	Rab37	1.91E-65	0.504096	0.281	0.001	3.66E-61

Cluster	Gene	p_val	avg_log2FC	pct.1	pct.2	p_val_adj
Osteo	Ibsp	3.31E-149	4.065684	0.773	0.028	6.33E-145
Osteo	Cdh11	2.08E-118	1.969991	0.773	0.062	3.98E-114
Osteo	Bglap	4.31E-106	4.935723	0.582	0.025	8.25E-102
Osteo	Fgfr2	5.61E-106	1.693455	0.738	0.065	1.07E-101
Osteo	Bglap2	3.66E-102	5.147283	0.582	0.028	7.00E-98
Osteo	Tnc	3.70E-101	2.352153	0.688	0.056	7.09E-97
Osteo	Runx2	1.03E-100	1.258095	0.546	0.021	1.97E-96
Osteo	Alpl	1.33E-98	2.982731	0.73	0.078	2.55E-94
Osteo	Cd200	3.62E-89	2.454558	0.872	0.182	6.94E-85
Osteo	Sp7	1.36E-75	1.152935	0.369	0.008	2.61E-71

Cluster	Gene	p_val	avg_log2FC	pct.1	pct.2	p_val_adj
Chondro	Meltf	1.85E-175	2.485438	0.795	0.005	3.53E-171
Chondro	Ppp1r1b	2.46E-169	1.956606	0.659	0	4.71E-165
Chondro	Mall	3.16E-159	1.639586	0.682	0.003	6.05E-155
Chondro	Hapln1	7.53E-150	2.635117	0.818	0.013	1.44E-145
Chondro	Ucma	4.61E-129	2.381635	0.545	0.002	8.84E-125
Chondro	Cytl1	2.01E-112	6.938956	0.591	0.008	3.86E-108
Chondro	Gdf6	1.06E-110	1.874056	0.432	0	2.02E-106
Chondro	Clec3a	1.12E-102	3.041147	0.591	0.011	2.15E-98
Chondro	Tspan13	5.37E-99	2.384974	0.75	0.027	1.03E-94
Chondro	Cpm	1.81E-89	1.720738	0.614	0.018	3.47E-85

Cluster	Gene	p_val	avg_log2FC	pct.1	pct.2	p_val_adj
Teno	Angptl7	8.10E-60	3.504045	0.422	0.024	1.55E-55
Teno	Uts2r	2.41E-53	1.093415	0.422	0.028	4.62E-49
Teno	Ccdc3	5.85E-53	1.865815	0.878	0.218	1.12E-48
Teno	Fibin	1.27E-43	2.653455	0.744	0.176	2.43E-39
Teno	Dkk3	4.67E-43	1.400572	0.8	0.207	8.95E-39
Teno	Cilp	6.26E-43	2.189124	0.744	0.169	1.20E-38
Teno	Prelp	3.43E-42	2.548272	0.978	0.753	6.56E-38
Teno	Kera	9.54E-39	2.452145	0.356	0.031	1.83E-34
Teno	Dcn	1.30E-37	1.523629	1	0.978	2.49E-33
Teno	Lox	1.12E-36	1.992322	0.767	0.228	2.15E-32

Cluster	Gene	p_val	avg_log2FC	pct.1	pct.2	p_val_adj
F1	S100a10	3.06E-52	1.428255	1	0.982	5.86E-48
F1	Anxa8	8.15E-47	2.119704	0.756	0.216	1.56E-42
F1	Tspo	5.49E-45	1.226833	0.992	0.885	1.05E-40
F1	Crip1	1.34E-42	1.406402	1	0.993	2.57E-38
F1	Ociad2	6.66E-42	1.149411	0.614	0.139	1.28E-37
F1	Anxa2	3.20E-40	1.20036	1	0.974	6.13E-36
F1	Slurp1	2.59E-37	2.127482	0.417	0.062	4.96E-33
F1	Txn1	5.84E-36	1.095678	1	0.931	1.12E-31
F1	Igfbp6	1.30E-34	1.418252	0.992	0.94	2.49E-30
F1	Crip2	1.03E-33	1.159756	0.992	0.87	1.97E-29

Cluster	Gene	p_val	avg_log2FC	pct.1	pct.2	p_val_adj
F2	Cxcl12	9.18E-57	1.642371	0.961	0.671	1.76E-52
F2	Ctsb	5.90E-45	0.857229	0.989	0.954	1.13E-40
F2	Pcolce	5.59E-43	0.952381	1	0.971	1.07E-38
F2	Gpnmb	1.93E-42	1.163044	0.831	0.422	3.70E-38
F2	Gas6	7.44E-42	0.959344	0.965	0.728	1.42E-37
F2	Dcn	5.56E-40	0.748553	1	0.973	1.07E-35
F2	Serping1	1.06E-38	1.067863	0.972	0.883	2.02E-34
F2	Serpina3n	6.33E-38	1.079861	0.803	0.468	1.21E-33
F2	Rarres2	9.36E-36	0.974022	0.986	0.838	1.79E-31

F2	C4b	3.15E-35	0.999104	0.743	0.343	6.03E-31

Cluster	Gene	p_val	avg_log2FC	pct.1	pct.2	p_val_adj
F3	Cxcl1	4.85E-47	2.556798	0.81	0.251	9.28E-43
F3	Lpl	4.23E-40	1.911208	0.871	0.36	8.09E-36
F3	Dpep1	4.80E-37	1.281209	0.75	0.222	9.20E-33
F3	Ccl11	2.16E-34	1.392893	0.681	0.177	4.15E-30
F3	Acvr2a	5.51E-34	1.046518	0.517	0.118	1.06E-29
F3	Ntrk2	2.89E-31	1.136963	0.802	0.314	5.54E-27
F3	Gsn	3.85E-31	1.135511	1	0.991	7.38E-27
F3	Col4a1	1.08E-30	1.632134	0.836	0.441	2.07E-26
F3	Fst	5.93E-28	1.331829	0.698	0.259	1.14E-23
F3	Bmper	3.56E-27	0.782675	0.543	0.143	6.82E-23

Cluster	Gene	p_val	avg_log2FC	pct.1	pct.2	p_val_adj
F4	Pi16	1.55E-109	4.035385	0.923	0.233	2.96E-105
F4	Car8	5.48E-107	1.89606	0.788	0.118	1.05E-102
F4	Efhd1	1.07E-86	1.603401	0.827	0.218	2.04E-82
F4	Anxa3	6.04E-85	2.043907	0.957	0.385	1.16E-80
F4	Aif1l	1.81E-81	1.181406	0.591	0.075	3.47E-77
F4	Mfap5	2.41E-73	1.773895	1	0.685	4.62E-69
F4	Cd248	8.39E-73	1.644116	0.995	0.561	1.61E-68
F4	Tek	2.15E-72	0.819107	0.5	0.047	4.11E-68
F4	Zyx	7.78E-71	1.524139	0.904	0.392	1.49E-66
F4	Sema3c	1.98E-70	1.717605	0.913	0.439	3.79E-66

Cluster	Gene	p_val	avg_log2FC	pct.1	pct.2	p_val_adj
F5	C7	9.63E-52	1.210175	0.324	0.016	1.84E-47
F5	Abcc9	3.85E-38	0.828005	0.324	0.03	7.38E-34
F5	Fmo2	3.56E-35	1.245506	0.342	0.04	6.82E-31
F5	Rbp1	4.40E-34	1.807167	0.568	0.152	8.43E-30
F5	F3	9.75E-33	2.368749	0.541	0.143	1.87E-28
F5	Kcnj8	3.67E-31	1.216805	0.351	0.05	7.03E-27
F5	Sparcl1	5.07E-24	1.738239	0.631	0.234	9.70E-20
F5	Gdf10	1.56E-21	1.546707	0.559	0.194	2.99E-17
F5	Kitl	4.87E-20	1.395803	0.387	0.104	9.33E-16
F5	Cygb	1.34E-19	1.461814	0.802	0.597	2.56E-15

Cluster	Gene	p_val	avg_log2FC	pct.1	pct.2	p_val_adj
F6	Hsd11b1	9.42E-72	1.727112	0.742	0.033	1.80E-67
F6	Vtn	3.15E-58	1.430101	0.581	0.023	6.04E-54
F6	Aldh1a2	1.30E-42	1.670248	0.484	0.024	2.49E-38
F6	Ret	1.95E-37	0.9342	0.484	0.029	3.73E-33
F6	Atp1a2	5.59E-37	1.142874	0.613	0.049	1.07E-32
F6	Cldn15	1.26E-36	0.74603	0.419	0.021	2.42E-32
F6	Ccl11	1.82E-36	3.358332	1	0.206	3.49E-32

F6	Hmcn2	1.27E-35	2.497802	0.968	0.18	2.43E-31
F6	D630033O11Rik	4.49E-35	1.156915	0.548	0.042	8.60E-31
F6	Prss12	8.49E-34	1.087155	0.452	0.029	1.63E-29

Supplementary Table 6. Top 10 DEGs for identified *Prg4*+progenitor and VSMC clusters in steady state.

Cluster	Gene	p_val	avg_log2FC	pct.1	pct.2	p_val_adj
Progenitor	Megf10	3.15E-22	0.756472	0.438	0.024	6.03E-18
Progenitor	Gm12695	2.45E-19	1.138587	0.5	0.038	4.69E-15
Progenitor	Enpp5	1.54E-18	0.767496	0.562	0.05	2.96E-14
Progenitor	Rspo2	9.32E-18	1.405092	0.625	0.064	1.78E-13
Progenitor	Cdkn2c	7.49E-15	0.917494	0.625	0.08	1.43E-10
Progenitor	Pla1a	2.35E-12	1.411035	0.75	0.143	4.51E-08
Progenitor	Marcksl1	7.55E-12	0.730894	0.5	0.062	1.45E-07
Progenitor	Sparcl1	9.54E-11	1.6785	0.938	0.262	1.83E-06
Progenitor	Nat8f1	1.37E-10	0.557503	0.438	0.055	2.62E-06
Progenitor	Pi15	8.59E-10	1.155656	0.812	0.208	1.64E-05

Cluster	Gene	p_val	avg_log2FC	pct.1	pct.2	p_val_adj
VSMCs	Gja4	4.82E-173	2.780155	0.667	0	9.22E-169
VSMCs	Tinagl1	1.30E-148	3.658437	0.667	0.001	2.48E-144
VSMCs	Kcnk3	8.00E-116	1.23995	0.444	0	1.53E-111
VSMCs	Rasgrp2	1.66E-104	1.910893	0.667	0.003	3.18E-100
VSMCs	Mcam	1.30E-103	1.512162	0.556	0.002	2.49E-99
VSMCs	Gucy1b1	3.11E-95	1.689412	0.667	0.004	5.95E-91
VSMCs	Myh11	6.25E-93	3.266787	0.444	0.001	1.20E-88
VSMCs	Timp4	2.88E-87	1.614912	0.333	0	5.51E-83
VSMCs	Nrip2	2.88E-87	1.452769	0.333	0	5.51E-83
VSMCs	Higd1b	2.88E-87	1.359717	0.333	0	5.51E-83

Supplementary Table 7. Top 10 DEGs for each cluster in integrated steady state and injured state.

Cluster	Gene	p_val	avg_log2FC	pct.1	pct.2	p_val_adj
FLS	Prg4	0	4.192193	1	0.423	0
FLS	Hbegf	0	4.118061	0.979	0.204	0
FLS	Rgcc	0	3.800575	0.992	0.352	0
FLS	F13a1	0	3.260149	0.988	0.094	0
FLS	Htra4	0	3.258303	0.985	0.215	0
FLS	Clic5	0	3.141345	0.973	0.041	0
FLS	Col22a1	0	2.66492	0.975	0.073	0
FLS	Cystm1	0	2.427589	0.927	0.077	0
FLS	Tspan15	0	2.312283	0.967	0.068	0
FLS	Pla1a	0	2.094637	0.934	0.177	0

Cluster	Gene	p_val	avg_log2FC	pct.1	pct.2	p_val_adj
Prog	Rspo2	9.41E-115	1.428591	0.724	0.269	1.80E-110
Prog	Nt5dc2	4.44E-94	1.470391	0.769	0.373	8.51E-90
Prog	Pla1a	2.10E-88	1.094779	0.656	0.222	4.01E-84
Prog	Tmem98	9.55E-84	0.79461	0.835	0.475	1.83E-79
Prog	Htra1	2.60E-83	0.96994	0.989	0.911	4.97E-79
Prog	Fn1	1.21E-79	0.974708	1	0.937	2.31E-75
Prog	Ctsb	2.51E-78	0.892483	0.998	0.976	4.80E-74
Prog	Htra4	7.22E-77	0.767449	0.697	0.261	1.38E-72
Prog	Ucp2	1.26E-75	0.935846	0.893	0.552	2.42E-71
Prog	Fbln7	3.06E-75	1.053359	0.947	0.614	5.87E-71

Cluster	Gene	p_val	avg_log2FC	pct.1	pct.2	p_val_adj
Osteochondro	Alpl	6.41E-266	2.46898	0.578	0.037	1.23E-261
Osteochondro	Mmp13	3.25E-260	3.963802	0.559	0.037	6.23E-256
Osteochondro	Fgfr2	2.27E-227	1.44952	0.638	0.07	4.34E-223
Osteochondro	Fmod	3.39E-174	2.439113	0.762	0.15	6.49E-170
Osteochondro	Cd200	3.76E-171	2.072584	0.708	0.138	7.20E-167
Osteochondro	Sp7	9.16E-170	0.823941	0.305	0.012	1.76E-165
Osteochondro	Cdh11	6.05E-162	1.579114	0.759	0.181	1.16E-157
Osteochondro	Ptprd	5.49E-144	1.140735	0.565	0.094	1.05E-139
Osteochondro	Galr2	2.15E-118	0.640802	0.302	0.024	4.11E-114
Osteochondro	1500015O10Rik	7.37E-118	1.860415	0.898	0.382	1.41E-113

Cluster	Gene	p_val	avg_log2FC	pct.1	pct.2	p_val_adj
Chondro	Col2a1	0	6.228617	0.658	0.009	0
Chondro	Snorc	0	4.390878	0.467	0.001	0
Chondro	Clec3a	0	3.836799	0.592	0.004	0
Chondro	Col11a2	0	3.195569	0.592	0.013	0
Chondro	Ucma	0	2.236359	0.525	0.003	0
Chondro	Meltf	0	2.1324	0.592	0.003	0
Chondro	Ppp1r1b	0	1.53759	0.575	0.001	0

Chondro	Cytl1	5.77E-307	6.400734	0.45	0.004	1.10E-302
Chondro	Col9a3	4.71E-303	3.295872	0.475	0.006	9.02E-299
Chondro	Col9a2	4.92E-291	2.698601	0.442	0.005	9.42E-287

Cluster	Gene	p_val	avg_log2FC	pct.1	pct.2	p_val_adj
Teno	Uts2r	1.76E-128	1.244781	0.485	0.042	3.37E-124
Teno	Cilp2	2.20E-126	1.519471	0.455	0.036	4.22E-122
Teno	Angptl7	2.49E-118	2.84857	0.257	0.009	4.77E-114
Teno	Comp	1.57E-77	2.504709	0.988	0.505	3.01E-73
Teno	Crispld2	6.42E-75	1.453325	0.844	0.263	1.23E-70
Teno	Ackr4	2.79E-74	0.706745	0.365	0.042	5.34E-70
Teno	Prelp	3.71E-74	2.416738	0.976	0.631	7.11E-70
Teno	Dcn	2.79E-70	1.942693	1	0.973	5.34E-66
Teno	Kera	2.17E-67	2.468773	0.509	0.098	4.16E-63
Teno	Vipr2	4.71E-65	0.567463	0.335	0.041	9.03E-61

Cluster	Gene	p_val	avg_log2FC	pct.1	pct.2	p_val_adj
F1	S100a10	3.53E-117	1.193676	1	0.986	6.75E-113
F1	Igfbp6	1.22E-94	1.59357	1	0.895	2.33E-90
F1	Anxa8	5.93E-83	1.475672	0.844	0.436	1.14E-78
F1	Crip1	6.88E-82	1.10051	1	0.987	1.32E-77
F1	Tspo	2.04E-80	0.97059	0.986	0.903	3.90E-76
F1	Emp1	1.95E-78	0.960788	0.992	0.871	3.73E-74
F1	Tmsb4x	2.85E-77	0.940177	0.997	0.969	5.45E-73
F1	Tppp3	1.57E-72	1.205264	0.97	0.71	3.01E-68
F1	Prelp	2.07E-72	1.148166	0.953	0.615	3.97E-68
F1	Anxa2	1.63E-71	0.946931	0.997	0.981	3.12E-67

Cluster	Gene	p_val	avg_log2FC	pct.1	pct.2	p_val_adj
F2	Gas6	5.56E-152	1.659649	0.976	0.601	1.06E-147
F2	Apod	1.37E-149	2.091537	0.936	0.407	2.63E-145
F2	Serping1	6.03E-137	1.68314	0.996	0.707	1.15E-132
F2	C3	1.90E-125	1.413437	0.919	0.385	3.63E-121
F2	C4b	5.30E-125	1.364737	0.838	0.327	1.01E-120
F2	Igf1	1.88E-120	1.745494	0.947	0.543	3.60E-116
F2	Cxcl12	9.24E-113	1.821489	0.974	0.654	1.77E-108
F2	C1s1	1.47E-105	1.061509	0.934	0.522	2.82E-101
F2	Cfb	7.50E-105	1.212355	0.746	0.289	1.44E-100
F2	Mgst1	1.39E-104	1.060967	0.934	0.502	2.66E-100

Cluster	Gene	p_val	avg_log2FC	pct.1	pct.2	p_val_adj
F3	Ntrk2	2.05E-161	1.300263	0.768	0.153	3.92E-157
F3	C3	2.63E-142	2.166307	0.971	0.402	5.03E-138
F3	C4b	1.36E-135	1.958287	0.912	0.341	2.60E-131
F3	Gm2564	2.16E-134	1.695882	0.758	0.174	4.14E-130
F3	Ccl19	1.50E-128	1.345777	0.69	0.143	2.88E-124

F3	Entpd2	9.31E-128	1.675424	0.908	0.339	1.78E-123
F3	Apod	1.04E-118	2.003309	0.958	0.426	1.99E-114
F3	Dpep1	2.18E-117	1.22852	0.569	0.106	4.18E-113
F3	Icam1	2.09E-109	1.316798	0.706	0.208	3.99E-105
F3	Ccl11	1.14E-104	1.511605	0.507	0.089	2.18E-100

**Cluster**	**Gene**	**p_val**	**avg_log2FC**	**pct.1**	**pct.2**	**p_val_adj**
F4	Pi16	0	4.214291	0.915	0.15	0
F4	Efhd1	1.81E-246	1.552535	0.74	0.106	3.47E-242
F4	Tek	4.25E-227	0.781211	0.462	0.029	8.13E-223
F4	Scara5	2.34E-191	1.726887	0.904	0.262	4.49E-187
F4	1700019D03Rik	3.12E-177	1.446628	0.858	0.249	5.98E-173
F4	Dpp4	9.11E-168	0.90987	0.555	0.079	1.74E-163
F4	Cadm3	1.42E-164	1.510233	0.883	0.278	2.71E-160
F4	Cd34	4.21E-164	1.879422	0.997	0.616	8.06E-160
F4	Cd248	1.60E-162	1.828725	0.992	0.642	3.06E-158
F4	Zfp385a	2.68E-159	1.504881	0.803	0.246	5.13E-155

**Cluster**	**Gene**	**p_val**	**avg_log2FC**	**pct.1**	**pct.2**	**p_val_adj**
F5	C7	0	2.170172	0.66	0.008	0
F5	Abcc9	2.84E-187	1.41048	0.67	0.037	5.43E-183
F5	Gdf10	5.75E-150	3.003916	0.962	0.127	1.10E-145
F5	Fmo2	1.01E-142	1.706508	0.594	0.041	1.94E-138
F5	F3	7.08E-126	3.102675	0.849	0.121	1.36E-121
F5	Kcnj8	4.94E-125	1.680578	0.585	0.045	9.47E-121
F5	Inmt	1.32E-113	1.143862	0.283	0.009	2.52E-109
F5	C2	3.94E-87	1.428263	0.575	0.068	7.54E-83
F5	Rbp1	6.68E-84	2.321247	0.896	0.223	1.28E-79
F5	Atp1a2	1.95E-73	0.690957	0.33	0.023	3.73E-69

**Cluster**	**Gene**	**p_val**	**avg_log2FC**	**pct.1**	**pct.2**	**p_val_adj**
F6	Ccl11	2.23E-44	2.532614	0.446	0.107	4.27E-40
F6	Abca8a	5.85E-40	1.348558	0.497	0.146	1.12E-35
F6	Hmcn2	5.34E-36	1.667374	0.414	0.113	1.02E-31
F6	Gsn	1.23E-32	1.593795	0.955	0.979	2.35E-28
F6	Myoc	1.86E-25	1.496215	0.522	0.195	3.57E-21
F6	Dpep1	2.79E-23	1.314804	0.382	0.13	5.34E-19
F6	AW112010	8.19E-23	0.991697	0.261	0.064	1.57E-18
F6	Ltbp4	1.13E-22	1.712527	0.713	0.574	2.16E-18
F6	Entpd2	1.57E-20	1.267053	0.624	0.371	3.01E-16
F6	Tmem204	7.97E-20	0.686135	0.255	0.067	1.53E-15

**Cluster**	**Gene**	**p_val**	**avg_log2FC**	**pct.1**	**pct.2**	**p_val_adj**
VSMC	Fabp4	0	5.027236	0.821	0.023	0
VSMC	Tinagl1	0	3.119222	0.917	0.005	0
VSMC	Notch3	0	2.438523	0.881	0.024	0

VSMC	Gja4	0	2.332056	0.75	0.002	0
VSMC	Esam	0	2.312816	0.905	0.004	0
VSMC	Myh11	0	2.242495	0.667	0.002	0
VSMC	Higd1b	0	2.09126	0.738	0	0
VSMC	Bcam	0	1.905123	0.75	0.014	0
VSMC	Mcam	0	1.851284	0.845	0.009	0
VSMC	Gucy1a1	0	1.850627	0.726	0.008	0

**Cluster**	**Gene**	**p_val**	**avg_log2FC**	**pct.1**	**pct.2**	**p_val_adj**
IF1	Rad51ap1	0	0.733068	0.8	0.013	0
IF1	Lrr1	7.02E-259	0.425761	0.55	0.007	1.34E-254
IF1	Mybl2	3.42E-242	0.390336	0.483	0.006	6.55E-238
IF1	Mcm5	5.14E-240	0.867321	0.9	0.031	9.84E-236
IF1	Top2a	1.43E-234	1.455163	0.867	0.029	2.73E-230
IF1	Pbk	1.11E-232	1.226563	0.817	0.025	2.12E-228
IF1	Uhrf1	1.89E-226	0.878371	0.817	0.027	3.61E-222
IF1	Clspn	8.51E-226	0.724775	0.767	0.022	1.63E-221
IF1	Hist1h1b	6.48E-222	0.996719	0.467	0.006	1.24E-217
IF1	Dscc1	1.41E-218	0.593203	0.633	0.015	2.70E-214

**Cluster**	**Gene**	**p_val**	**avg_log2FC**	**pct.1**	**pct.2**	**p_val_adj**
IF2	Gjb5	1.10E-199	0.7378	0.681	0.082	2.10E-195
IF2	Glipr1	3.86E-135	0.625788	0.611	0.097	7.40E-131
IF2	Cenpa	6.24E-135	1.799363	0.509	0.069	1.20E-130
IF2	Pclaf	1.22E-134	0.737422	0.456	0.049	2.34E-130
IF2	Gjb3	1.25E-128	0.310034	0.281	0.015	2.39E-124
IF2	Ccnb2	4.41E-128	1.131655	0.467	0.059	8.45E-124
IF2	Bcat1	6.45E-126	0.797317	0.804	0.204	1.24E-121
IF2	Acta2	7.86E-124	1.101911	0.684	0.141	1.50E-119
IF2	Birc5	1.17E-119	1.189635	0.432	0.053	2.25E-115
IF2	Il1rl1	8.12E-119	1.094216	0.474	0.064	1.55E-114

**Cluster**	**Gene**	**p_val**	**avg_log2FC**	**pct.1**	**pct.2**	**p_val_adj**
IF3	C1qtnf3	6.49E-222	2.919563	0.848	0.201	1.24E-217
IF3	Tnn	1.27E-191	1.422573	0.873	0.205	2.44E-187
IF3	Postn	1.18E-175	2.313526	0.983	0.458	2.25E-171
IF3	Cthrc1	1.09E-166	2.229164	0.99	0.543	2.09E-162
IF3	Col12a1	1.51E-154	1.565005	0.945	0.371	2.89E-150
IF3	Col1a1	1.06E-148	1.543449	1	0.955	2.02E-144
IF3	Col1a2	5.76E-141	1.393105	1	0.874	1.10E-136
IF3	Capn6	8.75E-133	0.759305	0.656	0.155	1.68E-128
IF3	Ptn	9.44E-130	1.660086	0.89	0.353	1.81E-125
IF3	Lgals1	4.07E-127	1.042633	1	0.98	7.80E-123

**Cluster**	**Gene**	**p_val**	**avg_log2FC**	**pct.1**	**pct.2**	**p_val_adj**
IF4	Mmp27	1.19E-98	0.563316	0.5	0.009	2.27E-94

IF4	Col6a5	1.78E-97	1.046239	0.636	0.016	3.41E-93
IF4	Draxin	3.14E-78	0.404489	0.318	0.005	6.00E-74
IF4	Dlk1	9.87E-78	3.36091	0.682	0.025	1.89E-73
IF4	Cpz	1.10E-62	1.508402	0.818	0.048	2.11E-58
IF4	Plac8	1.94E-51	3.420075	1	0.099	3.71E-47
IF4	Wnt16	6.32E-47	0.863145	0.773	0.054	1.21E-42
IF4	Mcoln2	1.23E-45	0.429844	0.409	0.015	2.35E-41
IF4	Ccl8	1.11E-37	2.731665	1	0.13	2.12E-33
IF4	C430049B03Rik	1.71E-36	1.519104	0.682	0.057	3.28E-32

Supplementary Table 8. *Foxm1* Regulon

Cep55	*Racgap1*	*Hmgb2*	*Mxd3*	*Dock5*	*Asf1b*
Mki67	*Ncapd2*	*Ndc80*	*Ska1*	*Tacc3*	

Supplementary Table 9. *Pole3* Regulon

Acaca	*Acbd3*	*Adam12*	*Adcy7*	*Arntl*	*Baz1b*
C2cd3	*C87436*	*Ccbe1*	*Ccne1*	*Chaf1b*	*Chn2*
Fam98a	*Fancl*	*Gart*	*Gemin6*	*Gins2*	*Gpr153*
Hat1	*Hist1h1e*	*Hjurp*	*Hmgb3*	*Hmgcr*	*Ier3ip1*
Irx3	*Lsm3*	*Lsm5*	*Mbtps2*	*Mcm3*	*Mcm4*
Mcm7	*Mdn1*	*Med10*	*Moxd1*	*Myh10*	*Nfkb2*
Ogg1	*Pcdh19*	*Pla2g4a*	*Plk4*	*Poc5*	*Polg*
Ppp1r7	*Ptpn4*	*Racgap1*	*Rpa2*	*Ruvbl2*	*Sephs1*
Skp2	*Slc25a44*	*Smad2*	*Smchd1*	*Snrpd1*	*Steap1*
Tbx3					

Supplementary Table 10. *Pole4* Regulon

Ccne1	*Gins2*	*Mcm3*	*Rbm3*	*Enah*	*Ung*
Gap43	*Dtl*	*Uhrf1*	*Cenpk*	*Mcm2*	*Mcm5*
Orc6	*Pcna*	*Rad51*	*Psat1*	*Siva1*	

Supplementary Table 11. *Hif1a* Regulon

Ankrd11	*Kdm2b*	*190002N15Rik*	*Sgk1*	*Abl2*	*Ankrd17*
Btg3	*Copa*	*Ddx3x*	*Dlc1*	*Gas5*	*Lmna*
Lysmd3	*Mbnl2*	*Nr4a2*	*Prrc2c*	*Rpl3*	*Srpr*
Srsf6	*Stat3*	*Tmem39a*	*Tnks2*	*Uso1*	*Vegfa*
Xpr1	*Socs2*	*Tnfaip3*	*Adcy7*	*Ak2*	*Anp32b*
Arcn1	*Arhgef19*	*Cacna1c*	*Cald1*	*Calu*	*Cblb*
Chn2	*Clip1*	*Cltc*	*Cnnm4*	*Cog3*	*Ddit4*
Dhrs3	*Dnajc1*	*Dock9*	*Dyrk2*	*En1*	*Eri3*
Ext1	*Fam117b*	*Foxp4*	*Gadd45b*	*Golga4*	*H19*
Hnrnpa0					

Supplementary Table 12. *Nfactc4* Regulon

Vegfa	*Angptl2*	*Antxr1*	*C1qtnf6*	*Chd3*	*Dhx57*
Fbn1	*Glis3*	*Kcnj15*	*Lrig3*	*Meg3*	*Nfatc2*

Prrx2	*Psd3*	*Rin3*	*Tgfb3*	*Tnn*	*Vcan*
Col12a1	*Dkk3*	*Igfbp2*	*Kcnma1*	*Nfatc4*	*Olfml3*
Thbs2	*Mtmr11*	*C1qtnf3*	*Prkd2*	*Srpx2*	*Arsj*
Olfml2b	*Hmcn1*	*Itga2*	*Aspn*	*Fzd2*	

Supplementary Table 13. *Cebpd* Regulon

Arhgap32	*C2*	*Cntfr*	*Gpc3*	*Sfrp2*	*Adipoq*
Angpt1	*Cadm3*	*Col6a6*	*Dkk2*	*Fgf7*	*Flrt2*
Fmo1	*Hdac7*	*Mgst1*	*Ntrk2*	*Opcml*	*Ankrd11*
Chd2	*Eif4a2*	*Foxp1*	*Pdp1*	*Spop*	*Tmcc3*
Arid5a	*Arl5b*	*Atf3*	*Atp8b1*	*Btg1*	*Btg2*
Ccl7	*Cxcl1*	*Cyr61*	*Dusp1*	*Fam110b*	*Fos*
Fosb	*Fosl2*	*Gadd45g*	*Gem*	*Ier2*	*Irs2*
Jun	*Kdm6b*	*Klf6*	*Klf9*	*Map3k8*	*Mllt10*
Myc	*Nckap5l*	*Nfil3*	*Nfkbiz*	*Per1*	*Sgk1*
Slc38a2	*Spry2*	*Trib1*			

Supplementary Table 14. *Klf4* Regulon

Cadm3	*Atf3*	*Cdkn1a*	*Fosb*	*Has1*	*Mafk*
Osr2	*Arl4a*	*Insig1*	*Maff*	*Rab11fip5*	*Tob1*
Bdnf	*Camkk1*	*Coro2b*	*Erf*	*Klf3*	*Mtmr12*
Mtmr3	*Ncoa3*	*Pak4*	*Stk40*	*Chst1*	*Tmem158*
Cry2	*Wnt2*	*Rbm38*	*Rusc2*	*Klf13*	*Cd248*
Msx1	*Ndrg1*	*Timp3*	*Trip10*	*Ugdh*	*Zfp385a*
Arap1	*Tacc2*	*Spsb1*	*Mical1*	*Hdac4*	*Scara5*
Stmn4	*Bbc3*				

Supplementary Table 15. *Irf1* Regulon

Cntfr	*Dkk2*	*Tbx5*	*Atf3*	*Ccnl1*	*Irf1*
Klf9	*Map3k8*	*Mllt10*	*Sbno2*	*Zfp36l1*	*Il6*
Cxcl10	*H2-Q7*	*Stx11*	*Tnfaip3*	*Hk2*	*Pogz*
Socs7	*Twist2*	*Acsl1*	*Pparg*	*Nr2f2*	*Psmb8*
Tcirg1	*Pik3r1*	*Tifa*	*Tap2*	*H2-Q6*	*Plxna2*
Deptor	*Rnf19b*	*Cebpd*	*Dtx3l*	*Esr1*	*Nampt*
Pcdh18	*Uba7*				

Supplementary Table 16. *Sox5* Regulon

Col4a1	*Hipk2*	*Limch1*	*Lmo4*	*Pbx1*	*Sox11*
Traf1	*Zeb2*	*Zfp385b*	*Sparcl1*	*Prune2*	*Calcrl*
Mfap3l	*Rtn1*	*Rspo2*	*Col22a1*	*Fut9*	*Hcn1*
Sox5	*Ugp2*	*Efnb2*	*Gpr1*	*Itgb8*	*Clic5*
Pcbd1					

Supplementary Table 17. *Foxo1* Regulon

Dcaf15	*Mrpl53*	*Tet2*	*Usp38*	*Als2*	*Ankrd11*
Ccnk	*Cdc14b*	*Ctnnd1*	*Dnajb4*	*Eif4a2*	*Exoc2*
Fbxo33	*Fbxo42*	*Fbxw7*	*Gpbp1*	*Gripap1*	*Homer1*

Hoxa11	*Jarid2*	*Midn*	*Slc30a9*	*Smchd1*	*Tiprl*
Ubn2	*Zfhx3*	*Zfp503*	*Zfp655*	*Bhlhe40*	*Cttnbp2nl*
Hsph1	*Ier2*	*Junb*	*Klf4*	*Nckap5l*	*Nfkbiz*
Ppp1r10	*Sbno2*	*Trib1*	*810055G02Rik*	*Adipor1*	*Aftph*
Ahnak	*Aldoa*	*Ankrd17*	*Arf6*	*Arhgef10l*	*Arih2*
Arl4a	*BC005537*	*Becn1*			

Supplementary Table 18. *Creb5* Regulon

Abr	*Adamtsl1*	*Ap2a2*	*Arrb1*	*Atp6v0c*	*Bend6*
Casp3	*Cdk6*	*Cdr2l*	*Ckb*	*Clcn3*	*Clcn5*
Creb5	*Daam1*	*Dbndd2*	*Dennd4b*	*Egfr*	*Elmo1*
Epdr1	*Fahd2a*	*Fn1*	*Fyn*	*Gabarapl2*	*Homer1*
Hspb8	*Igfbp5*	*Igsf9b*	*Laptm4b*	*Map1lc3a*	*Mknk2*
Mmp28	*Nacc2*	*Nhs*	*Pfdn1*	*Ppp2r5b*	*Ptpre*
Rab10	*Rela*	*Rnd2*	*Sbsn*	*Sema4c*	*Slc6a9*
Sox11	*Thbd*	*Timp3*	*Tmbim1*	*Tmod1*	*Trps1*
Ubap1	*Vat1*	*Zfhx2*			

References

1 Rountree RB, Schoor M, Chen H, *et al.* BMP receptor signaling is required for postnatal maintenance of articular cartilage. *PLoS Biol* 2004;**2**:e355. doi:10.1371/journal.pbio.0020355

2 Madisen L, Zwingman TA, Sunkin SM, *et al.* A robust and high-throughput Cre reporting and characterization system for the whole mouse brain. *Nat Neurosci* 2010;**13**:133–40. doi:10.1038/nn.2467

3 Snippert HJ, van der Flier LG, Sato T, *et al.* Intestinal Crypt Homeostasis Results from Neutral Competition between Symmetrically Dividing Lgr5 Stem Cells. *Cell* 2010;**143**:134–44. doi:10.1016/j.cell.2010.09.016

4 Roelofs AJ, Zupan J, Riemen AHK, *et al.* Joint morphogenetic cells in the adult mammalian synovium. *Nat Commun* 2017;**8**:15040. doi:10.1038/ncomms15040

5 Eltawil NM, De Bari C, Achan P, *et al.* A novel in vivo murine model of cartilage regeneration. Age and strain-dependent outcome after joint surface injury. *Osteoarthritis Cartilage* 2009;**17**:695–704. doi:10.1016/j.joca.2008.11.003

6 Roelofs AJ, De Bari C. Immunostaining of Skeletal Tissues. In: *Methods in molecular biology (Clifton, N.J.)*. Humana Press 2019. 437–50. doi:10.1007/978-1-4939-8997-3_25

7 Gnerre S, Maccallum I, Przybylski D, *et al.* High-quality draft assemblies of mammalian genomes from massively parallel sequence data. *Proc Natl Acad Sci U S A* 2011;**108**:1513–8. doi:10.1073/pnas.1017351108

8 Young MD, Behjati S. SoupX removes ambient RNA contamination from droplet-based single-cell RNA sequencing data. *Gigascience* 2020;**9**:1–10. doi:10.1093/gigascience/giaa151

9 Butler A, Hoffman P, Smibert P, *et al.* Integrating single-cell transcriptomic data across different conditions, technologies, and species. *Nat Biotechnol* 2018;**36**:411–20. doi:10.1038/nbt.4096

10 La Manno G, Soldatov R, Zeisel A, *et al.* RNA velocity of single cells. *Nature* 2018;**560**:494–8. doi:10.1038/s41586-018-0414-6

11 Bergen V, Lange M, Peidli S, *et al.* Generalizing RNA velocity to transient cell states through dynamical modeling. *Nat Biotechnol* Published Online First: 2020. doi:10.1038/s41587-020-0591-3

12 Cao J, Spielmann M, Qiu X, *et al.* The single-cell transcriptional landscape of mammalian organogenesis. *Nature* 2019;**566**:496–502. doi:10.1038/s41586-019-0969-x

13 Street K, Risso D, Fletcher RB, *et al.* Slingshot: cell lineage and pseudotime inference for single-cell transcriptomics. *BMC Genomics* 2018;**19**:477. doi:10.1186/s12864-018-4772-0

14 Chen Y, Lun ATL, Smyth GK. From reads to genes to pathways: Differential expression analysis of RNA-Seq experiments using Rsubread and the edgeR quasi-likelihood pipeline. *F1000Research* 2016;**5**:1–51. doi:10.12688/F1000RESEARCH.8987.2

15 Aibar S, González-Blas CB, Moerman T, *et al.* SCENIC: Single-cell regulatory network inference and clustering. *Nat Methods* 2017;**14**:1083–6. doi:10.1038/nmeth.4463

16 Gustavsen JA, Pai S, Isserlin R, *et al.* Rcy3: Network biology using cytoscape from within r [version 1; peer review: 2 approved]. *F1000Research* 2019;**8**:1–20. doi:10.12688/f1000research.20887.1

BMJ Publishing Group Limited (BMJ) disclaims all liability and responsibility arising from any reliance placed on this supplemental material which has been supplied by the author(s)

17 Hu H, Miao YR, Jia LH, *et al.* AnimalTFDB 3.0: A comprehensive resource for annotation and prediction of animal transcription factors. *Nucleic Acids Res* 2019;**47**:D33–8. doi:10.1093/nar/gky822

18 Croft AP, Campos J, Jansen K, *et al.* Distinct fibroblast subsets drive inflammation and damage in arthritis. *Nature* 2019;**570**:246–51. doi:10.1038/s41586-019-1263-7

19 Chou CH, Jain V, Gibson J, *et al.* Synovial cell cross-talk with cartilage plays a major role in the pathogenesis of osteoarthritis. *Sci Rep* 2020;**10**:1–14. doi:10.1038/s41598-020-67730-y

20 Mizoguchi F, Slowikowski K, Wei K, *et al.* Functionally distinct disease-associated fibroblast subsets in rheumatoid arthritis. *Nat Commun* 2018;**9**:1–11. doi:10.1038/s41467-018-02892-y

21 Zhang F, Wei K, Slowikowski K, *et al.* Defining inflammatory cell states in rheumatoid arthritis joint synovial tissues by integrating single-cell transcriptomics and mass cytometry. *Nat Immunol* 2019;**20**:928–42. doi:10.1038/s41590-019-0378-1

22 Schumacher BL, Block JA, Schmid TM, *et al.* A novel proteoglycan synthesized and secreted by chondrocytes of the superficial zone of articular cartilage. *Arch Biochem Biophys* 1994;**311**:144–52. doi:10.1006/abbi.1994.1219

23 Zhang C, Gao Y, Jadhav U, *et al.* Creb5 establishes the competence for Prg4 expression in articular cartilage. *Commun Biol* 2021;**4**:332. doi:10.1038/s42003-021-01857-0

24 Khan IM, Salter DM, Bayliss MT, *et al.* Expression of clusterin in the superficial zone of bovine articular cartilage. *Arthritis Rheum* 2001;**44**:1795–9. doi:10.1002/1529-0131(200108)44:8<1795::AID-ART316>3.0.CO;2-K

25 Li L, Newton PT, Bouderlique T, *et al.* Superficial cells are self-renewing chondrocyte progenitors, which form the articular cartilage in juvenile mice. *FASEB J* 2017;**31**:1067–84. doi:10.1096/fj.201600918R

26 Buechler MB, Pradhan RN, Krishnamurty AT, *et al.* Cross-tissue organization of the fibroblast lineage. *Nature* 2021;**593**:575–9. doi:10.1038/s41586-021-03549-5
